# Nano-granulated zoledronate sensitizes innate immune metabolism to enhance vaccine-induced and antitumor immunity

**DOI:** 10.1016/j.xcrm.2026.102765

**Published:** 2026-04-22

**Authors:** Meifang Chen, Xiaojia Jiao, Zhicheng Yan, Yueheng Wang, Jing Zhang, Qinghua Wu, Minghui Li, Shumin Fan, Yuan Wang, Wenbing Dai, Hua Zhang, Xueqing Wang, Qiang Zhang, Bing He

**Affiliations:** 1 State Key Laboratory of Natural and Biomimetic Drugs, Peking University, Beijing 100191, China; 2 Beijing Key Laboratory of Molecular Pharmaceutics and New Drug Delivery Systems, School of Pharmaceutical Sciences, Peking University, Beijing 100191, China; 3 Peking University Hospital of Stomatology, Beijing 100191, China

**Keywords:** nano-adjuvants, bisphosphonate, the mevalonate pathway, innate immunity, metabolic regulation

## Abstract

Metabolic reprogramming targeting the mevalonate pathway represents an emerging innate immune activation target. However, its regulatory mechanisms remain incompletely elucidated. Here, we target the mevalonate pathway and construct a nano-granulated zoledronate (Nano-ZD) modulator. Following subcutaneous injection, Nano-ZD preferentially accumulates in draining lymph nodes rather than in bone tissues, enabling targeted delivery to innate immune cells. Nano-ZD functions as an immune-metabolic adjuvant, sensitizing and amplifying immune responses. By integrating Nano-ZD with the TLR4 agonist monophosphoryl lipid A (MPLA), MPLA-loaded Nano-ZD (Nano-ZDM) elicits robust humoral and antitumor cellular immunity. Mechanistically, Nano-ZD not only inhibits the isoprenylation of RhoA GTPases but also reduces coenzyme Q (CoQ) biosynthesis. CoQ deficiency disrupts oxidative phosphorylation (OXPHOS) and pyrimidine metabolism, causes mitochondrial ROS accumulation, induces mitochondrial antiviral protein (MAVS) oligomerization, and activates the pyrin inflammasome. This mevalonate-CoQ-OXPHOS/pyrimidine metabolism axis serves as a promising target for screening additional immune-metabolic adjuvants, and nanofabrication offers a paradigm for the lymph-targeted *in vivo* delivery of such adjuvants.

## Highlights

• Nano-ZD redirects zoledronate to lymph node innate immune cells *in vivo*
• Nano-ZD enhances humoral and antitumor immunity
• Nano-ZDM shows combinatorial antitumor efficacy with αPD-L1 *in vivo*
• The MVA-CoQ-OXPHOS/pyrimidine axis is a potential target for adjuvant discovery

## Graphical abstract

**Figure:**
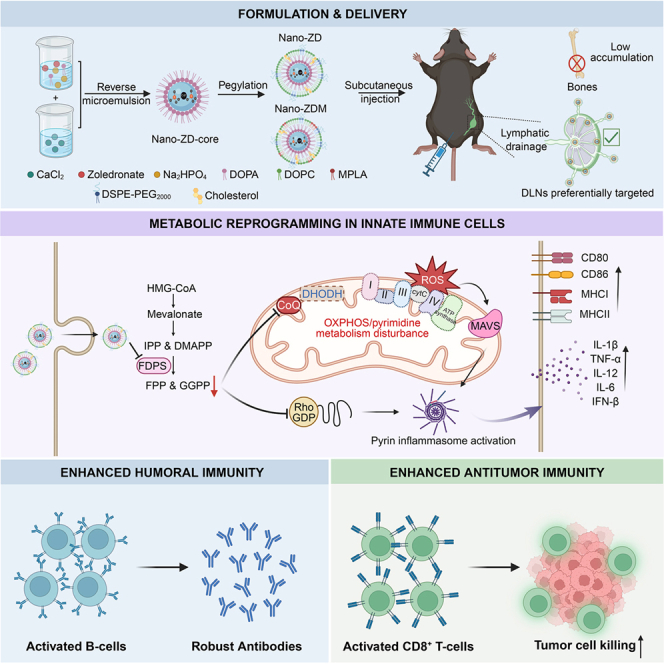

## Teaser

Chen et al. develop a nano-granulated metabolic modulator that redirects zoledronate to lymph nodes, where it sensitizes and amplifies innate immunity to enhance humoral and antitumor responses and identifies the MVA-CoQ-OXPHOS/pyrimidine metabolism axis as a target for immune-metabolic vaccine adjuvant design.

## Introduction

Metabolic reprogramming regulates immune-cell maintenance, differentiation, maturation, activation, and senescence.^1^^,^^2^^,^^3^^,^^4^^,^^5^ Switching among metabolic pathways facilitates innate immune activation.^6^^,^^7^ Upon binding of pathogen-associated molecular patterns (PAMPs) to pattern-recognition receptors (PRRs), innate immune cells undergo activation accompanied by a shift from oxidative phosphorylation (OXPHOS) to aerobic glycolysis.^8^^,^^9^ Studies have demonstrated that altering specific metabolic fluxes can activate innate immune cells independently of PRR stimulation.^10^^,^^11^^,^^12^^,^^13^ These findings suggest that regulators inducing specific metabolic reprogramming could serve as vaccine adjuvants akin to ligands for Toll-like receptors (TLRs), NOD-like receptors (NLRs), C-type lectin receptors, etc.^14^^,^^15^ However, unlike PRRs that are selectively expressed on innate immune cells, most metabolic pathways are conserved across immune and nonimmune cells. Modulating specific metabolic pathways affects not only immune cells but also nonimmune cells. Therefore, achieving specific metabolic reprogramming in innate immune cells remains a significant challenge for developing metabolism-based adjuvants.

Which metabolic pathway is more conducive to innate immune response regulation? This is another critical issue in immune-metabolic adjuvant studies. Such a pathway should affect immune signal transmission, transcription factor activation, cytokine production, and even antigen uptake, processing, and presentation. The mevalonate (MVA) pathway, which is derived from acetyl-CoA, is a multi-branched pathway involved in the synthesis of cholesterol, coenzyme Q (CoQ), and heme, as well as in the regulation of sterol metabolism-related gene expression and small GTPase isoprenylation.^16^^,^^17^^,^^18^ Cholesterol synthesis and its transcriptional regulation in immune cells affect intracellular immune signal transduction through NF-κB and IRF pathways.^19^^,^^20^ Isoprenylation of Rab GTPases is further demonstrated to regulate antigen uptake and presentation by innate immune cells.^21^ Therefore, MVA pathway has been defined as a druggable target for vaccine adjuvant discovery. Nevertheless, given its branched architecture, it remains unclear whether the inhibition of this metabolism can modulate innate immunity through alternative branches.

In this study, we targeted farnesyl diphosphate synthase (FDPS) in the MVA pathway and selected zoledronate, a representative bisphosphonate, as a potential immune-metabolic adjuvant molecule because of its inhibitory effects on this enzyme.^22^ Unlike statins, which act upstream of the MVA pathway, zoledronate inhibits synthesis of farnesyl pyrophosphate (FPP) and geranylgeranyl pyrophosphate (GGPP) by acting midstream. The precise mechanism by which zoledronate modulates intracellular immune signaling via MVA metabolism remains unclear. Additionally, zoledronate, an approved drug for treating osteoporosis and cancer bone metastasis, exhibits strong bone tropism *in vivo*,^23^ which complicates targeted delivery to the innate immune cells.

Here, to address the challenge of targeting delivery of zoledronate and elucidate its detailed molecular mechanisms as the potential immune-metabolic adjuvant, we employed nanotechnology to fabricate nano-granulated zoledronate (Nano-ZD) by coordinating zoledronate with calcium ions using phospholipids as stabilizing agents. Owing to its nanoscale dimensions, Nano-ZD can be delivered via lymphatic vessels to lymph nodes rather than accumulating in bone tissue following subcutaneous injection, thereby facilitating the uptake and activation of antigen-presenting cells. Moreover, our findings indicate that zoledronate and Nano-ZD can sensitize and amplify the innate immune response elicited by classical PAMPs, such as lipopolysaccharide (LPS). To enhance the adjuvant effect, we integrated TLR4 ligand monophosphoryl lipid A (MPLA) with Nano-ZD to prepare Nano-ZDM (MPLA-loaded Nano-ZD). Accompanied by tumor or viral antigens, Nano-ZDM effectively elicits cellular immunity against tumors and induces virus-specific humoral immune responses. Mechanistically, we elucidated a new molecular mechanism through which the MVA pathway regulates innate immunity. In addition to blocking RhoA GTPase isoprenylation, we discovered that both zoledronate and Nano-ZD reduce CoQ levels in cells and mitochondria, leading to the disruption of OXPHOS and pyrimidine metabolism. These metabolic alterations induce mitochondrial reactive oxygen species (mtROS) production and trigger mitochondrial antiviral protein (MAVS) oligomerization. Ultimately, these changes, together with PAMPs, trigger NF-κB signaling, further upregulating pyrin inflammasome expression, thereby sensitizing and amplifying the innate immune response. In conclusion, this study defines a new target based on the MVA-CoQ-OXPHOS/pyrimidine mechanism axis for the screening of more immune-metabolic vaccine adjuvants and provides a nanotechnology-based design paradigm for their druggability.

## Results

### Free zoledronate stimulates and sensitizes innate immune response *in vitro* but not *in vivo*

**Figure 1:**
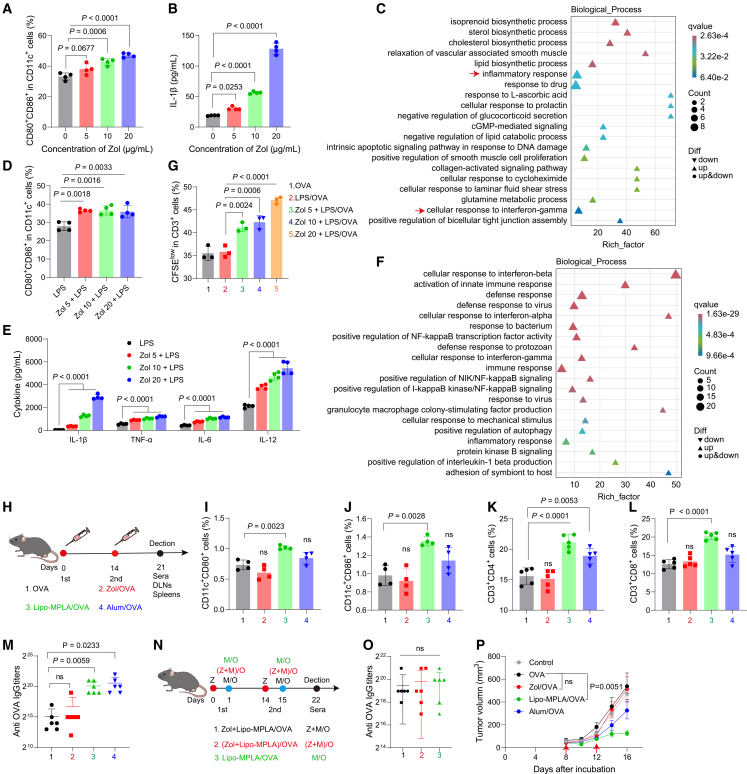
Free zoledronate stimulates and sensitizes innate immune response *in vitro* but not *in vivo* (A) CD80^+^CD86^+^ percentage in CD11c^+^ cells by flow cytometry (*n* = 4). (B) IL-1β in BMDC supernatants by ELISA (*n* = 4). (C) GO enrichment of upregulated genes in BMDCs. Control, PBS; experimental, zoledronate. Red arrows indicate inflammation-related pathways (*n* = 3). (D) CD80^+^CD86^+^ percentage in CD11c^+^ cells by flow cytometry (*n* = 4). (E) IL-1β, TNF-α, IL-6, and IL-12 levels by ELISA (*n* = 4). (F) GO enrichment of upregulated genes in BMDCs. Control, LPS; experimental, Zol + LPS (*n* = 3). (G) CFSE^low^ percentage in CD3^+^ T cells (*n* = 3). (H) Immunization scheme of free zoledronate *in vivo*. (I and J) CD11c^+^CD80^+^ and CD11c^+^CD86^+^ DC percentages in DLNs (*n* = 4). (K and L) CD3^+^CD4^+^ and CD3^+^CD8^+^ T cell percentages in spleens (*n* = 5). (M) OVA-specific total IgG titers (*n* = 6). (N) Immunization scheme of free zoledronate and Lipo-MPLA *in vivo*. (O) OVA-specific total IgG titers (*n* = 6). (P) Tumor growth of B16-OVA tumor-bearing mice (*n* = 6). *n* indicates biologically independent samples. Data are shown as mean ± SEM for (P) and mean ± SD for other panels. Statistical significance was calculated by one-way ANOVA with Tukey’s test. *p* < 0.05 was considered significant.

First, we evaluated the immunostimulatory efficacy of free zoledronate in bone marrow-derived dendritic cells (BMDCs) and bone marrow-derived macrophages (BMDMs) and incubating them with different concentrations of zoledronate (Figures 1 and S1). The dosing concentrations did not cause significant cytotoxicity (Figures S1A and S1B). Flow cytometry showed that free zoledronate upregulated costimulatory molecules CD80 and CD86 in BMDCs (Figures 1A and S3A). It also facilitated the secretion of the proinflammatory factors IL-1β and TNF-α in a concentration-dependent manner but did not affect other proinflammatory cytokines (Figures 1B and S1C–S1E). Gene Ontology (GO) enrichment analysis of RNA sequencing (RNA-seq) data revealed that free zoledronate not only altered isoprenoid and sterol biosynthesis because of its inhibition of MVA metabolism but also induced inflammatory responses to IFN-γ in BMDCs (Figures 1C and S2A–S2C). These findings demonstrated the capacity of free zoledronate to stimulate innate immunity.

Notably, when LPS was added following free zoledronate treatment, the immunostimulatory effect of LPS was markedly increased. Compared with LPS alone, free zoledronate pretreatment increased the proportion of activated BMDCs (Figures 1D and S3B). Moreover, free zoledronate significantly promoted the production of LPS-induced proinflammatory cytokines, including IL-1β, TNF-α, IL-6, and IL-12 (Figure 1E). Western blot analysis further showed increased pro-IL-1β expression in BMDCs (Figure S1F). Compared with six other bisphosphonates, zoledronate maximally increased the expression of IL-1β at the same molar concentration (Figure S1G). Zoledronate also showed similar immunostimulatory effects in BMDMs (Figures S1H–S1K). GO enrichment analysis and gene set enrichment analyses (GSEA) of RNA-seq revealed that free zoledronate plus LPS markedly upregulated genes involved in type I and II interferons, NF-κB, and NLR and RIG-1-like receptor signaling pathways (Figures 1F and S2D–S2F). These findings indicate that zoledronate sensitizes LPS-triggered innate immune responses. Moreover, when BMDCs treated with LPS and the model antigen ovalbumin (OVA) were incubated with mouse splenocytes, the addition of free zoledronate accelerated the proliferation of CD3^+^ T cells (Figures 1G and S3C). These findings suggested that free zoledronate could further enhance adaptive immunity by sensitizing the innate immune response.

Next, we investigated the immunostimulatory efficacy of free zoledronate *in vivo* (Figure 1H). Flow cytometry showed that free zoledronate did not increase dendritic cell activation in draining lymph nodes (DLNs) or T cell proliferation in the spleen. In contrast, MPLA liposomes (Lipo-MPLA) and aluminum adjuvants (Alum) significantly activated dendritic cells in DLNs and increased CD8^+^ or CD4^+^ T cell proportions in the spleen (Figures 1I–1L and S3D). Free zoledronate also failed to increase serum OVA-specific IgG levels compared with Lipo-MPLA and Alum (Figure 1M). The combination of free zoledronate and MPLA also did not have any advantage in inducing humoral immune responses (Figures 1N and 1O). In B16-OVA tumor-bearing mice receiving adjuvant/OVA formulations, free zoledronate did not suppress tumor growth (Figures 1P and S1L–S1N). Collectively, these results suggested that free zoledronate lost its stimulatory and sensitizing effects on immunity *in vivo*.

### Nano-granulated zoledronate is fabricated through coordination interactions and endocytosed by innate immune cells

**Figure 2:**
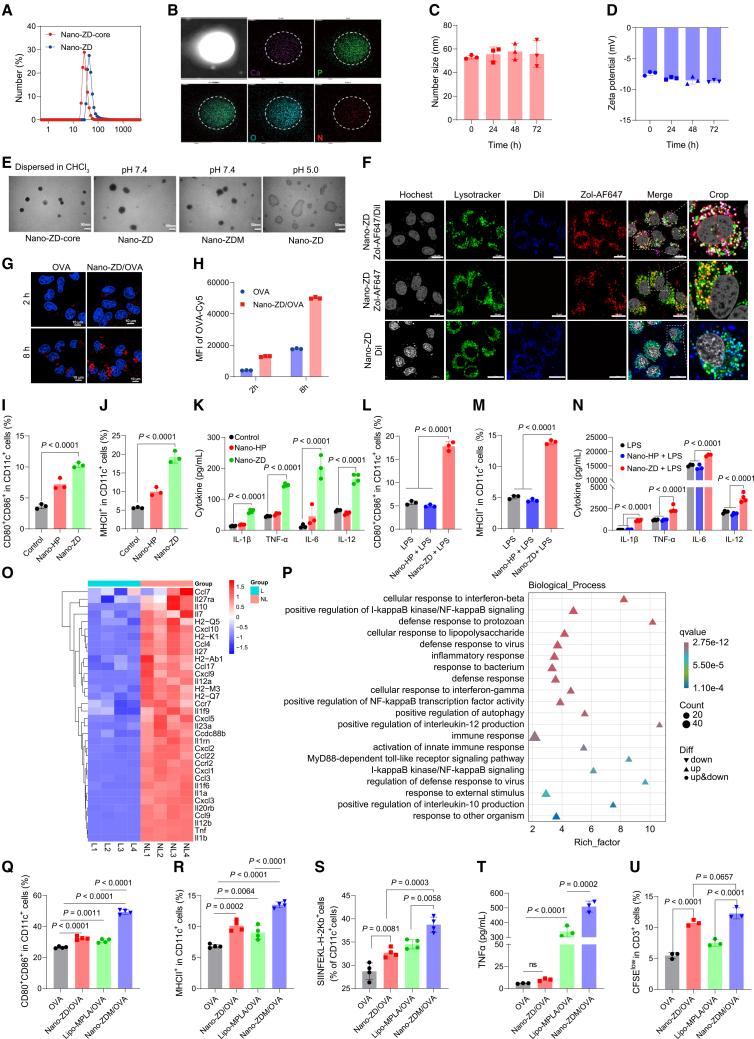
The construction and immunostimulatory effects of Nano-ZD and Nano-ZDM *in vitro* (A) Particle size distribution of Nano-ZD-core and Nano-ZD by DLS. (B) EDS analysis of Nano-ZD-core. (C and D) Size (C) and zeta potential (D) stability of Nano-ZD (*n* = 3). (E) Representative TEM images of Nano-ZD-core, Nano-ZD, and Nano-ZDM. Scale bars, 50 nm. (F) Confocal images of DC2.4 cells incubated with Nano-ZD/Zol-AF647/Dil, Nano-ZD/Zol-AF647, or Nano-ZD/Dil. Scale bars, 10 μm. (G) Confocal images of DC2.4 cells incubated with Nano-ZD/OVA_Cy5_ or OVA _Cy5_. Scale bars, 10 μm. (H) Flow cytometric analysis of Nano-ZD/OVA_Cy5_ or OVA_Cy5_ uptake (*n* = 3). (I and J) CD80^+^CD86^+^ (I) and MHC II^+^ (J) percentages in BMDCs after treatment with Nano-ZD or Nano-HP (*n* = 3). (K) IL-1β, TNF-α, IL-12, and IL-6 levels by ELISA (*n* = 3–4). (L and M) CD80^+^CD86^+^ (L) and MHC II^+^ (M) percentages in BMDCs after Nano-ZD plus LPS treatments (*n* = 3). (N) IL-1β, TNF-α, IL-12, and IL-6 levels by ELISA (*n* = 3–4). (O and P) Heatmap of DEGs involved in innate immune activation and defense response in BMDCs. Control, LPS (L); experimental, Nano-ZD + LPS (NL) (*n* = 4). (P) GO enrichment of upregulated genes in BMDCs. (Q and R) CD80^+^CD86^+^ (Q) and MHC II^+^ (R) percentages in BMDCs after treatments with Nano-ZD/OVA or Nano-ZDM/OVA (*n* = 4). (S) SIINFKEL-H2Kb^+^ percentage in BMDCs (*n* = 4). (T) TNF-α levels by ELISA (*n* = 3). (U) CFSE^low^ percentage in CD3^+^ T cells (*n* = 3). *n* indicates biologically independent samples. Data are shown as mean ± SD. Statistical significance was calculated by one-way ANOVA with Tukey’s test. *p* < 0.05 was considered significant.

The inability of achieving targeting delivery to innate immune cells is the main reason for the loss of immune stimulation and sensitization of free zoledronate *in vivo*. Owing to its hydrophilicity and bone tropism, subcutaneously injected zoledronate rapidly enters systemic circulation and preferentially accumulates in bone tissue rather than secondary lymphoid organs such as lymph nodes, where immune responses are initiated.^23^ To overcome this limitation, we constructed Nano-ZD via nanoprecipitation technology based on methods described in our previous works.^24^^,^^25^^,^^26^ Zoledronate was coordinated with Ca^2+^ cations to form a hydrophobic nanoscale core with the aid of phosphatidic acid. Dynamic light scattering (DLS) showed a hydrodynamic diameter of 32.2 ± 2.0 nm (Figure 2A; Table S1). Energy-dispersive X-ray spectroscopy (EDS) demonstrated colocalization of calcium, oxygen, phosphorus, and nitrogen (specific for zoledronate), confirming homogeneous zoledronate distribution (Figure 2B). The nano-core was further coated with a layer of phospholipids to enable stable dispersion of Nano-ZD in aqueous solution. After lipid coating, the diameter increased to 53.2 ± 1.6 nm. The loading efficiency of zoledronate was 62.67% ± 2.51% measured by high-performance liquid chromatography (HPLC), and the zeta potential was −7.4 ± 0.3 mV. Nano-ZD remained stable in water for 3 days (Figures 2C and 2D) and maintained size and drug retention under physiologically relevant conditions (Figures S4A–S4D). Transmission electron microscopy (TEM) revealed that both the nano-core and the complete Nano-ZD exhibited spherical morphology with the similar size as DLS results. When the pH decreased from 7.4 to 5.0, the Nano-ZD structure was disrupted (Figures 2E and S4E). Consistently, *in vitro* zoledronate release from Nano-ZD at pH 5.0 was more than 2-fold higher than at neutral pH (Figure S4F). This change in pH ensured that Nano-ZD could be degraded to release the loaded zoledronate after Nano-ZD was internalized by innate immune cells. To achieve synergistic adjuvant activity, MPLA was incorporated into Nano-ZD to generate Nano-ZDM by coating the nano-core with a mixture of MPLA and phospholipids. Nano-ZDM exhibited particle size, morphology, and structural characteristics similar to Nano-ZD (Table S1).

Next, we evaluated Nano-ZD uptake by innate immune cells. AF647-conjugated zoledronate (Zol-AF647) labeled the Nano-ZD core, while Dil labeled the lipid layer. After incubation with DC2.4 cells, Nano-ZD was efficiently internalized and colocalized with lysosomes. Nano-ZD maintained its intact structure during the endocytosis process, with good colocalization between the core and the lipid layer (Figures 2F and S4G–S4H). Notably, when DC2.4 cells were incubated with Nano-ZD combined with OVA, the cellular uptake of OVA was markedly increased compared with OVA alone (Figures 2G and 2H). The increased OVA uptake may result from antigen adsorption on the surface of Nano-ZD, which facilitates the internalization of OVA and benefits subsequent processing and presentation.

### Nano-granulated zoledronate maintains the stimulatory and sensitizing effects on innate immune cells *in vitro*

The immunostimulatory efficacy of Nano-ZD was evaluated via the same methods used for free zoledronate. Nano-ZD did not cause significant toxicity to BMDCs and BMDMs at the dosing concentrations (Figures S4I and S4J). Nano-HP, generated by replacing zoledronate with Na_2_HPO_4_ during preparation, served as a reference. Compared with Nano-HP, Nano-ZD significantly upregulated costimulatory molecules (CD80 and CD86) and MHC II expression in BMDCs (Figures 2I–2J and S6A–S6C) and increased production of IL-1β, TNF-α, IL-6, and IL-12 (Figure 2K). Furthermore, GO enrichment analysis and GSEA of the RNA-seq data revealed that Nano-ZD treatment activated multiple immune regulatory pathways, including the defense response, inflammatory response, NF-κB signaling pathway, and protein processing in the endoplasmic reticulum (Figures S5A–S5C). These findings indicated that Nano-ZD, similar to free zoledronate, could maintain or even augment the innate immune response.

Next, we investigated the sensitization effect of Nano-ZD on innate immunity. Like free zoledronate, Nano-ZD pretreatment increased the proportion of activated BMDCs following LPS stimulation and enhanced LPS-induced cytokine secretion, whereas Nano-HP showed no synergistic effect (Figures 2L, 2M, and S6A–S6C). Transcriptome analysis further showed that cytokines and chemokines associated with immune activation, including IL-7, IL-23a, CCL7, and CXCL10, were markedly upregulated following combined Nano-ZD and LPS treatment. These immune-related genes are involved in multiple signaling pathways, including the type I and II interferons, NF-κB, and MyD88-dependent TLR pathways (Figures 2O, 2P, and S5D–S5F). Nano-ZD also produced similar immunostimulatory effects in BMDMs (Figures S4M–S4Q). These results indicated that Nano-ZD sensitizes LPS-triggered innate immune responses similarly to free zoledronate.

As a critical component of vaccines, the efficacy of adjuvants cannot be comprehensively assessed until they are integrated with antigens to constitute a complete vaccine system. We combined Nano-ZD or Nano-ZDM with OVA to investigate their immunostimulatory effects on BMDCs. Nano-ZD had a stimulating effect comparable to Lipo-MPLA (Figures 2Q–2R). Nano-ZD also increased the proportion of BMDCs presenting OVA-specific epitopes to levels close to Lipo-MPLA, although induction of several proinflammatory cytokines was slightly weaker (Figures 2S, 2T, and S4K–S4L). These results highlighted the adjuvant effect of Nano-ZD. Nano-ZDM, which combines Nano-ZD with MPLA, further increased BMDC activation and proinflammatory cytokine production compared with Nano-ZD or Lipo-MPLA. Moreover, it facilitated the generation of more OVA-specific BMDCs and accelerated T cell proliferation (Figures 2Q–2U and S6D–S6G). Furthermore, when BMDCs were co-cultured with spleen lymphocytes from OT-I mice, both free zoledronate and the nano-formulations (Nano-ZD/OVA and Nano-ZDM/OVA) increased CFSE-low CD8^+^ T cells (Figure S7). These results indicated that Nano-ZD could exert synergistic effects in combination with other adjuvants by stimulating and sensitizing innate immune responses.

### Nano-granulated zoledronate achieves the targeting delivery to innate immune cells in lymph nodes

**Figure 3:**
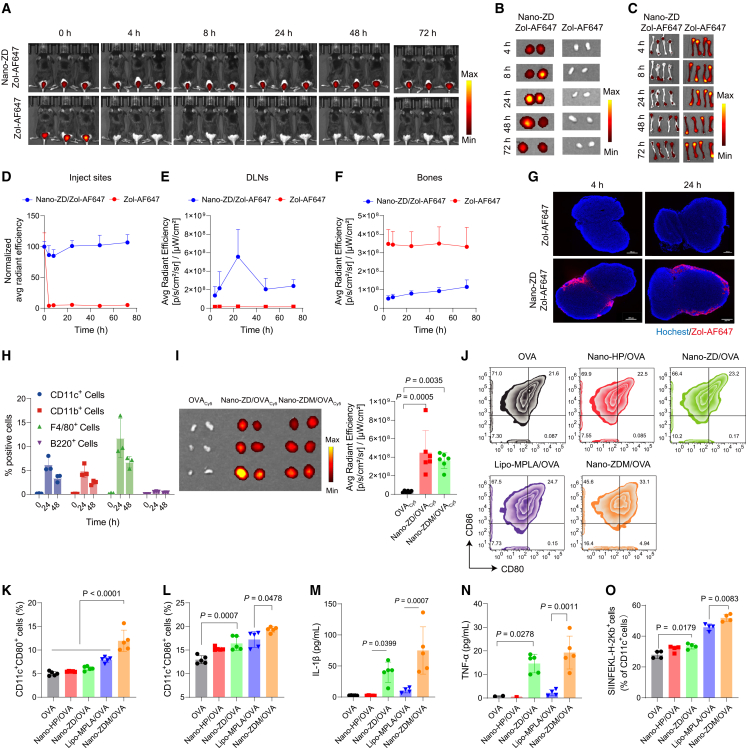
Nano-granulated zoledronate achieves the targeted delivery to innate immune cells in lymph nodes (A) Representative fluorescence images of mice injected with free Zol-AF647 or Nan-ZD/Zol-AF647. (B and C) Fluorescence images of DLNs (B) and bones (C) at different times post injection. (D–F) Average (Avg) radiant efficiency at injection sites, DLNs, and bones (*n* = 3). (G) Representative fluorescence images of DLN sections after treatments with Zol-AF647 or Nano-ZD/Zol-AF647. Scale bars, 300 μm. (H) Flow cytometric analysis of Nano-ZD/Zol-AF647 uptake in DLNs (*n* = 3). (I) Fluorescence images and quantification of DLNs after treatments with OVA_Cy5_, Nano-ZD/OVA_Cy5_, or Nano-ZDM/OVA_Cy5_ (*n* = 3). (J–O) DLNs from treated mice were harvested and prepared into single-cell suspensions. CD11c^+^CD80^+^ (K), CD11c^+^CD86^+^ (L), and SIINFKEL-H2Kb^+^ (O) percentages were analyzed by flow cytometry (*n* = 4–5). IL-1β (M) and TNF-α (N) in the supernatants were detected by ELISA (*n* = 5). *n* indicates number of mice. Data are shown as mean ± SD. Statistical significance was calculated by one-way ANOVA with Tukey’s test. *p* < 0.05 was considered significant.

Achieving targeting delivery to innate immune cells *in vivo* is the core challenge for the efficacy of immune-metabolic adjuvants. Here, we investigated the biodistribution of Nano-ZD in mice after subcutaneous injection by loading Zol-AF647 into the nano-granules. Small animal imaging showed that Nano-ZD significantly prolonged the retention time of zoledronate at the injection site and facilitated its delivery to DLNs compared with free Zol-AF647. Correspondingly, less Nano-ZD was present in the bones than free zoledronate after subcutaneous injection (Figures 3A–3F). Fluorescence quantification further demonstrated that free zoledronate predominantly accumulated in bone, with fluorescence intensity an order of magnitude higher than other tissues (Figures S8A and S8B). Additionally, we determined the concentration of free zoledronate in bones and lymph nodes using HPLC, which further validated the imaging findings (Figures S8C and S8D). These findings demonstrated that nanotechnology enables lymph node targeting of zoledronate, which is a prerequisite for its immune-metabolic adjuvant effect.

Next, we explored the delivery of Nano-ZD to DLNs in detail. The whole-mount microscopy images revealed that Nano-ZD localized mainly to the peripheral region of lymph nodes, where lymphatic vessels enter and myeloid cells congregate^27^ (Figures 3G and S9A). By performing a lymphocyte subset analysis via flow cytometry, we further revealed that Nano-ZD was greatly internalized by the mononuclear phagocyte (MNP) system (Figures 3H and S9B). The MNP population included dendritic cells (CD11c^+^) and macrophages (CD11b^+^ and F4/80^+^), which are involved in the innate immune response and antigen presentation. In contrast, B220^+^ B lymphocytes hardly ingested Nano-ZD. These results suggested that Nano-ZD achieved the targeting delivery to innate immune cells in the lymph nodes. Furthermore, we constructed a fluorescence resonance energy transfer (FRET)-based method to monitor the integrity of Nano-ZD during its transport to DLNs. Nano-ZD_Cy3/AF647_ exhibited an efficient FRET effect from Cy3 to AF647, and when the core of Nano-ZD was dissolved in acidic medium, the FRET effect significantly decreased, demonstrating correlation between FRET efficiency and nanoparticle integrity (Figure S10A). The fluorescence emission spectra and fluorescence lifetime imaging microscopy (FLIM) of DLN sections after Nano-ZD_Cy3/AF647_ injection demonstrate that Nano-ZD can be transported to DLNs in its intact form to exert its biological functions (Figure S10B).

Codelivery of adjuvants and antigens to lymphoid organs is crucial for the efficacy of vaccines. Herein, we investigated whether the targeting delivery of Nano-ZD to DLNs facilitates antigen transport. Fluorescence imaging of the lymph nodes indicated that both Nano-ZD and Nano-ZDM promoted the delivery of OVA antigen to DLNs (Figures 3I and S9C). This codelivery feature is conducive to the processing and presentation of antigens by innate lymphocytes. More importantly, it ensures that the efficacy of zoledronate as an immune-metabolic adjuvant can be achieved *in vivo*. By harvesting and analyzing dendritic cells in DLNs, we demonstrated that Nano-ZD significantly upregulated the expression of costimulatory molecules and promoted the production of the proinflammatory cytokines IL-1β and TNF-α (Figures 3J–3N). Transcriptome analysis further showed activation of myeloid dendritic cells and IL-2 biosynthesis in DLNs (Figures S11A and S11B). Notably, Nano-ZDM exhibited the strongest *in vivo* immunostimulatory activity. Compared with Nano-ZD and Lipo-MPLA, Nano-ZDM enriched more OVA-specific DCs in DLNs (Figures 3O and S11E). GO analysis revealed that Nano-ZDM activated more immune regulatory pathways in DLNs (Figures S11C and S11D). Collectively, these results demonstrated that Nano-ZD, but not free zoledronate, could exert an adjuvant effect *in vivo* through the targeted delivery and stimulation of innate immune cells in DLNs.

### Nano-granulated zoledronate effectively activates humoral immune responses

**Figure 4:**
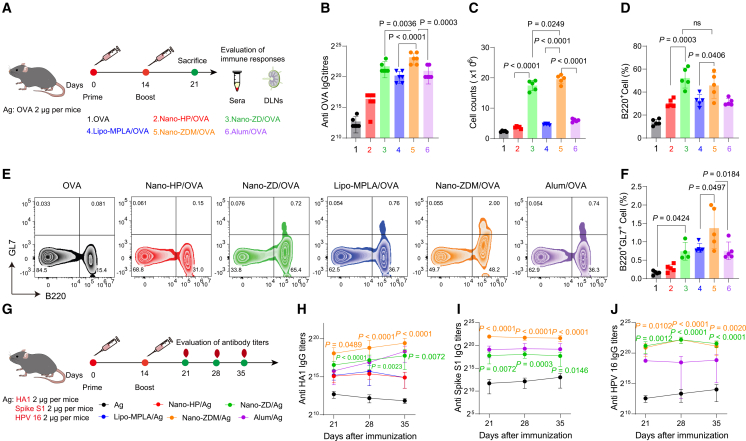
Nano-granulated zoledronate effectively activates humoral immune responses (A) Immunization scheme for indicated groups. (B) OVA-specific total IgG titers (*n* = 6). (C) Total cell counts in DLNs (*n* = 5). (D) Percentage of B220^+^ cells in DLNs (*n* = 5). (E and F) Representative flow cytometry plots and quantification of B220^+^GL7^+^ GC B cells in DLNs (*n* = 4–5). (G–J) Antibody responses to multiple antigens. (G) Immunization schedule. Mice (*n* = 6) were immunized on day 0 and boosted on day 14; serum IgG was measured on days 21, 28, and 35. (H) HA 1. (I) Spike S1. (J) HPV 16. *n* indicates number of mice. Data are shown as mean ± SD. Statistical significance was calculated by one-way ANOVA with Tukey’s test (B, C, D, and F) and two-way ANOVA with Tukey's test (H–J). *p* < 0.05 was considered significant.

The ability to stimulate a humoral immune response is an important indicator of adjuvant efficacy. The humoral immune response largely depends on B cell activity in the germinal center (GC) of lymph nodes. To evaluate the effects of Nano-ZD and Nano-ZDM, mice were immunized with adjuvant/OVA on day 0 and boosted on day 14. Serum was collected on day 21 for OVA-specific IgG detection, and DLNs were analyzed by flow cytometry (Figure 4A). Compared with Lipo-MPLA and Alum, both Nano-ZD and Nano-ZDM induced higher OVA-specific IgG titers, with Nano-ZDM showing the strongest effect (Figure 4B). Nano-ZD and Nano-ZDM significantly accelerated lymph node expansion according to the cell counting of DLNs (Figure 4C). They also increased the proportion of total B cells (B220^+^) and enriched humoral immunocompetent B cells (B220^+^GL7^+^) (Figures 4D–4F). Notably, Nano-ZDM further highly expanded CXCR5^+^ PD1^+^ follicular CD4^+^ T cells (Figure S12). These results indicated that Nano-ZD, as an immune-metabolic adjuvant, promoted the GC B-cell response, whereas Nano-ZDM further increased the efficacy via the sensitizing effects of Nano-ZD. We then comprehensively evaluated the effects of Nano-ZD adjuvants on humoral immune responses by loading different antigens, including hemagglutinin antigen HA1 from influenza virus, spike S1 from novel coronavirus, and HPV 16 from human papillomavirus (Figures 4G–4J). Measurement of antigen-specific IgG titers showed that Nano-ZD and Nano-ZDM achieved comparable or superior responses to Lipo-MPLA and Alum from weeks 3–5 post-immunization, demonstrating their strong capacity to activate humoral immunity.

### Nano-granulated zoledronate effectively activates cellular immune responses for cancer immunotherapy

**Figure 5:**
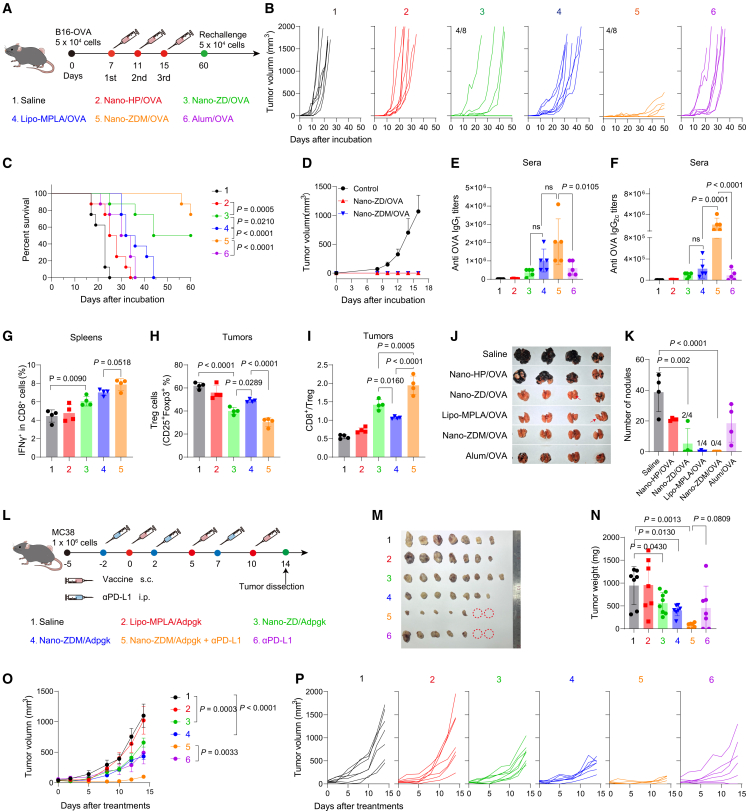
Nano-granulated zoledronate effectively activates cellular immune responses for cancer immunotherapy (A) Immunotherapy scheme for B16-OVA tumors. (B and C) Individual tumor growth and survival curves of B16-OVA-bearing mice (*n* = 8). (D) Tumor growth after rechallenging on day 60 (*n* = 8 of control group, *n* = 4 of Nano-ZD and Nano-ZDM). (E and F) Serum anti-OVA IgG1 and IgG2c titers (*n* = 5). (G) IFNγ^+^ CD8^+^ T cell percentage in spleens (*n* = 4). (H and I) Treg percentage and CD8^+^/Treg ratios in tumors (*n* = 4). (J and K) Photographs of lung metastasis of B16-OVA melanoma and numbers of metastasis nodules (*n* = 4). (L) Immunotherapy scheme for MC-38 tumors (*n* = 7–8). (M and N) Weight and photographs of excised tumors. (O and P) Tumor growth of MC-38-bearing mice. *n* indicates number of mice. Data are shown as mean ± SEM. for (O) and mean ± SD for other panels. Statistical significance was calculated by log-rank test (C), one-way ANOVA with Tukey’s test (E–I and K), unpaired two-tailed Welch’s *t* test (N), and two-way ANOVA with Tukey's test (O). *p* < 0.05 was considered significant.

We next evaluated the efficacy of Nano-ZD and Nano-ZDM as immune-metabolic adjuvants for cancer vaccines. All adjuvants used thereafter were mixed with the model antigen OVA or tumor neoantigen to form a complete vaccine system. First, B16-OVA tumor cells were subcutaneously inoculated, and mice were immunized three times with different adjuvant/OVA formulations (Figure 5A). By monitoring tumor growth and overall survival, Nano-ZD and Nano-ZDM showed more effective anti-tumor effects than Lipo-MPLA and Alum, with Nano-ZDM being the most effective. Four of 8 mice in each of these two groups achieved complete tumor inhibition (Figures 5B, 5C, and S13A). These tumor-free mice were rechallenged with B16-OVA on the contralateral side on day 60. Unlike the rapid tumor growth in unvaccinated controls, mice immunized with Nano-ZD or Nano-ZDM maintained potent antitumor effects and none experienced tumor recurrence (Figures 5D and S13C).

To elucidate the detailed regulatory process of Nano-ZD and Nano-ZDM, peripheral blood, spleens, and tumor samples were collected and analyzed after immunization. Subtype analysis of OVA-specific IgG antibodies revealed that Nano-ZD increased serum levels of IgG1 and IgG2c, with effects comparable to Lipo-MPLA and Alum. Nano-ZDM showed the strongest capacity for IgG induction among all groups (Figures 5E and 5F). These results revealed the dual stimulatory effects of Nano-ZD and Nano-ZDM on helper T cells. Analysis of T lymphocyte subsets via flow cytometry indicated that both Nano-ZD and Nano-ZDM increased the proportion of CD3^+^ CD8^+^ T cells in the peripheral blood and facilitated the production of CD8^+^ IFN-γ^+^ cytotoxic T cells in the spleen, with Nano-ZDM showing the strongest efficacy (Figures 5G, S13D, S14A, and S14B). Similarly, these two adjuvants reduced intratumoral Treg proportions and increased the CD8^+^/Treg ratio (Figures 5H, 5I, S13H, and S14C–S14E). These findings suggested that Nano-ZD and Nano-ZDM effectively activate the cellular immune response for cancer immunotherapy by alleviating the immunosuppressive tumor microenvironment while enhancing systemic immune responses in the blood and spleens. Nano-ZD and Nano-ZDM also increased the proportion of CD8^+^ and CD4^+^ T cells with effector memory phenotypes, supporting their long-term antitumor efficacy (Figures S13E, S13F, and S15). Full-spectrum flow cytometry showed that Nano-ZD and Nano-ZDM remodeled the tumor immune microenvironment by enhancing functional T cells and modulating myeloid populations, with Nano-ZDM more effectively reducing Tregs and repolarizing macrophages (Figures S16). Transcriptomic analysis of tumor tissues also indicated that Nano-ZD triggered a pro-inflammatory transcriptomic profile, while Nano-ZDM mediated a much stronger and broader immunomodulatory response (Figures S17 and S18).

In addition to immunotherapy efficacy, the safety of Nano-ZD and Nano-ZDM was evaluated (Figures S19, S13B, and S13G). Mice treated with the adjuvants did not exhibit systemic toxicity, including obvious body weight loss and abnormal blood biochemistry. Hematoxylin and eosin (H&E) staining confirmed good organ biocompatibility. Routine blood tests showed that Nano-ZDM slightly increased white blood cell, lymphocyte, and granulocyte counts, likely reflecting systemic immune activation.

An enhanced systemic immune response is beneficial for suppressing tumor metastasis. We further evaluated the effects of Nano-ZD and Nano-ZDM on the pulmonary metastasis of melanoma by intravenously injecting B16-OVA cells into vaccinated mice. Nano-ZD effectively prevented the pulmonary metastasis of melanoma, with 2 of 4 mice being free of nodules. Notably, Nano-ZDM almost completely blocked lung tumor lesions (Figures 5J and 5K).

Additionally, we evaluated the synergistic effect of the immune-metabolic adjuvants with an immune checkpoint blocker, αPD-L1, in the MC38 colorectal carcinoma model (Figure 5L). After simultaneously treating the mice with the MC38-specific neoantigen peptide Adpgk and Nano-ZD or Nano-ZDM, tumor growth was effectively suppressed. Notably, the combination of αPD-L1 and Nano-ZDM further improved the therapeutic efficacy, with the highest tumor inhibition rate of 88.7% on day 14 compared with saline group (Figures 5M–5P). Furthermore, we evaluated the proportions of OVA-specific tetramer^+^ CD8^+^ T cells, IFNγ^+^ CD8^+^ T cells, and Granzyme B^+^ CD8^+^ T cells in B16-OVA tumor-bearing mice following combination treatment with Nano-ZDM/OVA and αPD-L1. Nano-ZDM plus αPD-L1 enhanced antigen-specific cytotoxic T cell responses both in the spleen and in the tumor tissues (Figures S20). This demonstrated good synergy, paving the way for the application of Nano-ZD and Nano-ZDM in the treatment of different types of tumors.

### Nano-granulated zoledronate sensitizes and amplifies innate immunity by activating pyrin inflammasome and triggering mtROS-dependent MAVS oligomerization

Although the potential of the MVA metabolic pathway as an adjuvant target has been confirmed by studies on statins,^21^ given that MVA metabolism is a multi-branched pathway, the molecular mechanism of immune regulation based on other targets in the pathway is still not fully understood. Additionally, the mechanism by which the inhibition of MVA metabolism sensitizes and amplifies innate immune responses also remains unclear.

**Figure 6:**
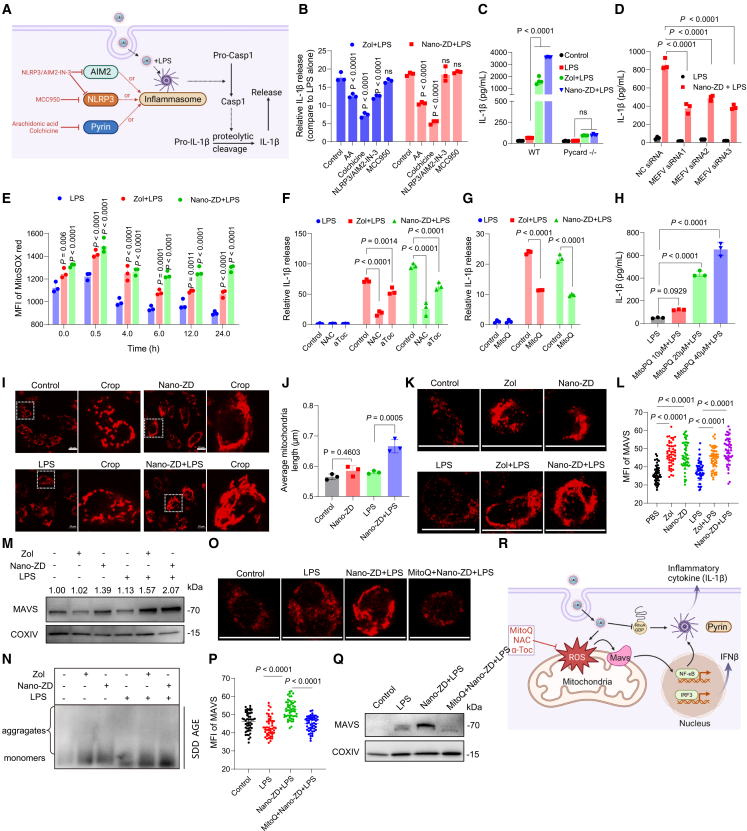
Nano-granulated zoledronate sensitizes and amplifies innate immunity by activating pyrin inflammasome and triggering mtROS-dependent MAVS oligomerization (A) Schematic of inflammasome activation analysis. (B) Relative IL-1β release after inhibitor treatment (*n* = 3). (C) IL-1β levels in Pycard^−/−^ BMDCs (*n* = 4). (D) IL-1β levels in BMDCs after MEFV siRNA treatment (*n* = 3). (E) mtROS production in BMDCs (*n* = 3). (F–H) IL-1β levels after antioxidant supplementation (NAC and α-Toc in F and MitoQ in G) or treatment with MitoPQ (H) (*n* = 3). (I and J) Confocal images (I) and ImageJ quantification (J) of mitochondrial morphology in MitoTracker Deep Red-stained BMDCs. Scale bars, 10 μm. (K and L) Immunofluorescence images (K) and MFI (L) of MAVS. Scale bars, 10 μm. Fifty cells per sample were measured. (M) Western blot analysis of mitochondrial MAVS expression. COXIV as loading control. (N) SDD-AGE analysis of MAVS aggregation. (O–Q) MAVS detection after MitoQ supplementation. (O and P) Immunofluorescence images and MFI quantified by ImageJ. Scale bars, 10 μm. (Q) Western blot analysis of mitochondrial MAVS expression. (R) Schematic of the mechanism of Nano-ZD in sensitizing and amplifying innate immunity. Created in https://BioRender.com. *n* indicates biologically independent samples. Data are shown as mean ± SD. Statistical significance was calculated by two-way ANOVA with Tukey’s test (B–G) and one-way ANOVA with Tukey's test (H, J, L, and P). *p* < 0.05 was considered significant.

Inflammasome activation is a key step to innate immune response, and there are several types of inflammasomes.^28^^,^^29^ Inflammasome activation cleaves pro-caspase-1 or pro-caspase-11, leading to IL-1β maturation and release. We previously showed that Zol and Nano-ZD increased IL-1β production and potentiated LPS-induced IL-1β secretion (Figures 1E and 2N). These findings revealed that the inflammasome pathway located upstream was involved in the sensitizing and amplifying effects of Zol and Nano-ZD. To identify the key inflammasome, we compared the influences of distinct inhibitors that block the assembly or activity of inflammasomes on the IL-1β production induced by LPS plus Zol or Nano-ZD (Figure 6A). Colchicine and arachidonic acid (AA), two inhibitors of pyrin inflammasome by activating RhoA or PKNs/ROCKs,^30^ significantly reduced educed IL-1β secretion (Figure 6B). NLRP3/AIM2-IN-3, as co-inhibitors of the NLRP3 and AIM2 inflammasomes, had a weak inhibitory effect on IL-1β production. However, MCC950, a specific inhibitor of the NLRP3 inflammasome, had no effect on IL-1β secretion. These data highlight pyrin inflammasome as the central driver. To further confirm this, we cultured BMDCs from Pycard^−/−^ and NLRP3^−/−^ mice to investigate their responses to the treatment with Zol or Nano-ZD (Figures 6C and S21A–S21C). The knockout of Pycard significantly inhibited IL-1β production, further demonstrating the involvement of the inflammasome pathway. Nevertheless, IL-1β secretion induced by LPS plus Zol or Nano-ZD was not suppressed in NLRP3^−/−^ BMDCs. In contrast, when BMDCs or BMDMs were pretreated with MEFV-small interfering RNA (siRNA) to reduce the protein expression of pyrin, IL-1β production caused by LPS plus Nano-ZD was markedly inhibited (Figures 6D, S21D, and S21E). In summary, these results clearly suggested that the sensitization and amplification of innate immune responses by Zol and Nano-ZD was mediated by the pyrin inflammasome.

Activation of the pyrin inflammasome is closely related to RhoA GTPase dysfunction.^30^ Some pathogens reportedly suppress host immune responses by modulating RhoA GTPases and downstream pyrin activity.^29^ Activation of RhoA GTPase is tightly regulated by MVA metabolism. Newly synthesized RhoA GTPase requires GGPP, generated by the MVA pathway, for membrane anchoring and function. Therefore, inhibition of MVA metabolism can cause dysfunction of RhoA GTPase. Western blot analysis of RhoA GTPase in the membrane and cytosol showed that Nano-ZD obviously reduced membrane anchoring of RhoA GTPase, which suggested GTPase inactivation (Figure S21F). These findings demonstrated that Nano-ZD activated the pyrin inflammasome through the MVA metabolism-RhoA GTPase pathway.

Compelling evidence has shown that metabolic changes and redox pathways are tightly interwoven and interdependent to ultimately regulate immune cell function.^5^^,^^31^ Our previous work demonstrated that bisphosphonates disrupt redox homeostasis, leading to the accumulation of mtROS. This accumulation triggers ferroptosis in tumor cells by inhibiting MVA metabolism.^25^ Here, we first investigated the impacts of Zol and Nano-ZD on redox homeostasis in BMDCs. By labeling mtROS with MitoSox red, it was revealed that Zol and Nano-ZD facilitated mtROS production based on LPS treatment, like their ability to increase the secretion of proinflammatory cytokines (Figure 6E). We explored whether increased mtROS affected the innate immune response by adding different antioxidants. Both N-acetyl-L-cysteine (NAC) and α-tocopherol (α-Toc) significantly inhibited IL-1β production caused by LPS plus Zol or Nano-ZD (Figure 6F). MitoQ, a mitochondria-targeting antioxidant, markedly reduced IL-1β secretion in BMDCs (Figure 6G). In contrast, when mtROS were specifically increased by addition of the mitochondria-targeting superoxide generator MitoPQ, large amounts of IL-1β were produced in a concentration-dependent manner (Figure 6H). These results indicated that mtROS, as a crucial regulator, is involved in the innate immune response mediated by the pyrin inflammasome.

Nevertheless, how does mtROS regulate pyrin-dependent immunity? Given that mitochondria are the major source of ROS and sensitize IL-1β secretion, we focused on studying the effect of mitochondria variations on immunity. MitoTracker staining showed that Zol and Nano-ZD altered mitochondrial morphology. Many ellipsoidal mitochondria in the control group were fused with each other and shaped as bars or rods after Zol and Nano-ZD treatments (Figures 6I and S21G). This morphological change was significantly amplified in the LPS plus Zol or Nano-ZD groups, with the average mitochondrial length increasing by nearly 20% compared with the control group (Figure 6J).

A direct consequence of mitochondrial fusion is the rearrangement of mitochondrial membrane proteins. MAVS, a protein localized to the mitochondrial outer membrane, has been reported to activate multiple downstream immune-related signaling pathways by regulating its own oligomerization in response to mitochondrial fusion.^32^ We next investigated the impacts of Zol and Nano-ZD on the expression of MAVS in BMDCs. Confocal microscopy revealed that Zol and Nano-ZD treatments significantly upregulated MAVS expression, which was further confirmed by western blot analysis of mitochondrial proteins (Figures 6K–6M and S22A). Similar to the changes in mitochondrial morphology, MAVS morphology in mitochondria also exhibited fusion and aggregation features in the Zol- and Nano-ZD-treated groups. More importantly, the imaging based on semi-denaturing detergent agarose gel electrophoresis (SDD-AGE) showed that more MAVS proteins were retarded in gel due to the oligomerization after Zol and Nano-ZD treatments (Figures 6N and S22B–S22D). These results indicated that Zol and Nano-ZD promoted MAVS oligomerization by inducing mitochondrial fusion, which established a prerequisite for the activation of downstream immune signals.

Multiple immune signaling pathways, including those involving NF-κB and IRFs, are closely related to MAVS activation.^31^^,^^33^ Using both western blot analysis and an NF-κB reporter assay, we demonstrated that Zol and Nano-ZD potentiated NF-κB pathway activation (Figures S23A–S23B). It has been reported that pyrin encoded by the MEFV gene and pro-IL-1β encoded by the IL-1B gene are under the regulation of NF-κB signaling.^28^^,^^29^ Correspondingly, compared with LPS alone, LPS plus Zol or Nano-ZD increased pyrin and pro-IL-1β expression by 20%–40% by western blot (Figure S23C). These findings suggested that more pyrin inflammasomes could be activated as a result of pyrin upregulation by Zol and Nano-ZD. We also investigated the impact of MAVS oligomerization on IRF signaling pathway. Poly I:C was transfected into the cytosol to assess IRF signaling. Zol and Nano-ZD promoted MAVS oligomerization, enhanced IRF activation, and increased IFNβ production (Figure S23D), indicating that they sensitize IRF signaling in addition to activating the pyrin inflammasome. Finally, to determine whether the MAVS oligomerization caused by Zol and Nano-ZD depends on mtROS, we investigated the variation in MAVS expression by adding MitoQ. Both CLSM and western blot imaging demonstrated that MitoQ markedly reduced the expression and fusion of MAVS induced by LPS plus Nano-ZD (Figures 6O–6Q and S24).

Taken together, these results demonstrate that both free zoledronate and Nano-ZD effectively sensitize and amplify innate immune responses by activating the pyrin inflammasome through the MVA metabolism-RhoA GTPase pathway and triggering mtROS-dependent MAVS oligomerization to promote immune-related gene expression (Figure 6R).

### Nano-granulated zoledronate induces mtROS production through the mevalonate-CoQ-OXPHOS/pyrimidine metabolism axis

**Figure 7:**
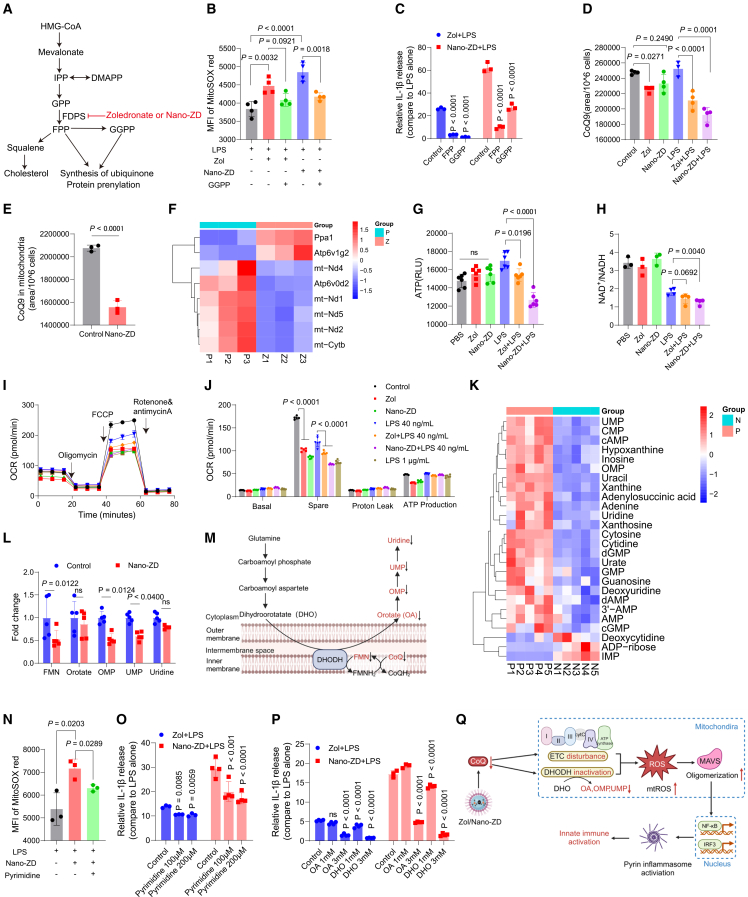
Nano-granulated zoledronate induces mtROS production through the mevalonate-CoQ-OXPHOS/pyrimidine metabolism axis (A) Schematic of the effects of Zol/Nano-ZD on the mevalonate pathway. (B) mtROS production after GGPP supplementation (*n* = 4). (C) Relative IL-1β release after GGPP and FPP supplementation (*n* = 3). (D) CoQ9 levels in whole BMDCs (*n* = 3–4). (E) CoQ9 levels in the mitochondria of BMDCs (*n* = 3). (F) Heatmap of DEGs related to mitochondrial oxidative phosphorylation signaling pathway (KEGG). (P, untreated; Z, zoledronate treated; *n* = 3). (G–J) BMDCs were pretreated with zoledronate or Nano-ZD for 24 h and stimulated with LPS for 12 h. ATP production (G, *n* = 6). NAD^+^/NADH ratio (H, *n* = 3–4). Seahorse analysis of OXPHOS-related OCR parameters (I and J, *n* = 5–6). (K) Heatmap of differential metabolites involved in pyrimidine and purine metabolism (P, untreated; Z, Nano-ZD treated; *n* = 5). (L) Fold change of metabolites in the *de novo* pyrimidine biosynthesis. (M) Schematic of *de novo* pyrimidine biosynthesis. (N) mtROS production after pyrimidine supplementation (*n* = 3). (O) Relative IL-1β release after pyrimidine supplementation (*n* = 3–4). (P) Relative IL-1β release after OA (orotate) or DHO (dihydroorotate) (*n* = 3) supplementation. (Q) Schematic of the mechanism by which Zol/Nano-ZD exert immune effects. Created in https://BioRender.com. *n* indicates biologically independent samples. Data are shown as mean ± SD. Statistical significance was calculated through one-way ANOVA with Tukey’s test (B, D, and G), unpaired two-tailed Student’s *t* test (E, H, and N), and two-way ANOVA with Tukey's test (C, J, L, O, and P). *p* < 0.05 was considered significant.

A more fundamental mechanistic question was how Zol and Nano-ZD induce mtROS production. According to the MVA metabolic pathway, FDPS is located at a key node in this multi-branched pathway (Figure 7A). FPP can generate cholesterol, and can also be catalyzed by GGPS to produce GGPP for small GTPase modification. Additionally, FPP and GGPP can serve as substrates for CoQ synthesis. To determine which branch contributes to mtROS production, we examined mtROS and IL-1β levels during LPS plus Zol or Nano-ZD treatment with supplementation of metabolites from different branches. GGPP supplementation significantly suppressed mtROS elevation induced by LPS plus Zol or Nano-ZD (Figure 7B). Both GGPP and FPP attenuated the proinflammatory effect of LPS plus Zol or Nano-ZD by reducing IL-1β and TNF-α production (Figures 7C and S25A), whereas cholesterol had no impact on the immune response (Figure S25B). These results suggest that the isoprenylation of proteins or CoQ based on GGPP plays a crucial role in mtROS-dependent immune regulation. We also examined whether these mechanistic findings apply to Nano-ZDM. Supplementation with GGPP, FPP, MitoQ, AA, and colchicine attenuated Nano-ZDM-induced IL-1β or TNF-α upregulation (Figures S25J–S25M). Nano-ZDM also increased mtROS and MAVS expression (Figures S25N–S25P), indicating that the principal mechanistic framework applies to Nano-ZDM.

Next, we compared gene expression before and after zoledronate treatment using transcriptome sequencing. Kyoto Encyclopedia of Genes and Genomes (KEGG) and GO analyses showed that zoledronate downregulated genes associated with mitochondrial OXPHOS and NADH dehydrogenase (ubiquinone) activity (Figure S26A–S26C). Given that NADH dehydrogenase in OXPHOS uses CoQ as the electron transporter, we speculated that the decreased CoQ levels might underlie OXPHOS dysfunction. Liquid chromatography-tandem mass spectrometry analysis showed that Zol and Nano-ZD decreased CoQ9 levels in BMDCs and LPS further reduced CoQ9 (Figures 7D and S26D). We also determined mitochondrial CoQ9 levels and confirmed their reduction after Nano-ZD treatment (Figure 7E). Supplementation with idebenone (a CoQ analog) alleviated the proinflammatory effects and mtROS elevation induced by Nano-ZD (Figures S25C and S25D). Supplementation with GGPP plus FPP partially restored CoQ9 levels (Figure S25E). These results indicate that CoQ in the MVA pathway is a key mediator of the immunoregulatory effects of Zol and Nano-ZD.

The transcriptomic analysis showed OXPHOS dysfunction after zoledronate treatment (Figure S26). According to the comparison of DEGs shown in the heatmap, mt-nd1, mt-nd2, and mt-nd5, which are involved in complex I assembly in the electron transport chain (ETC), and mt-Cytb, which is part of MRC complex III, were significantly downregulated by zoledronate (Figure 7F). This finding reflected the degradation of electron transport in the mitochondria. Correspondingly, ATP production was markedly reduced by LPS plus Zol or Nano-ZD (Figure 7G). NAD^+^/NADH ratios were further decreased compared with LPS alone (Figure 7H), indicating complex I impairment. Seahorse analysis showed that Zol and Nano-ZD treatments led to a substantial reduction in spare respiratory capacity (SRC), and the addition of LPS (40 ng/mL) further exacerbated this reduction, resulting in a decrease in the SRC similar to that observed after treatment with high concentrations of LPS (1 μg/mL, Figures 7I and 7J). Supplementation with GGPP and FPP partially rescued SRC loss induced by Nano-ZD alone. In contrast, idebenone did not significantly restore the SRC loss caused by Nano-ZD alone, although, compared with the Nano-ZD plus LPS group, idebenone led to a partial recovery of SRC (Figures S25F–S25I). We propose that although idebenone is a CoQ10 analog with antioxidant properties, it cannot fully substitute for the native functions of the predominant murine CoQ isoprenolog, CoQ9. Therefore, the complete recovery of mitochondrial respiratory capacity appears to depend specifically on restored endogenous CoQ9 synthesis. These results revealed that Zol and Nano-ZD impaired OXPHOS by decreasing CoQ levels, laying the foundation for mtROS accumulation.

We next investigated whether CoQ reduction caused by Nano-ZD affected other mitochondrial processes. Untargeted metabolomics revealed that many pathways, including pyrimidine metabolism and purine metabolism, were downregulated after Nano-ZD treatment (Figure S27). Multiple metabolites directly involved in *de novo* pyrimidine synthesis, including uridine, uridine 5′-monophosphate (UMP), orotidine 5′-monophosphate (OMP), orotate (OA), and flavin mononucleotide (FMN), were significantly decreased after Nano-ZD treatment (Figures 7K–7L). Pyrimidine biosynthesis is initiated from glutamine to produce aspartate, which then results in the synthesis of dihydroorotate (DHO) outside the mitochondria.^34^ The latter is oxidized by dihydroorotate dehydrogenase (DHODH), which is located in the inner membrane of mitochondria, to generate OA, which is then catalyzed by phosphoribosyl transferase to generate OMP and finally obtain UMP and uridine (Figure 7M).^35^^,^^36^ Since DHODH requires CoQ and FMN to transport electrons, decreased CoQ levels caused by Nano-ZD led to DHODH inactivation and suppressed pyrimidine metabolism. Correspondingly, increased production of mtROS and IL-1β caused by LPS plus Zol or Nano-ZD was attenuated when DHO, OA, or pyrimidine were supplemented to restore pyrimidine metabolism (Figures 7N–7P).

These results delineated a complete mechanism by which Zol and Nano-ZD exert immune effects. Both Zol and Nano-ZD reduce the synthesis of CoQ by inhibiting MVA metabolism. Reduced CoQ impaired the ETC and disrupted OXPHOS, while suppressing pyrimidine metabolism. These mitochondrial changes led to mtROS accumulation, which then caused MAVS oligomerization and ultimately sensitized and amplified pyrin-dependent immune responses (Figure 7Q).

## Discussion

Metabolic reprogramming provides a strategy for functional remodeling of innate immune cells that differs from PRR activation.^3^ Targeting specific metabolic pathways can function similarly to classical vaccine adjuvants to enhance innate immunity and antigen presentation.^14^ However, because most metabolic pathways are ubiquitous, achieving targeted delivery and specific regulation of metabolic adjuvants to innate immune cells *in vivo* remains challenging.^37^ To address this, we designed Nano-ZD, in which zoledronate coordinates with calcium ions to form nanoscale granules with lipid assistance. Subcutaneously injected nanoparticles preferentially enter lymphatic vessels and traffic to lymph nodes, particularly when sized between 10 and 100 nm.^38^ The average diameter of Nano-ZD was approximately 50 nm, facilitating transport to lymph nodes after subcutaneous injection. Imaging analyses of living animals and tissue sections demonstrated the delivery of Nano-ZD to lymph nodes. This nanotechnology-based delivery strategy is critical for zoledronate. As an inhibitor of the MVA pathway, zoledronate has been approved for the clinical treatment of osteoporosis and bone tumors. The bisphosphonate groups in zoledronate have a strong affinity for calcium ions, resulting in strong bone tropism after entering the blood and potentially causing adverse reactions such as hypocalcemia.^22^^,^^39^^,^^40^ In our study, free zoledronate injected subcutaneously was rapidly absorbed into the bloodstream and distributed to the bone, thus losing its ability to enhance innate immune responses *in vivo*. Interestingly, the high affinity between zoledronate and calcium ions led us to design and construct Nano-ZD. Nano-ZD was taken up mainly by innate immune cells, including macrophages and DCs in DLNs. Nano-ZD remained intact in a neutral environment but disassembled in a weakly acidic environment at pH 5.0, allowing zoledronate release in acidic phagolysosomes to exert immunoregulatory effects. Unlike free zoledronate, Nano-ZD exhibited adjuvant efficacy both *in vitro* and *in vivo*. This highlights the importance of changing the *in vivo* distribution of metabolic modulators through nanotechnology to achieve the targeted regulation of innate immune responses. The strategy developed in this study also provides a general solution based on nano-granulation for other metabolic modulators with potential adjuvant efficacy.

The MVA metabolic pathway has been validated as a target for vaccine adjuvants through studies of hydrophobic statins.^21^ Statins inhibit geranylgeranylation of small GTPases, resulting in arrested endosomal maturation, prolonged antigen retention, enhanced antigen presentation, and increased T cell activation. Nevertheless, given that MVA metabolism is multi-branched, it remains unclear whether inhibition of this pathway could modulate innate immunity through alternative branches. Furthermore, studies involving statins as well as our research on zoledronate have demonstrated that the inhibition of MVA metabolism sensitizes and amplifies innate immune responses triggered by PAMPs, whereas the precise molecular mechanisms remain to be elucidated.^41^^,^^42^^,^^43^ Here, we demonstrated that both free zoledronate and Nano-ZD not only inhibited small GTPase isoprenylation but also reduced CoQ biosynthesis. CoQ reduction impaired mitochondrial complex I and complex III function, disrupted electron transport equilibrium, diminished SRC, and disrupted OXPHOS. CoQ deficiency also decreased *de novo* pyrimidine synthesis by inactivating DHODH in mitochondria. Both OXPHOS and pyrimidine metabolism are involved in maintaining redox homeostasis in mitochondria.^44^^,^^45^ Dysregulation of these pathways led to mtROS accumulation, triggering mitochondrial fusion and MAVS oligomerization. As a hub of mitochondrial immune regulation, MAVS activated multiple downstream pathways, including NF-κB and IRF, increasing the expression of the inflammasome component pyrin and the proinflammatory factor pro-IL-1β. Because these pathways are also activated by classical PAMPs such as LPS, the superposition of these signals significantly enhanced innate immune responses, thus explaining the sensitization mechanism of free zoledronate and Nano-ZD. This mechanism depends on the MVA-CoQ-OXPHOS/pyrimidine metabolism axis. Supplementation of metabolites in this axis diminished the amplification effect of Nano-ZD. These findings suggest that the MVA-CoQ-OXPHOS/pyrimidine metabolism axis may serve as a target for immune-metabolic vaccine adjuvants.

### Limitations of the study

Several limitations warrant consideration. Although we delineate an MVA-CoQ-OXPHOS/pyrimidine metabolism axis at the cellular level, *in vivo* validation of this mechanistic cascade remains limited. Given that the MVA pathway is highly branched and broadly regulates cellular metabolism, multiple downstream pathways beyond FDPS inhibition may contribute to immune regulation. The relative contributions of these distinct branches to innate immune sensitization require further quantitative dissection in animal models. In addition, although no overt systemic toxicity was observed in our study, the long-term consequences of sustained immune activation and potential risks associated with excessive inflammasome activation require further investigation.

## Resource availability

### Lead contact

Requests for further information, resources, and reagents should be directed to and will be fulfilled by the lead contact, Bing He (hebingmumu@bjmu.edu.cn).

### Materials availability

All unique/stable reagents generated in this study are available from the lead contact with a completed materials transfer agreement.

### Data and code availability

• RNA sequencing raw reads have been deposited in NCBI’s Sequence Read Archive (SRA) as BioProject : PRJNA1185916, PRJNA1186379, andPRJNA1428487. The raw metabolomics data have been deposited in the MetaboLights as MetaboLights: MTBLS11696.
• This paper does not report original code.
• Any additional information required to reanalyze the data reported in this work paper is available from the lead contact upon request.

## Acknowledgments

This work was supported by 10.13039/501100012166National Key Research and Development Program of China (#2023YFC2605004 and 2022YFA1206104 to B.H.), 10.13039/100007219Beijing Natural Science Foundation (#Z230017 to B.H.), key project at central government level: The ability establishment of sustainable use for valuable Chinese medicine resources (#2060302 to Q.Z.), The 10.13039/501100013090Science and Technology Innovation Project of the Guangdong Provincial Medical Products Administration (#2024ZDZ02 to B.H.), 10.13039/501100007129Natural Science Foundation of Shandong Province (#ZR2023ZD25 to B.H.), and 10.13039/100014718National Natural Science Foundation of China (#81971718, 82073786, and 81872809 to X.W.; #U20A20412 and 81821004 to Q.Z.). We thank the 10.13039/100031918State Key Laboratory of Natural and Biomimetic Drugs, Peking University Medical and Health Analysis Center, for confocal and flow cytometry services.

## Author contributions

M.C. and B.H. were responsible for all phases of this project. X.J., Z.Y., Yueheng Wang, Q.W., and M.L. assisted with cellular and *in vivo* antitumor experiments. Z.Y. helped with western blot analysis. J.Z. assisted with siRNA knockdown assay. S.F. helped with HPLC analysis. Yuan Wang assisted with LC-MS/MS analysis. H.Z., W.D., and X.W. provided conceptual advice and supervision. M.C. and B.H. wrote the manuscript. B.H. and Q.Z. revised the final draft. All authors discussed the results and commented on the manuscript.

## Declaration of interests

The authors declare no competing financial interests.

## STAR★Methods

### Key resources table

**Table:** 

REAGENT or RESOURCE	SOURCE	IDENTIFIER
**Antibodies**		

APC anti-mouse CD11c	Biolegend	Cat # 117309; RRID: AB_313778
PE anti-mouse CD86	Biolegend	Cat # 105007; RRID: AB_313150
PE/Cyanine7 anti-mouse CD80	Biolegend	Cat # 104733; RRID: AB_2563112
PerCP/Cyanine5.5 anti-mouse I-A/I-E	Biolegend	Cat # 107625; RRID: AB_2191072
FITC anti-mouse F4/80	Biolegend	Cat # 123107; RRID: AB_893500
PE anti-mouse H-2K^d^	Biolegend	Cat # 116608; RRID: AB_313743
APC anti-mouse CD3	Biolegend	Cat # 100235; RRID: AB_2561455
Pacific Blue™ anti-mouse CD45	Biolegend	Cat # 103125; RRID: AB_493536
Alexa Fluor® 488 anti-mouse CD11c	Biolegend	Cat # 117313; RRID: AB_492849
PE anti-mouse CD86	Biolegend	Cat # 105007; RRID: AB_313150
PE/Cyanine7 anti-mouse CD80	Biolegend	Cat # 104733; RRID: AB_2563112
PE anti-mouse CD8a	Biolegend	Cat # 100707; RRID: AB_312746
PE/Cyanine7 anti-mouse CD4	Biolegend	Cat # 100421; RRID: AB_312706
PE/Cyanine7 anti-mouse CD11c	Biolegend	Cat # 117317; RRID: AB_493569
PE/Dazzle™ 594 anti-mouse/human CD11b	Biolegend	Cat # 101255; RRID: AB_2563647
Alexa Fluor® 488 anti-mouse/human CD45R/B220	Biolegend	Cat # 103228; RRID: AB_492874
PE anti-mouse F4/80	Biolegend	Cat # 123109; RRID: AB_893498
FITC anti-mouse CD3	Biolegend	Cat # 100203; RRID: AB_312660
PE/Cyanine5 anti-mouse/human CD45R/B220	Biolegend	Cat # 103209; RRID: AB_312994
PE anti-mouse/human GL7 Antigen	Biolegend	Cat # 144607; RRID: AB_2562925
APC anti-mouse CD8a	Biolegend	Cat # 100711; RRID: AB_312750
PE anti-mouse CD279 (PD-1)	Biolegend	Cat # 135205; RRID: AB_1877232
Brilliant Violet 421™ anti-mouse CD185 (CXCR5)	Biolegend	Cat # 145511; RRID: AB_2562127
Brilliant Violet 605™ anti-mouse IFN-γ	Biolegend	Cat # 505839; RRID: AB_2561438
PE anti-mouse FOXP3	Biolegend	Cat # 126403; RRID: AB_1089118
PE/Cyanine5 anti-mouse/human CD44	Biolegend	Cat # 103009; RRID: AB_312960
PE anti-mouse CD62L	Biolegend	Cat # 104407; RRID: AB_313094
CD25 Monoclonal Antibody (PC61.5), PerCP-Cyanine5.5	Invitrogen	Cat # 45-0251-80; RRID: AB_914323
Goat Anti-Mouse IgG(H + L)-HRP	Southernbiotech	Cat # 1036-05; RRID: AB_2794348
Goat Anti-Mouse IgG1-HRP	Southernbiotech	Cat # 1071-05; RRID: AB_2794426
Goat Anti-Mouse IgG2c-HRP	Southernbiotech	Cat # 1078-05; RRID: AB_2794462
rabbit anti-IL-1β	Abcam	Cat # ab234437; RRID: AB_2936228
rabbit anti-Pyrin	Abcam	Cat # ab195975; RRID: AB_3674164
rabbit anti-RhoA	Abcam	Cat # ab187027; RRID: AB_2827434
rabbit anti-Sodium Potassium ATPase	Abcam	Cat # ab76020; RRID: AB_1310695
rabbit anti-MAVS	Abcam	Cat # ab314444; RRID: AB_3741558
mouse anti-COXIV	CST	Cat # 11967T; RRID: AB_2797784
Dylight 649 Goat anti-Rabbit secondary antibody	Abbkine	Cat # A23620; RRID: AB_3661755
PE/Cyanine5 anti-human/mouse Granzyme B	Biolegend	Cat # 372225; RRID: AB_2910421;
Brilliant Violet 570™ anti-mouse Ly-6G	Biolegend	Cat # 127629; RRID: AB_10899738
Alexa Fluor® 488 anti-mouse Ly-6C	Biolegend	Cat # 128021; RRID: AB_10640820
APC anti-mouse CD206 (MMR)	Biolegend	Cat # 141707; RRID: AB_10896057
T-Select H-2Kb OVA Tetramer-SIINFEKL-PE	MBL	Cat # TS-5001-1C; RRID: AB_3090188
InVivoMab anti-mouse PD-L1 (B7-H1)	Bioxcell	Cat # BE0101; RRID: AB_10949073

**Chemicals, peptides, and recombinant proteins**		

Hemagglutinin/HA Protein	SinoBiological	Cat # 40938-V08H
Spike S1-His Recombinant Protein	SinoBiological	Cat # 40591-V08H
Human Papilloma Virus type 16 (HPV 16) L1 protein (VLP)	SinoBiological	Cat # HPV16
GM-CSF	PeproTech	Cat # 315-03-20
M-CSF	PeproTech	Cat # 315-02-10
AF647-Zol	BioVinc	Cat # BV501001
Ovalbumin (OVA)	Shanghai Yuanye	Cat #S12015
Adpgk (CGIPVHLELASMTNMELMSSIVHQQVFPT)	ChinaPeptides (QYAOBIO)	N/A
Mitotracker deep red	Invitrogen	Cat # A66440
MitoSox red	Invitrogen	Cat #M36008
Geranylgeranyl Pyrophosphate ammonium salt (GGPP)	GlpBio	Cat # GC43747
Mitoquinone (MitoQ)	GlpBio	Cat # GC30416
α-Tocopherol phosphate disodium(α-Toc)	GlpBio	Cat # GC67618
Farnesyl pyrophosphate ammonium salt (FPP)	Macklin	Cat #F922191
Idebenone	Macklin	Cat #I844312
N-acetyl-L-cysteine (NAC)	Macklin	Cat # ST2524
MitoPQ	MCE	Cat # HY-130278
Arachidonic acid	MCE	Cat # HY-109590
Colchicine	MCE	Cat # HY-16569
NLRP3/AIM2-IN-3	MCE	Cat # HY-144226
MCC950	MCE	Cat # HY-12815A

**Critical commercial assays**		

Mouse IL-1β ELISA kit	Biolegend	Cat # 432604
Mouse TNF-α ELISA kit	Biolegend	Cat # 430904
mouse IL-6 ELISA kit	Biolegend	Cat # 431304
Mouse IL-12 p70 ELISA kit	Peprotech	Cat # 900-TM97
Mouse IFN-β ELISA kit	Solarbio	Cat # SEKM-0032
Mitochondria isolation kit	Beyotime	Cat #C3601

**Deposited data**		

RNA-sequencing of BMDCs and B16-OVA tumor tissues	This paper	NCBI Bioproject: PRJNA1185916, PRJNA1186379, PRJNA1428487
Untargeted metabolomics analysis	This paper	MetaboLights: MTBLS11696

**Experimental models: Cell lines**		

NF-κB Reporter RAW 264.7 Cells	InvivoGen	Cat # raw-sp
B16-OVA	Gift from Prof. Yiguang Wang	N/A
MC-38	Gift from Prof. Lei Miao	N/A

**Experimental models: Organisms/strains**		

C57BL/6J	PKUHSC Department of Laboratory Animal Science	N/A
C57BL/6JGpt-*Nlrp*^*3em7Cd4411*^/Gpt	GemPharmatech	strain number T010873
C57BL/6JGpt-*Pycardem1*^*Cd14740*^/Gpt	GemPharmatech	strain number T011758
OT-I mouse	Wukong Biotechnology	N/A

**Oligonucleotides**		

5′- GTAGAGAATAAAGCCAAGA-3′	RiboBio	N/A
5′- GCAGAAGATTTCATGTCCA-3′	RiboBio	N/A
5′- CCATTCTCGGAACAACAAA-3′	RiboBio	N/A

**Software and algorithms**		

BioRender	BioRender	https://www.biorender.com/
GraphPad Prism 8.0	GraphPad Software	https://www.graphpad.com/
FLowJo 10.8.1	BD Life Science	https://www.flowjo.com/
Seahorse Wave Pro	Agilent	https://www.agilent.com.cn/
CytExpert	Beckman	https://www.mybeckman.cn/
ImageJ	ImageJ	https://imagej.net/ij/
Living Image 4.3.1	PerkinElimer	https://www.perkinelmer.com.cn/

### Experimental model and study participant details

#### Cells

B16-OVA melanoma cells and MC38 colorectal cancer cells were cultured in DMEM medium (10% FBS, 100 U/mL penicillin and 100 μg/mL streptomycin), DC2.4 mouse dendritic cells were cultured in RPMI 1640 medium (10% FBS, 100 U/mL penicillin and 100 μg/mL streptomycin). NF-κB Reporter RAW 264.7 Cells were cultured in the complete heat-inactivated DMEM medium. These cell lines were maintained at 37°C with 5% CO_2_, authenticated by short tandem repeat (STR) profiling, and routinely tested for Mycoplasma contamination. Murine bone marrow-derived dendritic cells (BMDCs) and murine bone marrow-derived macrophages (BMDMs) were obtained by isolating bone marrow cells from surgically resected femur and tibia of C57BL/6 female mice (6–8 weeks). BMDCs were cultured in the complete heat-inactivated RPMI 1640 medium containing recombinant murine GM-CSF (20 ng/mL, PeproTech) at a density of 1.5 × 10^6^/well in 6-well plates on day 0. Similarly, BMDMs were cultured in the complete heat-inactivated DMEM medium containing recombinant murine M-CSF (20 ng/mL, PeproTech). Half the medium was replaced every 2 days. The cultured cells could be used for the following experiments on day 5 or day 6.

#### Mice

All animal studies were approved by the Institutional Animal Care and Use Committee (IACUC) and adhered to the guidelines of the Animal Ethics Committee of Peking University under protocol number (BCJB0076). Female C57BL/6J mice (6–8 weeks) were obtained from Peking University Health Science Center Department of Laboratory Animal Science (Beijing, China). NLRP3-KO mice (C57BL/6JGpt-*Nlrp*^*3em7Cd4411*^/Gpt, strain number T010873) and Pycard-KO mice (C57BL/6JGpt-*Pycardem1*^*Cd14740*^/Gpt, strain number T011758) were purchased from GemPharmatech LLC (Nanjing, China). Mice were maintained under specific pathogen-free (SPF) conditions and group-housed (maximum five per cage) under controlled temperature and humidity with a 12-h light/dark cycle. Food and water were provided *ad libitum*.

### Method details

#### Preparation of Nano-ZD and Nano-ZDM

The inner core of the nanoparticles was prepared by the water-in-oil microemulsion method as previously described.^24^^,^^26^ Briefly, aqueous solutions of Na_2_HPO_4_ (30 μL, 100 mM) and zoledronate (20 μL, 50 mM) and DOPA in chloroform (160 μL, 20 mM) were added dropwise to 4 mL of an organic mixed system (0.6 M Triton X-100/1.5M 1-hexanol in cyclohexane) and stirred for 30 min. Similarly, an aqueous solution of CaCl_2_ (50 μL, 500 mM) was added dropwise to 4 mL of the organic mixed system and stirred for 30 min. The bisphosphonates containing microemulsion were added dropwise to the CaCl_2-_containing microemulsion and stirred vigorously for 30 min at room temperature. After the addition of 16 mL of ethanol, the inner core was obtained by centrifugation at 12000 rpm for 10 min. The resulting pellet was washed twice with ethanol and once with ethanol/chloroform (1:1). The inner core was dispersed in chloroform. Nano-ZD was prepared by adding a chloroform solution of DOPC, cholesterol, and DSPE-PEG_2000_ (2000 μg, 285 μg, 1013 μg, molar ratio: 6.9 : 1.9: 1.2) to the inner core system. The organic solvent was removed by rotary evaporation at 37°C, and then preheated saline at 60°C was added for hydration. Then the mixture was sonicated at 60 w for 3 min using an ultrasonic cell disruptor (Scientz, Zhejiang, China). Nano-ZDM was formulated similarly but included MPLA (40 μg) in the lipid shell. To prepare Nano-ZD/Antigen nano-vaccine system, nanoparticles were mixed with antigen and swung on a shaker at 600 rpm for 30 min. As a comparison, Nano-HP was formulated in a similar manner of Nano-ZD but included the same molar amount of Na_2_HPO_4_ instead of zoledronate. Lipo-MPLA was formulated in a similar manner to Nano-ZDM but removed the inner core.

CaCl_2_·2H_2_O and Na_2_HPO_4_ were purchased from Sigma-Aldrich. Zoledronate monohydrate was purchased from Energy Chemical (Shanghai, China). MPLA (PHAD) and 18:1 PA (DOPA) were obtained from Avanti Polar Lipids. 1,2-Dioleoyl-*sn*-glycero-3-phosphocholine (DOPC) was purchased from Tokyo Chemical Industry Co., Ltd. DSPE-PEG_2000_ was purchased from NOF Corporation (Kawasaki, Japan). Cholesterol was obtained from HARVEYBIO (Beijing, China). (2,3-dioleoyloxy-propyl)-trimethylammonium-chloride (DOTAP) was purchased from APExBIO Technology.

#### Characterization of Nano-ZD and Nano-ZDM

The particle size and zeta potential were measured by Zetasizer Nano ZSP (Malvern, United Kingdom). The morphologies of nanoparticles were observed by a transmission electron microscope (JEM-1400 Plus, JEOL, Japan). Energy-dispersive X-ray spectroscopy of Nano-ZD-core was acquired by using high-resolution TEM with an EDS detector system (JEM-2100 F, JEOL, Japan). To determine zoledronate loading efficiency, Nano-ZD was digested in 1 M HCl solution for 24h. The mixture was vortexed and centrifuged at 15000 rpm for 15 min, and the supernatant was determined by HPLC (Shimadzu, Japan). To determine the release behaviors of zoledronate, nanoparticles were dispersed in media (pH 7.4 PBS, pH 5.0 PBS or DMEM with 10% FBS) and incubated at 37°C with a shaking speed of 100 rpm. At predetermined time points, the medium (20 μL) was taken out and centrifuged at 15000rpm for 15 min, and the supernatant was determined by HPLC analysis with an SB-C18 column (4.6 × 250, 5 μm). The temperature was maintained at 30°C with a flow rate of 0.5 mL min^−1^. The mobile phase was composed of 96% buffer A (dissolve 10.8 g of sodium octane-1-sulfonate and 37 mg of EDTA in water, add 10 mL of perchloric acid, 2 mL of phosphonic acid, and dilute to 1000 mL with water) and 4% buffer B (acetonitrile). The eluate was detected at 215 nm. The cytotoxicity of the various formulations was evaluated using the CCK8 assay.

#### *In vitro* cellular uptake assay

Intracellular transport of Nano-ZD was observed in DC 2.4 cells. Zol-AF647 (2‰ (w/w) of total Zol) and Dil (2‰ molar ratio of total lipids) were used to label the inner core and lipid shell of nanoparticles, respectively. The DC 2.4. cells were seeded on confocal dishes and cultured for 24 h, followed by incubation with Nano-ZD/Zol-AF647, Nano-ZD/Dil or Nano-ZD/Zol-AF647/Dil (10 μg/mL zoledronate) for 6 h. The nanoparticles were then removed. LysoTracker-Green was used to label the lysosomes, and Hoechst 33342 was used to stain the cell nucleus.OVA-Cy5 was used to detect antigen uptake by DCs. All samples were observed and photographed under a confocal microscope (AXR, Nikon). The raw images were processed by NIS-Elements viewer software (Nikon) and ImageJ.

#### Stimulation of BMDCs and BMDMs

BMDCs and BMDMs were cultured according to the protocol mentioned above. For free drugs activation assay, Cells (5 × 10^5^/well) were plated onto 48-well plates on day 5, and then treated with zoledronate or other bisphosphonates for 24 h. After that, the cells were washed and incubated with fresh complete heat-inactivated medium for 18 h or treated with LPS (40 ng/mL) for 18 h. The supernatants were collected for cytokine measurement by ELISA according to the manufacturer’s instructions. The cells were collected and stained with APC anti-mouse CD11c, PE anti-mouse CD86, PE/Cy7 anti-mouse CD80, PerCP/Cy5.5 anti-mouse MHCII, or FITC anti-mouse F4/80 antibodies according to the manufacturer’s instructions. Then the cells were analyzed by flow cytometer (CytoFLEX, Beckman Coulter, USA). For the Nano-ZD activation assay, cells were treated with Nano-ZD (10 μg/mL zoledronate) or Nano-HP (with the same concentration of Nano-ZD) for 24 h on day 5. After that, the cells were washed and incubated with fresh complete heat-inactivated medium for 18 h or treated with LPS (40 ng/mL) for 18 h. For the Nano-ZD/Antigen nano-vaccine system activation assay, BMDCs were treated with Nano-ZD/OVA, Lipo-MPLA/OVA or Nano-ZDM/OVA (10 μg/mL zoledronate, 2 μg/mL MPLA, 50 μg/mL OVA) for 24 h. Then the supernatants were collected for cytokine measurement. The cells were collected to detect cell surface co-stimulatory factors (CD11c, CD80, CD86, MHCII) or antigen presentation capacity (SIINFEKL-H-2Kb) by flow cytometry.

For poly I:C stimulation experiments, BMDCs were treated with zoledronate or Nano-ZD for 24 h (10 μg/mL zoledronate). After that, the cells were washed and transfected with poly I:C (5 μg/mL)/LyoVec for 18 h. The supernatants were collected for cytokine measurement.

For loss of function experiments, inhibitors were added 30 min before the stimulation of zoledronate or Nano-ZD. After that, the cells were washed and the inhibitors were added again before the treatment of LPS. Inhibitor concentrations were as follows, unless indicated otherwise: 5mM NAC, 10 μM α-Toc, 5 μM MitoQ, 5 μM GGPP, 10 μM FPP, 10 μM idebenone, 20 μg/mL cholesterol, 1 mM and 3 mM OA, 1mM and 3mM DHO. For gain of function experiments, MitoPQ was added 24 h before the stimulation of LPS. The concentration of chemical reagents was titrated, and the effective dose without cytotoxicity was used.

For pyrimidine supplementation, cells were cultured in cytidine, thymidine and uridine (100μM or 200 μM) for 24h before the treatment of zoledronate or Nano-ZD.

#### siRNA knockdown of BMDCs and BMDMs

siRNA knockdown was conducted by siRNA-LNP. We prepared siRNA-LNP using the ethanol injection method. The proportion of lipids used in LNP, namely DLin-MC3-DMA (MC3): Cholesterol: DSPC: DMG-PEG, is 50:38.5:10:1.5. Prepare 20 mM MC3, 20 mM Cholesterol, 5mM DSPC, and 1mM DMG-PEG stock solutions using anhydrous ethanol respectively. Take 6.30 μL, 4.85 μL, 5.04 μL, and 3.78 μL of these stock solutions and mix them. Dilute the mixture with anhydrous ethanol to 100 μL to obtain the total lipid ethanol mixture. Add 300 μL of sodium citrate buffer (pH 4.0) into a vial, then add siRNA (0.5nmol). Place it on a stirrer with the speed set at 1500 rpm. Quickly add 100 μL of the total lipid ethanol mixture into the aqueous buffer, stir at 1500 rpm for 10 min, and then let it stand for 10 min. Put the prepared system into a dialysis bag with a retention molecular weight of 3500 Da, and dialyze it in 2 L PBS (pH 7.4, 0.01 M) at 4°C for 2 h. Finally, filter the system to obtain the siRNA-LNP system.

BMDCs or BMDMs (1.5 × 10^6^/well) were plated onto 6-well plates on day 5 and transfected with MEFV siRNA or negative control siRNA (50 nM) for 48h. Knockdown efficiency was determined by western blot at 48 h post transfection. After that, the cells were treated with Nano-ZD (10 μg/mL zoledronate) for 24 h. Then, the cells were washed and stimulated with LPS (40 ng/mL) for 18h. The supernatants were collected for cytokine measurement.

#### *In vitro* T cell proliferation

For free drug activation assay, BMDCs were treated with zoledronate for 24h. After that, the cells were washed and treated with LPS/OVA (40 ng/mL LPS, 50 μg/mL OVA) for 18 h. Spleen lymphocytes from WT mice or OT-1 mice were isolated via a Mouse Spleen Lymphocyte Separation Reagent Kit (LTS1092PK, TBD) and labeled with CFSE. BMDCs and lymphocytes (1:1) at a density of 1 × 10^6^/well were co-cultured on 48-well plates for 72h. The cells were collected and stained with antibodies according to the manufacturer’s instructions. Then the cells were analyzed by flow cytometer. For Nano-ZD/Antigen nano-vaccine system activation assay, BMDCs were treated with Nano-ZD/OVA, Lipo-MPLA/OVA or Nano-ZDM/OVA (10 μg/mL zoledronate, 2 μg/mL MPLA, 50 μg/mL OVA) for 24 h and then co-cultured with T cells as mentioned above.

#### Mouse immunizations of free drugs

C57BL/6J mice were subcutaneously vaccinated on day 0 with OVA, zoledronate/OVA, Lipo-MPLA/OVA or Alum/OVA (2 μg OVA, 22 μg zoledronate, 5 μg MPLA, 100 μg Alum per mouse) and boosted on day 14. On day 21, the mice were sacrificed, and sera, draining lymph nodes and spleens were isolated for further analysis. Sera were collected for antibody titer test. Single cells were isolated from spleens and lymph nodes. Cells generated from the lymph nodes were stained with Pacific Blue anti-mouse CD45, AF488 anti-mouse CD11c, PE anti-mouse CD86, and PE/Cy7 anti-mouse CD80 antibodies. Cells generated from spleens were stained with Pacific Blue anti-mouse CD45, APC anti-mouse CD3, PE anti-mouse CD8α, and PE/Cy7 anti-mouse CD4 antibodies. Finally, the proportion of immune cells was analyzed by flow cytometer.

For the combination treatments of free zoledronate and MPLA, mice were randomized into three groups. In the zol + Lipo-MPLA/OVA group, mice were subcutaneously injected with free zoledronate (22 μg per mouse) on day 0 and day 14, and Lipo-MPLA/OVA (5 μg MPLA, 2 μg OVA) was injected one day after each zoledronate injection. In the (zol + MPLA Lipo)/OVA group, mice were subcutaneously injected with free zoledronate, Lipo-MPLA and OVA simultaneously on day 1 and day 15. In the Lipo-MPLA/OVA group, mice were immunized with Lipo-MPLA/OVA on day 1 and day 15. On day 22, sera were collected for the detection of OVA-specific IgG titers.

For anti-tumor therapy, C57BL/6J mice were subcutaneously inoculated with 1 × 10^5^ B16-OVA cells in the right flank on day 0. On day 8, mice were randomized into five groups. Mice were subcutaneously injected with Saline, OVA, Zol/OVA, Lipo-MPLA/OVA, or Alum/OVA (22 μg zoledronate, 5 μg MPLA, 100 μg Alhydrogel, 100 μg OVA per mouse per time) on day 8 and day 12. Tumor size (length and width) and body weight were measured every two days. The tumor volume was calculated as length × width × width × 0.5. On day 16, all tumors were harvested, weighed and imaged.

#### RNA-seq analysis

For free drug activation assay, BMDCs (5 × 10^6^) were treated with zoledronate (10 μg/mL) for 24 h. After that, the cells were washed and incubated with fresh complete heat-inactivated medium for 6 h or treated with LPS (40 ng/mL) for 6 h. The cells were pelleted and washed once with PBS at 1500 rpm, 4°C for 5 min and resuspended in 1 mL TRIzol reagent (Invitrogen). The samples were vortexed briefly every 3 min for 15 min and stored at −80°C until they were used for RNA extraction and library construction. For the Nano-ZD activation assay, cells were treated with Nano-ZD (10 μg/mL zoledronate) for 24 h, After that, the cells were washed and incubated with fresh complete heat-inactivated medium for 6 h or treated with LPS (40 ng/mL) for 6 h. The sample processing procedures were the same as above.

To investigate the effect of Nano-ZD and Nano-ZDM in DLNs, Nano-ZD or Nano-ZDM (22 μg zoledronate, 5 μg MPLA per mouse) were subcutaneously injected at the tail base on day 0. On day 3, two popliteal lymph nodes of each mouse were isolated and snap-frozen in liquid nitrogen for 15 min. To investigate the effect of Nano-ZD and Nano-ZDM in B16-OVA tumors, mice were subcutaneously injected with Nano-ZD/OVA or Nano-ZDM/OVA every 6 days for two times. Four days after the last treatment, 50 mg tumors of each mouse were collected. The tissues were stored at −80°C until they were used for RNA extraction and library construction.

Subsequent sample processing and RNA-Seq were performed at Biomarker Technologies Corporation (Beijing, China) and Novogene Corporation Inc. (Beijing, China). The raw reads were further analyzed and processed by the bioinformatic pipeline tool BMKCloud (www.biocloud.net) https://www.bioinformatics.com.cn.

#### Biodistribution of Nano-ZD

To investigate the retention of nanoparticles at the injection sites, C57BL/6J mice were subcutaneously injected at the tail base with free Zol-AF647 or Nano-ZD/Zol-AF647 (22 μg zoledronate, 200 ng Zol-AF647 per mouse). After 0, 4, 8, 24, 48, and 72 h, fluorescence imaging at the tail base was carried out with the IVIS spectrum imaging system (PerkinElmer). To analyze the accumulation of nanoparticles in DLNs, C57BL/6J mice were subcutaneously injected with free Zol-AF647 or Nano-ZD/Zol-AF647. At 4, 8, 24, 48, and 72 h after administration, three mice were sacrificed and DLNs (popliteal lymph nodes) were collected. Fluorescence imaging of DLNs was carried out with IVIS spectrum imaging system. To analyze the accumulation of antigen in DLNs, C57BL/6J mice were subcutaneously injected with free OVA-Cy5, Nano-ZD/OVA-Cy5 or Nano-ZDM/OVA-Cy5 (10 μg OVA-Cy5 per mouse). After 24 h, DLNs were collected and imaged with IVIS Spectrum imaging system. The fluorescence intensity of tissues was quantified by Living Image software. Meanwhile, the injection sites and DLNs were prepared into frozen sections, sealed with a mounting medium containing hochest33342, followed by detection via the Vectra Polaris analysis system (PerkinElmer).

To determine the concentration of zoledronate in bone tissue and DLNs, samples were analyzed by HPLC. Mice were subcutaneously injected with free zoledronate (200 μg per mouse). Bones (femurs and tibias) and DLNs were collected at 20 min, 4 h and 24 h post injection. PBS was added to bone sample (0.5 mL) or DLNs sample (0.2 mL) tubes of each mouse followed by homogenization at −10°C for 30 min. Next, sample tubes were centrifuged at 10000 rpm, 4°C for 15 min. The supernatant was collected and mixed with an equal volume of acetonitrile to precipitate proteins. Sample tubes were centrifuged at 15000 rpm, 4°C for 20 min. The supernatant was determined by HPLC analysis with an SinoChrom ODS-BP column (4.6 × 250, 5 μm). The temperature was maintained at 30°C with a flow rate of 1 mL min^−1^. The mobile phase was composed of 75% buffer A (dissolve 2.30 g ammonium dihydrogen phosphate and 5mL tetrabutylammonium hydroxide in ultra-pure water, fixing to 1000 mL, and adjusting to pH = 2.10 with phosphoric acid) and 25% buffer B (acetonitrile). The eluate was detected at 218 nm.

#### FRET analysis of Nano-ZD integrity

To investigate the integrity of nanoparticles *in vivo*, Sulfo-Cy3 (5 nmol) and Sulfo-AF647 (5 nmol) at a molar ratio of 1:1 were incorporated into the core of Nano-ZD to construct Nano-ZD_Cy3/AF647_ with FRET efficiency. Sulfo-Cy3 (5 nmol) was incorporated into the core of Nano-ZD to construct Nano-ZD_Cy3._ C57BL/6J mice were subcutaneously injected with Nano-ZD_Cy3/AF647_ or Nano-ZD_Cy3_. After 4 h, DLNs were collected and prepared into frozen sections. The fluorescence and FLIM images were acquired with an STELLARIS 8 confocal microscope platform (Leica, Germany).

#### Nanoparticle uptake in lymph nodes

C57BL/6J mice were subcutaneously injected with Nano-ZD/Zol-AF647. At 24 h and 48 h after administration, DLNs were removed for analysis. Single cells were isolated from lymph nodes and stained with Pacific Blue anti-mouse CD45, PE/Cy7 anti-mouse CD11c, PE/Dazzle 594 anti-mouse CD11b, AF488 anti-mouse B220, and PE anti-mouse F4/80 antibodies. Finally, the proportion of AF647^+^ cells in different types of immune cells was analyzed by flow cytometer.

#### Activation of APCs in lymph nodes

C57BL/6J mice were subcutaneously injected with a single dose of OVA, Nano-HP/OVA, Nano-ZD/OVA, Lipo-MPLA/OVA and Nano-ZDM/OVA (100 μg OVA, 22 μg zoledronate and 5 μg MPLA per mouse). After 4 days, the DLNs were harvested and mechanically dissociated. Single cells were divided into two parts. One part was stained with Pacific Blue anti-mouse CD45, APC anti-mouse CD11c, PE anti-mouse CD86 and PE/Cy7 anti-mouse CD80 antibodies to estimate the expression of co-stimulatory molecules. The other part was stained with APC anti-mouse CD11c and PE anti-mouse SIINFEKL-H-2Kb antibodies to detect the antigen presentation capacity.

#### Analysis of humoral immune responses

OVA was used as the model antigen to evaluate the activation of B cells and T follicular helper (Tfh) cells. Mice were subcutaneously injected with OVA, Nano-HP/OVA, Nano-ZD/OVA, Lipo-MPLA/OVA, Nano-ZDM/OVA or Alum/OVA on day 0 and day 14 (22 μg zoledronate, 5 μg MPLA, 100 μg Alhydrogel, 2 μg OVA per mouse per time). To enhance the binding affinity with the antigen, DOTAP was incorporated at a molar ratio of 10% during the fabrication of the lipid layer in Nano-HP, Nano-ZD, Lipo-MPLA and Nano-ZDM groups. On day 21, DLNs and sera were collected. Sera were used to detect the titers of anti-OVA IgG antibodies. Single-cell suspensions from DLNs were counted and divided into two parts. One part was stained with Pacific Blue anti-mouse CD45, PE/Cy5 anti-mouse B220 and PE anti-mouse GL7 antibodies to detect GC B cells. The other part was stained with FITC anti-mouse CD3, PE/Cy7 anti-mouse CD4, APC anti-mouse CD8α, PE anti-mouse PD-1, BV421 anti-mouse CXCR5 antibodies to detect Tfh cells. For HA1 immunization, mice were subcutaneously injected with HA1, Nano-HP/HA1, Nano-ZD/HA1, Lipo-MPLA/HA1, Nano-ZDM/HA1, or Alum/HA1 on day 0 and day 14 (2 μg HA1 per mouse per time). Sera were collected and used to detect the titers of anti-HA1 IgG antibodies on day 21, day 28, and day35. For Spike S1 and HPV 16 immunization, mice were subcutaneously injected with Antigen, Nano-ZD/antigen, Nano-ZDM/antigen, or Alum/antigen on day 0 and day 14 (2 μg antigen per mouse per time). Sera were collected and used to detect the titers of specific IgG antibodies on day 21, day 28, and day35.

#### Antibody titer test

Sera were collected from whole blood by centrifugation, the levels of antigen-specific IgG, IgG1 and IgG2c were measured by ELISA. Briefly, 96-well plates (Nunc MaxiSorp, 423501, BioLegend) were coated with 2 μg/mL antigens (OVA, HA1, Spike S1, or HPV16) in 50mM carbonate buffer at 4°C overnight (100 μL/well). After washing 3 times with washing buffer (0.05% Tween 20 in PBS), plates were blocked with 200 μL of block buffer (2% BSA in PBS) for 2 h at 37°C. After washing 4 times, 100 μL of diluted samples was added to plates and incubated overnight at 4°C. After washing 4 times, the plates were incubated with HRP-conjugated goat anti-mouse IgG, IgG1 or IgG2c for 1 h at 37°C. After washing 5 times, the plates were incubated with 100 μL of TMB buffer (Solaibao, China) for 15 min, followed by adding 100 μL of stop solution (2N H_2_SO_4_). Optical densities (OD) were determined at a wavelength of 450 nm by a microplate reader (Thermo). Antibody titers were obtained by plotting the maximum serum dilution that gave an OD > 2 × background.

#### Tumor therapeutic efficacy analysis

*Subcutaneous B16-OVA tumor-bearing mice*. C57BL/6J mice were inoculated with 5 × 10^4^ B16-OVA cells in the right flank on day 0. On day 7, mice were randomized into six groups. Mice were subcutaneously injected with saline, Nano-HP/OVA, Nano-ZD/OVA, Lipo-MPLA/OVA, Nano-ZDM/OVA or Alum/OVA (22 μg zoledronate, 5 μg MPLA, 100 μg Alhydrogel, 100 μg OVA per mouse per time) on day 7, day 11 and day 15. Tumor size (length and width) and body weight were measured every two days. Death was captured when tumor volume was larger than 1500 mm^3^ or mice died naturally. On day 60, tumor-free mice of Nano-ZD/OVA group and Nano-ZDM/OVA group were rechallenged with 5 × 10^4^ B16-OVA cells in the left flank. On day 19, sera were collected from whole blood to detect cytokine secretion and the titers of anti-OVA IgG1 and IgG2c. And the DLNs were harvested and mechanically dissociated. Single-cell suspensions of DLNs (1×10^6^/well) were seeded in a 24-well plate in the complete heat-inactivated RPMI 1640 medium. After 24 h, the supernatants were collected to detect cytokine secretion.

*Subcutaneous MC38 tumor-bearing mice.* C57BL/6J mice were inoculated with 1 × 10^6^ MC38 cells in the right flank. Five days after tumor inoculation, mice were treated with saline, Lipo-MPLA/Adpgk, Nano-ZD/Adpgk and Nano-ZDM/Adpgk every five days for three times (22 μg zoledronate, 5 μg MPLA, 50 μg Adpgk per mouse per time). Groups receiving checkpoint blockade were intraperitoneally injected with 100 μg αPD-L1(clone10F.9G2, Bio X Cell) on days 3, 7, and 12 after tumor inoculation. Tumor volume and body weight of mice were monitored as described above. All tumors were harvested, weighed and imaged 4 days after the last treatments.

#### Cytotoxic T cell activation analysis

To evaluate cytotoxic T cell activation, C57BL/6J mice were subcutaneously injected with a single dose of saline, Nano-HP/OVA, Nano-ZD/OVA, Lipo-MPLA/OVA and Nano-ZDM/OVA (100 μg OVA, 22 μg zoledronate and 5 μg MPLA per mouse). After 4 days, single cell suspensions of spleens (1×10^6^/well) were seeded on a 24-well plate in the complete heat-inactivated RPMI 1640 medium supplemented with Cell Stimulation Cocktail (Invitrogen). After 6 h, the cells were washed and stained with Pacific Blue anti-mouse CD45, FITC anti-mouse CD3, APC anti-mouse CD8α and BV605 anti-mouse IFN-γ antibodies. Intracellular staining was performed using an intracellular staining permeabilization wash buffer (BioLegend). The percentages of IFN-γ^+^ in CD8^+^ T cells were quantified by flow cytometer.

#### T lymphocyte infiltration in tumors

To evaluate T lymphocyte infiltration in tumors, C57BL/6J mice were inoculated with 1 × 10^5^ B16-OVA cells in the right flank on day 0. When the average tumor reached ∼100 mm^3^, mice were subcutaneously injected with saline, Nano-HP/OVA, Nano-ZD/OVA, Lipo-MPLA/OVA and Nano-ZDM/OVA every 6 days for 2 times (100 μg OVA, 22 μg zoledronate and 5 μg MPLA per mouse per time). Nine days after the last treatments, B16-OVA melanomas were dissected and prepared into single-cell suspensions. Lymphocytes were isolated using the Mouse Tumor Infiltrating Tissue Lymphocyte Isolation Solution Kit (Cat. #P9000; Solarbio, China). The cells were stained with Pacific Blue anti-mouse CD45, FITC anti-mouse CD3, APC anti-mouse CD8α, PE/Cy7 anti-mouse CD4, PerCP/Cy5.5 anti-mouse CD25, PE anti-mouse FOXP3 antibodies, and then detected by flow cytometry. Intracellular staining was performed using an intracellular staining permeabilization wash buffer (BioLegend). Meanwhile, tumor tissues were prepared into frozen sections and stained with rabbit anti-mouse CD8 antibody, followed by Daylight 649 Goat anti-rabbit secondary antibody.

#### Full-spectrum flow cytometry analysis

The administration of drugs to mice was performed as described in the previous section. Four days after the last treatment, B16-OVA melanomas were dissected and prepared into single-cell suspensions. Leukocytes were isolated using the Mouse Tumor Infiltrating Leukocyte Isolation Kit (Cat. #P1910, Solarbio, China). The cells were then stained with 18 different antibodies. Finally, the stained cells were filtered through a strainer, acquired on the ID7000 spectral flow cytometer(Sony, Japan), and analyzed using Flowjo.

#### Tetramer staining

H2-K^b^/OVA tetramer assays were used to detect OVA-specific CD8^+^ T cells. C57BL/6J mice were inoculated with 1 × 10^5^ B16-OVA cells on day 0. Seven days after tumor inoculation, mice were treated with saline, αPD-L1, Nano-ZDM/OVA or Nano-ZDM/OVA + αPD-L1 (22 μg zoledronate, 5 μg MPLA, 100 μg OVA, 100 μg αPD-L1 per mouse per time). αPD-L1 was treated on day 7, day 12 and day 17. Nano-ZDM/OVA was treated on day 9 and day 15. On day 20, tumors and spleens were harvested and processed into single-cell suspensions. Tumor-infiltrating lymphocytes were isolated using a commercial kit. The resulting cells were divided into two aliquots: one was used to analyze CD8^+^IFN-γ^+^ and CD8^+^Granzyme B^+^ populations, while the other was subjected to tetramer staining. Briefly, this aliquot was first stained with a live/dead dye (Ghost Dye Red 780, CYTEK). After washing, the cells were incubated with 2.5 μL PE-labeled H2-Kb/OVA (SIINFEKL) tetramers at 4°C for 60 min, followed by staining with a surface antibody cocktail containing PB anti-mouse CD45, APC anti-mouse CD3, and FITC anti-mouse CD8 (Cat. #K0227-4; MBL, Japan).

#### Lung metastasis prophylactic study

To evaluate the preventive efficacy of lung metastasis, mice were subcutaneously injected with saline, Nano-HP/OVA, Nano-ZD/OVA, Lipo-MPLA/OVA, Nano-ZDM/OVA or Alum/OVA every 6 days for 2 times (22 μg zoledronate, 5 μg MPLA, 100 μg Alhydrogel, 100 μg OVA per mouse per time). After six days, mice were intravenously injected with 2 × 10^5^ B16-OVA cells. After three weeks, the mice were sacrificed and the lungs were dissected. The nodules of lung metastasis were counted.

#### Memory T lymphocyte activation

To evaluate the activation of memory T lymphocyte, B16-OVA tumor bearing mice were subcutaneously injected with saline, Nano-ZD/OVA, Lipo-MPLA/OVA and Nano-ZDM/OVA every 6 days for 2 times (100 μg OVA, 22 μg zoledronate and 5 μg MPLA per mouse per time). Nine days after the last treatments, the spleens were dissected to prepare a single-cell suspension. Single cells were stained with Pacific Blue anti-mouse CD45, FITC anti-mouse CD3, APC anti-mouse CD8α, PE/Cy7 anti-mouse CD4, PE/Cy5 anti-mouse/human CD44 and PE anti-mouse CD62L antibodies, CD44^+^CD62L^low^ effector memory T cells and CD44^+^CD62L^high^ central memory T cells within CD8^+^ and CD4^+^ T cell were measured by flow cytometry.

#### Western blot analysis

For protein levels in whole cell extracts, BMDCs or BMDMs were lysed with RIPA lysis buffer (Beyotime, China) The extracted proteins were quantified via a BCA Protein Assay kit, fractionated on 4–20% SDS-PAGE gel electrophoresis and transferred to 0.45 μm PVDF membranes. After being blocked with QuickBlock Blocking Buffer (Beyotime, China), the transferred membrane was stained with primary antibodies including rabbit anti-IL-1β (ab234437, Dilution 1:1000), rabbit anti-Pyrin (ab195975, Dilution 1:1000), rabbit anti-NF-κB p65 (ab32536, Dilution 1:1000), rabbit anti-phospho-NF-κB p65 (Ser536) (3033T, Dilution 1:1000), rabbit anti-IKBα (ab32518, Dilution 1:1000), rabbit anti-phospho-IKBα (phospho S32) (2859T, Dilution 1:1000), rabbit anti-NLRP3 (ab263899, Dilution 1:1000), rabbit anti-ASC (ab307560, Dilution 1:1000), rabbit anti-RhoA (ab187027, Dilution 1:5000), rabbit anti-GAPDH (10494-1-AP, Dilution 1:20000) Rabbit anti-Sodium Potassium ATPase (ab76020, Dilution 1:5000) and mouse anti-β-tubulin (66240-1-lg, 1:100000) overnight at 4°C, and followed by HRP-conjugated goat anti-mouse (ab6789, Dilution 1:10,000) and goat anti-rabbit (ab6721, Dilution 1:10,000) secondary antibodies for 2 h. Finally, the protein bands were processed by ECL chemiluminescence.

For protein levels in mitochondria, mitochondria were isolated by using the Mitochondria isolation kit (Beyotime, China). Briefly, BMDCs were harvested and washed once with PBS by centrifugation at 600 × g for 5 min at 4°C. Then the cells were resuspended in a mitochondrial isolation buffer for 15 min at 4°C, and subjected to Dounce-homogenization to lyse the cells. Next, the homogenate was centrifuged at 600 × g for 10 min at 4°C, and the supernatant was collected and centrifuged at 11000 × g for 10 min at 4°C to obtain the crud mitochondria in the pellet. Then, the crude mitochondria were lysed with mitochondrial lysis fluid in the kit. The extracted proteins were subjected to SDS-PAGE, and immunoblot analysis was performed with the indicated antibodies including rabbit anti-MAVS (ab314444, Dilution 1:1000) and mouse anti-COXIV (11967T, Dilution 1:1000).

Membrane protein and cytoplasmic protein were separated with the membrane protein extraction kit (Cat. # EX1110; Solarbio, China) according to the manufacturer’s instructions.

#### NF-κB agonistic activity on reporter cells

The NF-κB reporter RAW264.7 cells were cultured in a 96-well plate with 50,000 cells/well in the complete heat-inactivated DMEM medium. After incubation with zoledronate or Nano-ZD for 24 h, the supernatant was removed. The cells were stimulated with LPS (40 ng/mL) for 6 h. After that, 50 μL of supernatant was collected from each well and mixed with 150 μL chromogenic substrate Quanti-blue solution (InvivoGen). The absorbance was measured by a Microplate Reader (Multiskan FC, Thermo Fisher Scientific) at 620 nm.

#### Measurement of mitochondrial ROS

BMDCs were treated with zoledronate or Nano-ZD (10 μg/mL zoledronate) for 24 h. After that, the cells were washed and treated with LPS (40 ng/mL) for 0.5, 4, 6, 12, and 24 h. Next, the cells were collected and stained with 2.5 μM MitoSOX Red (Thermo) for 20 min at 37°C according to the manufacturer’s instructions. ROS signal was analyzed by flow cytometer (CytoFLEX, Beckman Coulter, USA).

#### Analysis of mitochondrial morphology

BMDCs were seeded on PLL-coated confocal dishes on day 5 and treated with zoledronate or Nano-ZD for 24 h followed by LPS for 12 h. Then, the cells were stained with MitoTracker Deep Red (Thermo) for 30 min at 37°C according to the manufacturer’s instructions. All samples were observed and photographed under a confocal microscope (AXR, Nikon) with a 63× oil objective lens. Mitochondrial morphology was analyzed by an ImageJ macro (MINA).

#### Immunofluorescence staining of MAVS

BMDCs were seeded on PLL-coated confocal dishes followed by different treatments. The cells were fixed, exposed to anti-MAVS antibody (ab314444 Dilution 1:1000) overnight at 4°C, and then stained with Dylight 649 Goat anti-Rabbit secondary antibody (A23620, Dilution 1:1000) for 2 h. All samples were observed and photographed under a confocal microscope (AXR, Nikon) with a 63× oil objective lens. The raw images were processed by NIS-Elements viewer software (Nikon) and ImageJ.

#### SDD-AGE analysis of MAVS aggregation

SDD-AGE was used to analyze the aggregation of MAVS according to a published protocol.^46^ Crude mitochondria were isolated by the Mitochondria isolation kit (Beyotime) as mentioned above. Then, the mitochondria were resuspended in sample buffer (0.5 × TBE, 10% glycerol, 2% SDS, and 0.0025% bromophenol blue) and loaded onto a 1.5% agarose gel containing 1 × TBE and 0.1% SDS. The gel was run in a running buffer (1 × TBE and 0.1% SDS) at 75 V for 60 min at 4°C. Then, the proteins were transferred to PVDF membranes for immunoblotting.

#### LC-MS/MS analysis of CoQ9

CoQ9 was extracted from cells using an extraction buffer (MeOH/Isopropanol, 1:1, v/v). The extraction buffer was prepared fresh for each extraction. The entire experimental process was carried out on ice. BMDCs with different treatments were collected, washed with pre-cooled PBS, and counted. One milliliter of pre-cooled extraction buffer was added to each sample (1×10^6^ cells), and samples were sonicated at 100 W for 2 min. The samples were then centrifuged at 15,000 rpm for 30 min at 4°C twice to collect the supernatant. Then, 2 μL of supernatant was injected for LC-MS/MS analysis. The samples were analyzed using a Waters ACQUITY UPLC i-Class Plus instrument. A Waters Xevo TQ-S instrument, equipped with an electrospray ion source (ESI), was used in positive ion mode. Data were collected using Waters MassLynx V4.2. The detailed experimental procedures for LC-MS were carried out by the technical staff of the State Key Laboratory of Natural and Biomimetic Drugs.

#### Untargeted metabolomics analysis

BMDCs (1 × 10^6^) were treated with Nano-ZD (10 μg/mL zoledronate) or PBS for 24 h. The cells were pelleted and washed twice with PBS at 1000 g, 4°C for 1 min. Then the pellets were snap-frozen in liquid nitrogen for 15 min and stored at −80°C. Liquid chromatography with tandem mass spectrometry metabolomics was carried out by Novogene Corporation Inc. (Beijing, China). The raw data were further processed by https://www.bioinformatics.com.cn, an online platform for data analysis and visualization.

#### Metabolic analysis

Real-time oxygen consumption rate (OCR) was measured using a Seahorse XF96 Pro Extracellular Flux Analyzer (Agilent). BMDCs were treated with zoledronate or Nano-ZD (10 μg/mL zoledronate) for 24 h. After that, the cells were washed and treated with LPS (40 ng/mL) for 12 h. Then, BMDCs (8 × 10^4^/well) with different treatments were seeded on a PLL-coated XF microplate. The assay was performed in XF RPMI assay medium supplemented with 10 mM glucose, 1 mM pyruvate and 2 mM glutamine. Three consecutive OCR measurements were performed under basal conditions and after the sequential addition of 2.5 μM oligomycin, 2 μM FCCP, 1 μM rotenone and 1 μM antimycin. ATP concentrations of cells were determined with a CellTiter-Lumi Plus kit according to the manufacturer’s instructions (Cat.#C0068S, Beyotime). The NAD^+^/NADH ratio was assayed using a NAD^+^/NADH assay kit with WST-8 (Cat.#S0175, Beyotime) according to the manufacturer’s instructions.

### Quantification and statistical analysis

Data are presented as mean ± s.d. or mean ± s.e.m., as indicated in the figure legends. Statistical details for each experiment, including exact *n* values and what n represents, are provided in the figure legends. Statistical significance was determined using unpaired two-tailed Student’s *t* test for comparisons between two groups, one-way or two-way analysis of variance (ANOVA) followed by Tukey’s multiple-comparisons test for comparisons among multiple groups, and the log rank test for survival analyses. Exact *p* values were provided in the figures. A *p* value of less than 0.05 was considered statistically significant. All statistical analyses were performed in GraphPad Prism 8.0.

## Supplemental information

Document S1. Figures S1–S27 and Table S1

Document S2. Article plus supplemental information
