# Supplementary material for: Nano-granulated zoledronate sensitizes innate immune metabolism to enhance vaccine-induced and antitumor immunity
Source: Cell Rep Med. 2026 Apr 22;7(5):102765. doi: 10.1016/j.xcrm.2026.102765 (PMC13198249; doi:10.1016/j.xcrm.2026.102765)
Supplement: Document S1. Figures S1–S27 and Table S1 [file mmc1.pdf]

Cell Reports Medicine, Volume *7*

Supplemental information

Nano-granulated zoledronate sensitizes innate immune metabolism to enhance vaccine-induced and antitumor immunity

Meifang Chen, Xiaojia Jiao, Zhicheng Yan, Yueheng Wang, Jing Zhang, Qinghua Wu, Minghui Li, Shumin Fan, Yuan Wang, Wenbing Dai, Hua Zhang, Xueqing Wang, Qiang Zhang, and Bing He

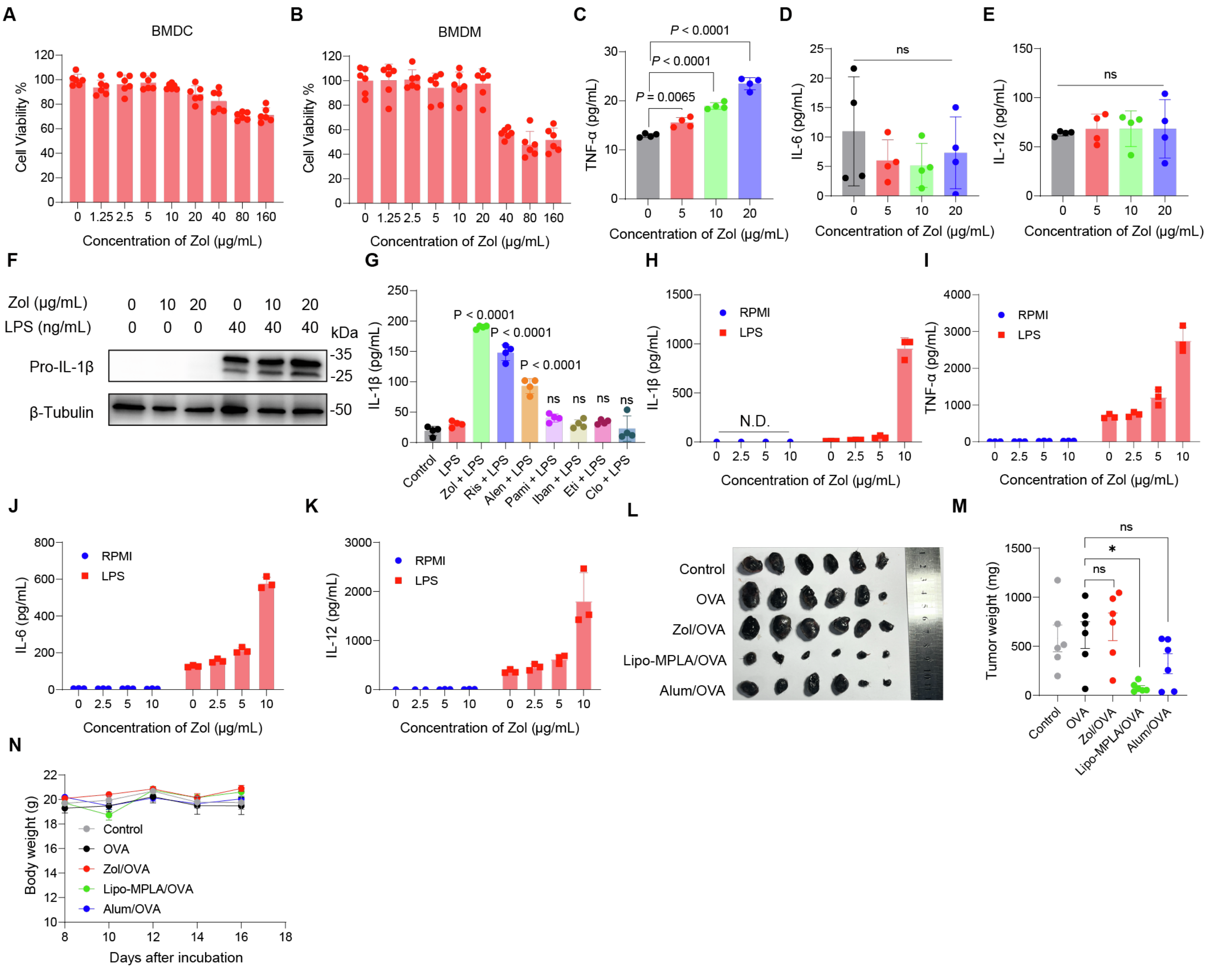

Figure S1. Free zoledronate stimulates and sensitizes innate immune response in vitro but not in vivo. Related to Figure 1. **(A-B)** Cell viability of BMDCs **(A)** and BMDMs **(B)** after the treatments with different concentrations of zoledronate for 24 h (n = 6). **(C-E)** ELISA detections of TNF-α **(C)**, IL-6 **(D)** and IL-12 **(E)** in the supernatant of BMDCs after the treatments of different concentrations of free zoledronate (n = 4). **(F)** Western blot analysis of pro-IL-1β protein expression in the lysates of BMDCs after different treatments. β-tubulin was used as a loading control. **(G)** The expression of IL-1β in the supernatant of BMDCs after different bisphosphonates treatments at the same molar concentration (36 μM, n = 4). **(H-K)** ELISA detections of IL-1β **(H)**, TNF-α **(I)**, IL-6 **(J)** and IL-12 **(K)** in the supernatant of BMDMs after different treatments (n = 3). **(L-M)** Weight and photographs of excised B16-OVA tumors after different treatments (n = 6). (**N**) Body weight changes of tumor-bearing mice during the treatments (n = 6). Data are shown as mean ± s.d.

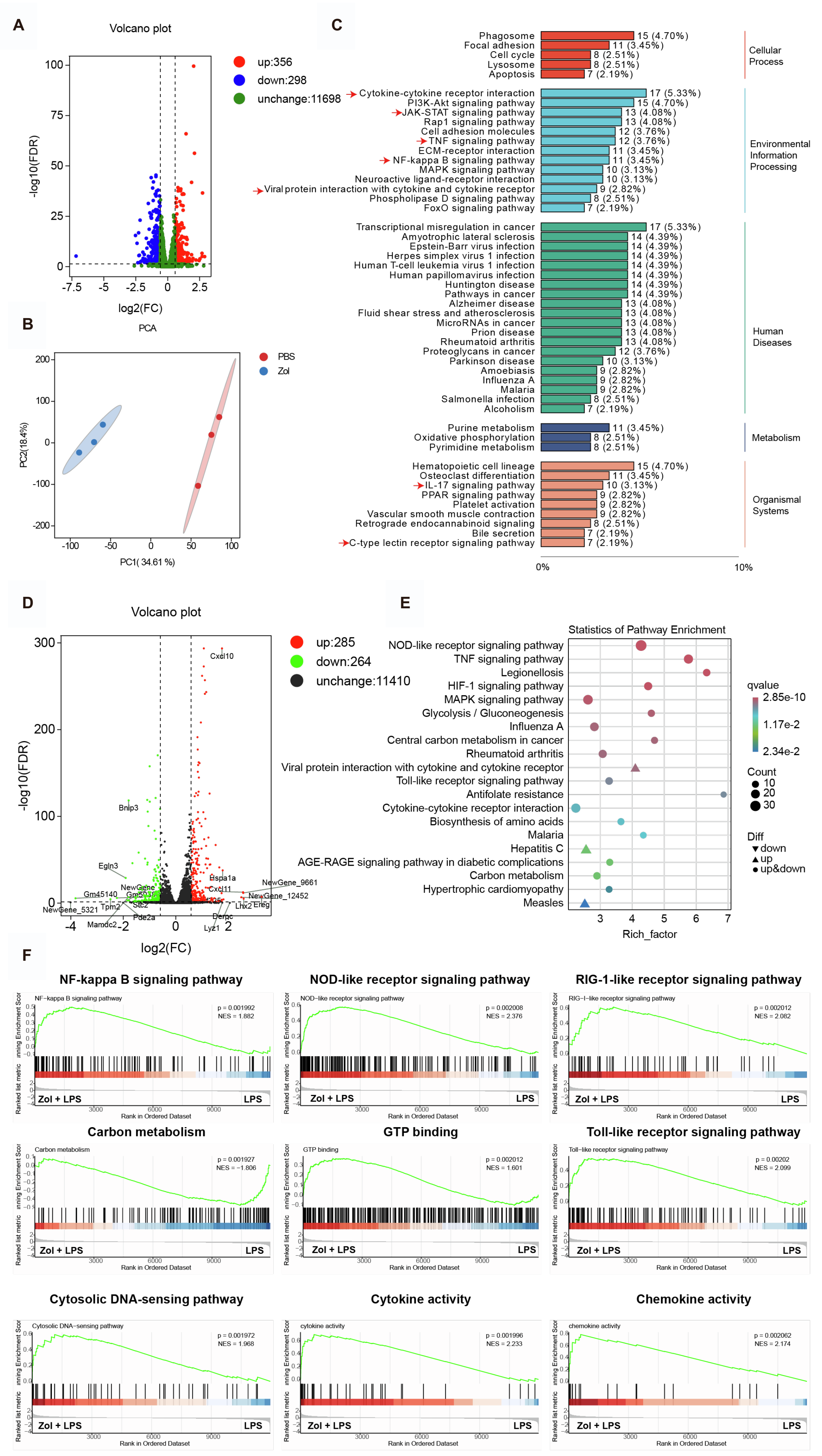

A
Volcano plot
up:356
down:298
unchange:11698
-log10(FDR)
log2(FC)
B
PCA
PBS
Zol
PC2(18.4%)
PC1(34.61 %)
C
Phagosome 15 (4.70%)
Focal adhesion 11 (3.45%)
Cell cycle 8 (2.51%)
Lysosome 8 (2.51%)
Apoptosis 7 (2.19%)
Cellular Process
Cytokine-cytokine receptor interaction 17 (5.33%)
PI3K-Akt signaling pathway 15 (4.70%)
JAK-STAT signaling pathway 13 (4.08%)
Rap1 signaling pathway 13 (4.08%)
Cell adhesion molecules 12 (3.76%)
TNF signaling pathway 12 (3.76%)
ECM-receptor interaction 11 (3.45%)
NF-kappa B signaling pathway 11 (3.45%)
MAPK signaling pathway 10 (3.13%)
Neuroactive ligand-receptor interaction 10 (3.13%)
Viral protein interaction with cytokine and cytokine receptor 9 (2.82%)
Phospholipase D signaling pathway 8 (2.51%)
FoxO signaling pathway 7 (2.19%)
Environmental Information Processing
Transcriptional misregulation in cancer 17 (5.33%)
Amyotrophic lateral sclerosis 14 (4.39%)
Epstein-Barr virus infection 14 (4.39%)
Herpes simplex virus 1 infection 14 (4.39%)
Human T-cell leukemia virus 1 infection 14 (4.39%)
Human papillomavirus infection 14 (4.39%)
Huntington disease 14 (4.39%)
Pathways in cancer 14 (4.39%)
Alzheimer disease 13 (4.08%)
Fluid shear stress and atherosclerosis 13 (4.08%)
MicroRNAs in cancer 13 (4.08%)
Prion disease 13 (4.08%)
Rheumatoid arthritis 13 (4.08%)
Proteoglycans in cancer 12 (3.76%)
Parkinson disease 10 (3.13%)
Amoebiasis 9 (2.82%)
Influenza A 9 (2.82%)
Malaria 9 (2.82%)
Salmonella infection 8 (2.51%)
Alcoholism 7 (2.19%)
Human Diseases
Purine metabolism 11 (3.45%)
Oxidative phosphorylation 8 (2.51%)
Pyrimidine metabolism 8 (2.51%)
Metabolism
Hematopoietic cell lineage 15 (4.70%)
Osteoclast differentiation 11 (3.45%)
IL-17 signaling pathway 10 (3.13%)
PPAR signaling pathway 9 (2.82%)
Platelet activation 9 (2.82%)
Vascular smooth muscle contraction 9 (2.82%)
Retrograde endocannabinoid signaling 8 (2.51%)
Bile secretion 7 (2.19%)
C-type lectin receptor signaling pathway 7 (2.19%)
Organismal Systems
0%
10%
D
Volcano plot
up:285
down:264
unchange:11410
-log10(FDR)
log2(FC)
Cxcl10
Bnip3
Egln3
Hspa1a
Cxcl11
NewGene_9661
NewGene_12452
Lhx2
Ereg
Derpc
Lyz1
Gm45140
NewGene_5321
Tpm2
Mamdc2
Stc2
Pde2a
E
Statistics of Pathway Enrichment
NOD-like receptor signaling pathway
TNF signaling pathway
Legionellosis
HIF-1 signaling pathway
MAPK signaling pathway
Glycolysis / Gluconeogenesis
Influenza A
Central carbon metabolism in cancer
Rheumatoid arthritis
Viral protein interaction with cytokine and cytokine receptor
Toll-like receptor signaling pathway
Antifolate resistance
Cytokine-cytokine receptor interaction
Biosynthesis of amino acids
Malaria
Hepatitis C
AGE-RAGE signaling pathway in diabetic complications
Carbon metabolism
Hypertrophic cardiomyopathy
Measles
Rich_factor
qvalue
2.85e-10
1.17e-2
2.34e-2
Count
10
20
30
Diff
down
up
up&down
F
NF-kappa B signaling pathway
p = 0.001992
NES = 1.882
NOD-like receptor signaling pathway
p = 0.002008
NES = 2.376
RIG-1-like receptor signaling pathway
p = 0.002012
NES = 2.082
Carbon metabolism
p = 0.001927
NES = −1.806
GTP binding
p = 0.002012
NES = 1.601
Toll-like receptor signaling pathway
p = 0.00202
NES = 2.099
Cytosolic DNA-sensing pathway
p = 0.001972
NES = 1.968
Cytokine activity
p = 0.001996
NES = 2.233
Chemokine activity
p = 0.002062
NES = 2.174
Zol + LPS
LPS
Rank in Ordered Dataset

Figure S2. RNA-seq analysis of BMDCs after different treatments. Related to Figure 1. (A-C) The control group is PBS, the experimental group is free zoledronate (Zol). (n = 3) **(A)** Volcano plots of differentially expressed genes (DEGs) between PBS and Zol groups. **(B)** Principal component analysis (PCA) of all samples. **(C)** KEGG pathways annotation analysis of DEGs between PBS and Zol groups. The red arrows point to inflammation-related pathways. **(D-F)** The control group is LPS, the experimental group is Zol + LPS. (n = 3) **(D)** Volcano plots of DEGs between LPS and Zol + LPS groups. **(E)** KEGG enrichment analysis of DEGs between LPS and Zol + LPS groups. **(F)** Transcript Gene set enrichment analysis (GSEA) of Zol +LPS group compared with LPS group.

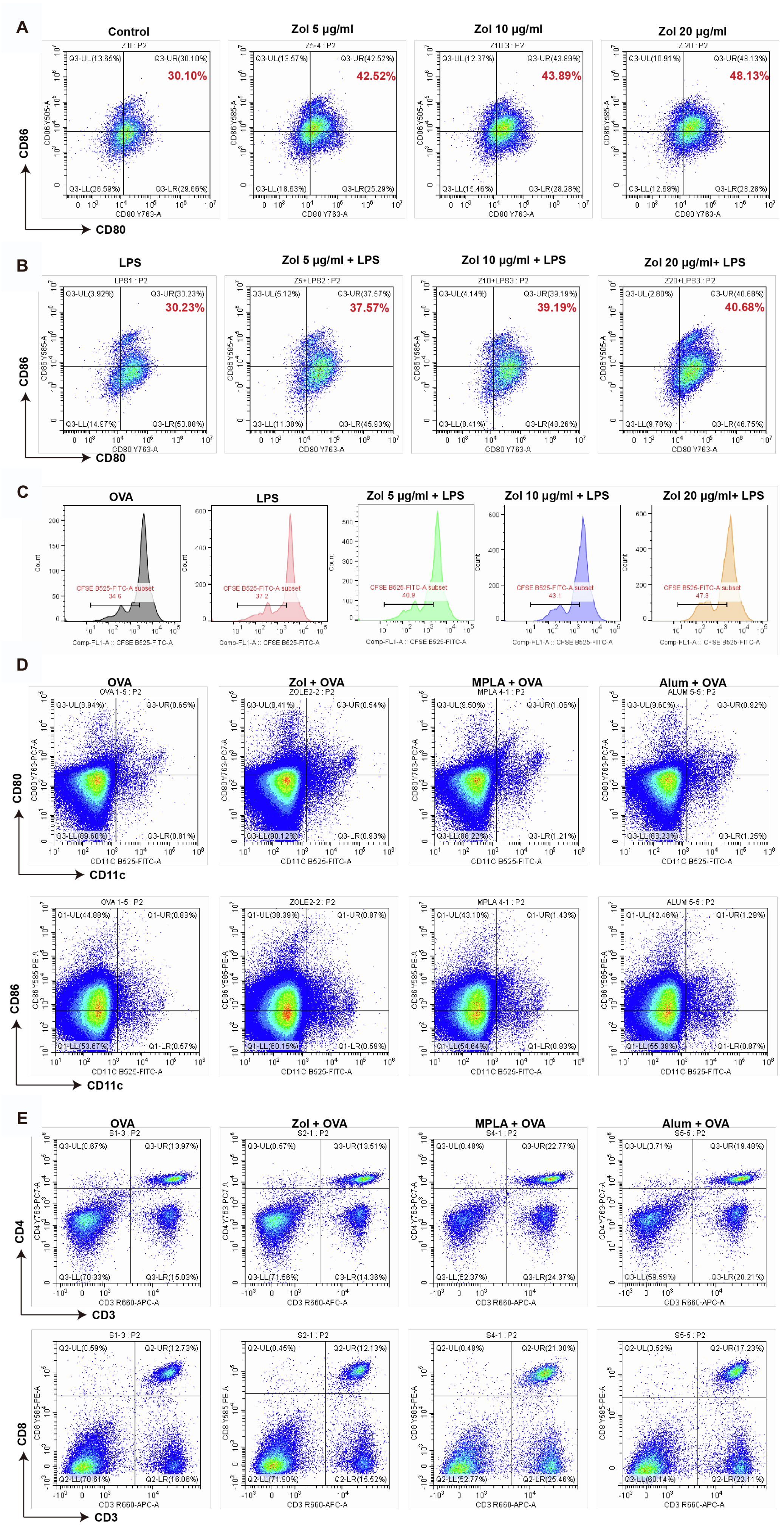
A
Control
Zol 5 μg/ml
Zol 10 μg/ml
Zol 20 μg/ml
30.10%
42.52%
43.89%
48.13%
CD86
CD80
B
LPS
Zol 5 μg/ml + LPS
Zol 10 μg/ml + LPS
Zol 20 μg/ml+ LPS
30.23%
37.57%
39.19%
40.68%
CD86
CD80
C
OVA
LPS
Zol 5 μg/ml + LPS
Zol 10 μg/ml + LPS
Zol 20 μg/ml+ LPS
D
OVA
Zol + OVA
MPLA + OVA
Alum + OVA
CD80
CD11c
CD86
CD11c
E
OVA
Zol + OVA
MPLA + OVA
Alum + OVA
CD4
CD3
CD8
CD3

Figure S3. Representative flow cytometry plots. Related to Figure 1. (A) Representative scatter plots of $CD80^{+}CD86^{+}$ in $CD11c^{+}$ cells after the stimulation of free zoledronate. **(B)** Representative scatter plots of $CD80^{+}CD86^{+}$ in $CD11c^{+}$ cells after the stimulation of free zoledronate and LPS. **(C)** Representative histogram plots of $CFSE^{low}$ cells in $CD3^{+}$ T cells after different treatments. **(D)** Representative scatter plots of $CD11c^{+}CD80^{+}$ and $CD11c^{+}CD86^{+}$ DCs in DLNs. **(E)** Representative scatter plots of $CD3^{+}CD4^{+}$ and $CD3^{+}CD8^{+}$ T cells in spleens after different treatments.

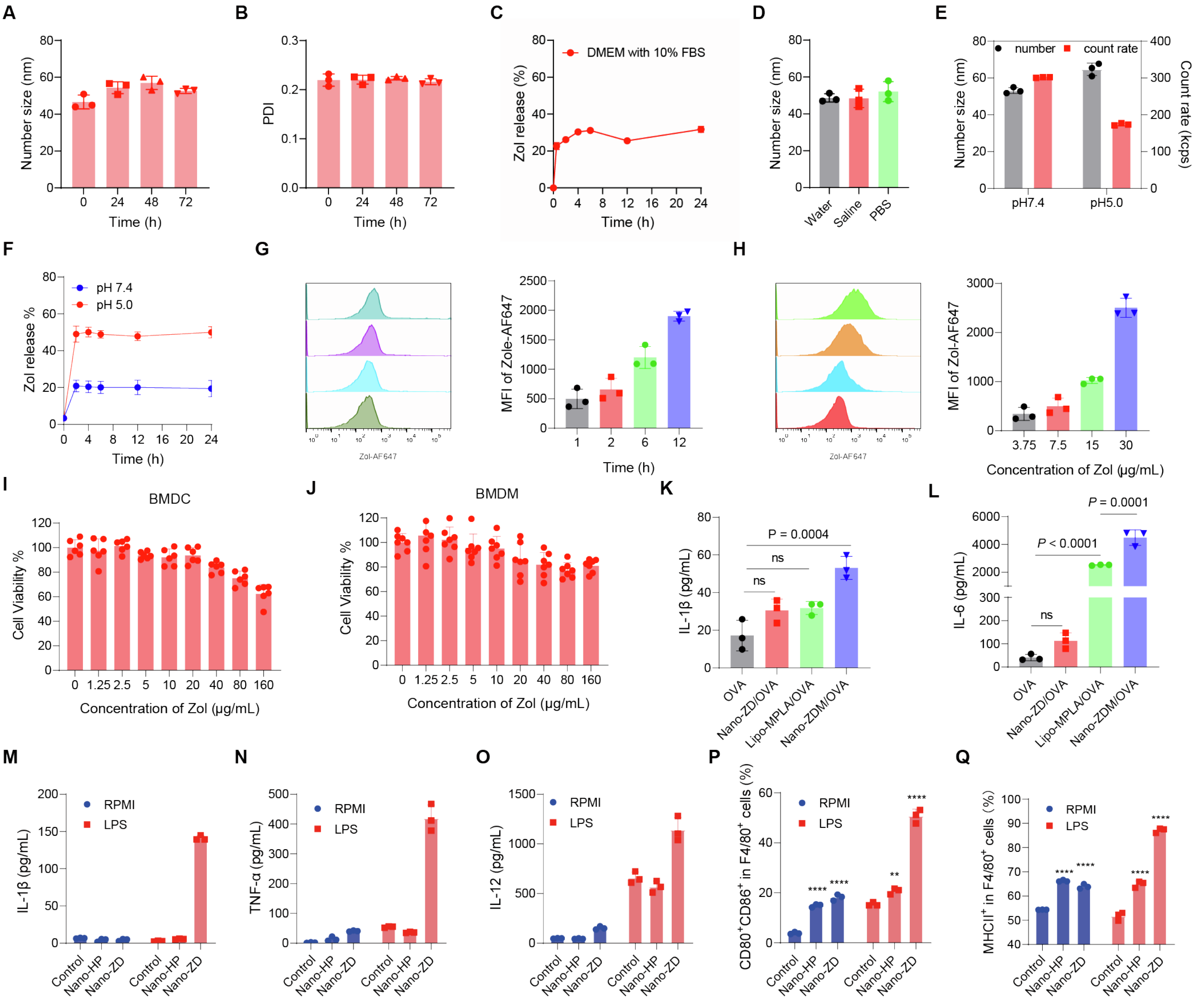

Figure S4. The construction and immune stimulatory effects of Nano-ZD and Nano-ZDM in vitro. Related to Figure 2. (A) Number size of Nano-ZD in DMEM with 10% FBS within 72 h (n = 3). **(B)** Polydispersity index (PDI) of Nano-ZD in DMEM with 10% FBS within 72 h (n = 3). **(C)** Zoledronate release profiles of Nano-ZD in DMEM with 10% FBS (n = 3). **(D)** Number size of Nano-ZD in water, saline or PBS after 24 h incubation (n = 3). **(E)** The particle sizes and count rate of Nano-ZD at different pH measured by DLS (n = 3). **(F)** Zoledronate release profiles of Nano-ZD in different conditions (n = 3). **(G-H)** Time-dependent and concentration-dependent uptake of Nano-ZD/Zol-AF647 on DC2.4 cells (n = 3)**. (I-J)** Cell viability of BMDCs **(I)** and BMDMs **(J)** after the treatments of different concentrations of Nano-ZD for 24 h (n = 6). **(K-L)** ELISA detections of IL-1β **(K)** and IL-6 **(L)** in the supernatant of BMDCs after the treatments of Nano-ZD/OVA or Nano-ZDM/OVA (n = 3). (**M-O)** ELISA detections of IL-1β **(M)**, TNF-α **(N)** and IL-12 **(O)** in the supernatant of BMDMs after the treatments of Nano-ZD alone or restimulation with LPS (n = 3) **(P-Q)** Percentage of $CD80^{+}CD86^{+}$ **(P)** and $MHCII^{+}$ **(Q)** cells in BMDMs after different treatments measured by flow cytometry (n = 3). Data are shown as mean ± s.d.

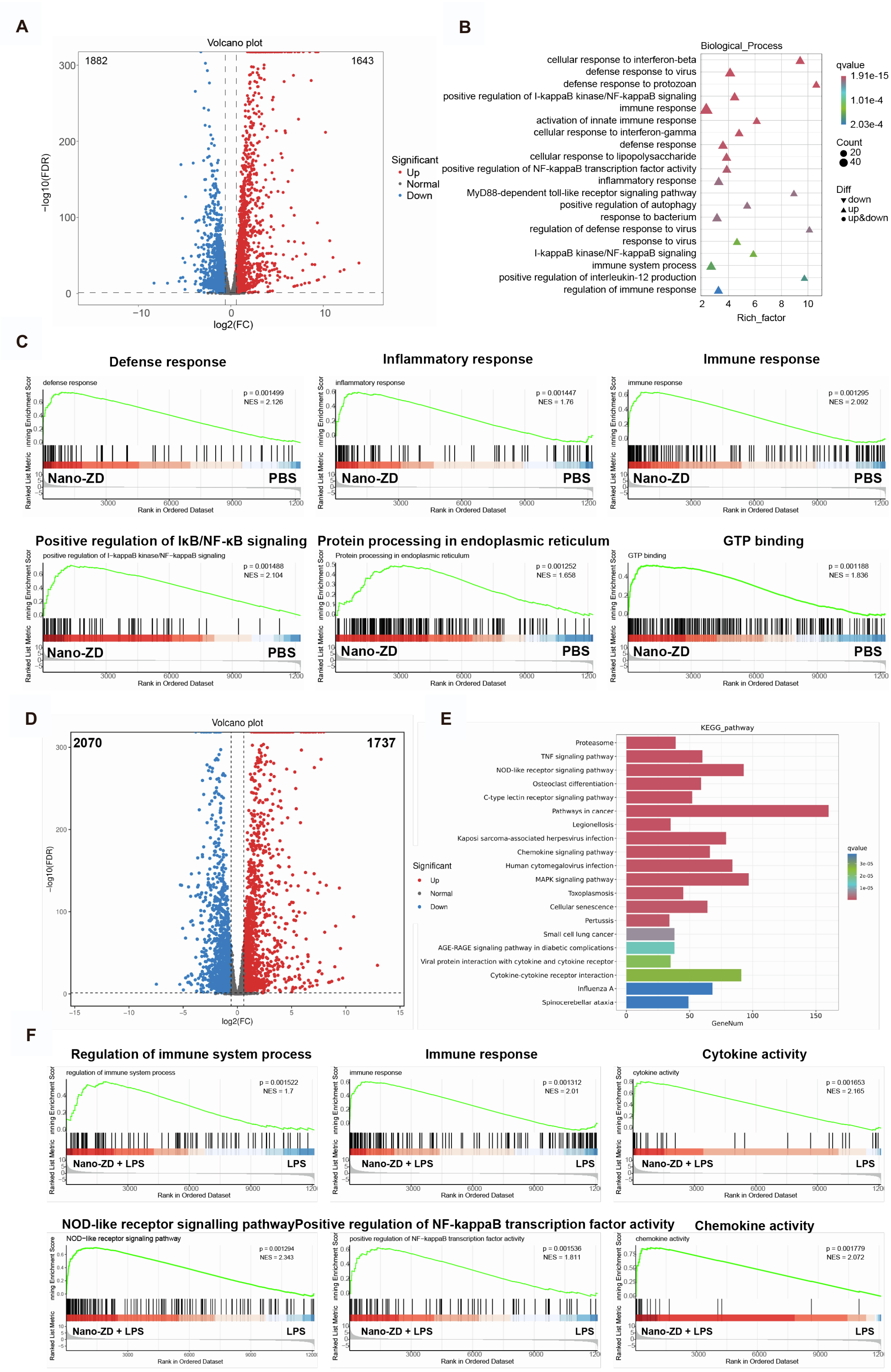

Figure S5. RNA-seq analysis of BMDCs after different treatments. Related to Figure 2. (A-C) The control group is PBS, the experimental group is Nano-ZD. (n = 4) **(A)** Volcano plots of DEGs between PBS and Nano-ZD groups. **(B)** GO enrichment analysis of the upregulated genes in Nano-ZD group. **(C)** GSEA analysis of Nano-ZD group compared with PBS group. **(D-F)** The control group is LPS, the experimental group is Nano-ZD + LPS. (n = 4) **(D)** Volcano plots of DEGs. **(E)** KEGG enrichment pathway analysis of the upregulated genes in Nano-ZD +LPS group. **(F)** Transcript Gene set enrichment analysis (GSEA) of Nano-ZD + LPS group compared with LPS group.

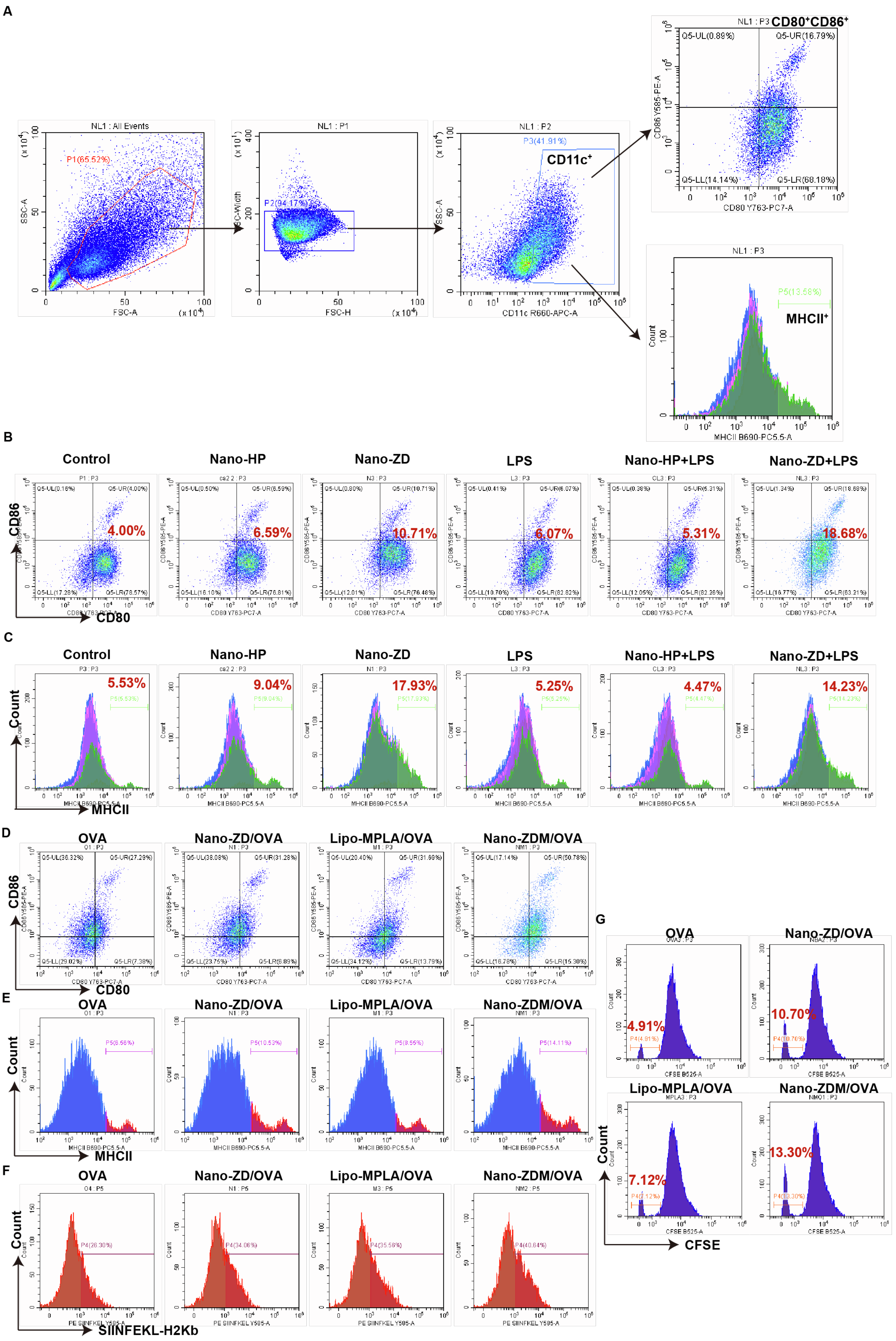
A
CD80+CD86+
CD11c+
MHCII+
B
Control
Nano-HP
Nano-ZD
LPS
Nano-HP+LPS
Nano-ZD+LPS
CD86
CD80
4.00%
6.59%
10.71%
6.07%
5.31%
18.68%
C
Control
Nano-HP
Nano-ZD
LPS
Nano-HP+LPS
Nano-ZD+LPS
Count
MHCII
5.53%
9.04%
17.93%
5.25%
4.47%
14.23%
D
OVA
Nano-ZD/OVA
Lipo-MPLA/OVA
Nano-ZDM/OVA
CD86
CD80
E
OVA
Nano-ZD/OVA
Lipo-MPLA/OVA
Nano-ZDM/OVA
Count
MHCII
F
OVA
Nano-ZD/OVA
Lipo-MPLA/OVA
Nano-ZDM/OVA
Count
SIINFEKL-H2Kb
G
OVA
Nano-ZD/OVA
Lipo-MPLA/OVA
Nano-ZDM/OVA
4.91%
10.70%
7.12%
13.30%
Count
CFSE

Figure S6. (A) Gating Strategies of CD80$^+$CD86$^+$ and MHCII$^+$ cells in BMDCs. Related to Figures 2I-2J and 2L-2M. **(B-C)** Representative scatter plots or histogram plots of CD80$^+$CD86$^+$ **(B)** and MHCII$^+$ **(C)** cells in CD11c$^+$ BMDCs after the stimulation of Nano-ZD or Nano-ZD + LPS. Related to Figures 2I-2J and 2L-2M. **(D-F)** Representative scatter plots or histogram plots of CD80$^+$CD86$^+$ **(D)**, MHCII$^+$ **(E)** and SIINFEKL-H2Kb **(F)** cells in CD11c$^+$ BMDCs after different treatments. Related to Figures 2Q-2S. **(G)** Representative histogram plots of CFSE^{low} cells in CD3$^+$ T cells after different treatments. Related to Figures 2U. Data are shown as mean ± s.d.

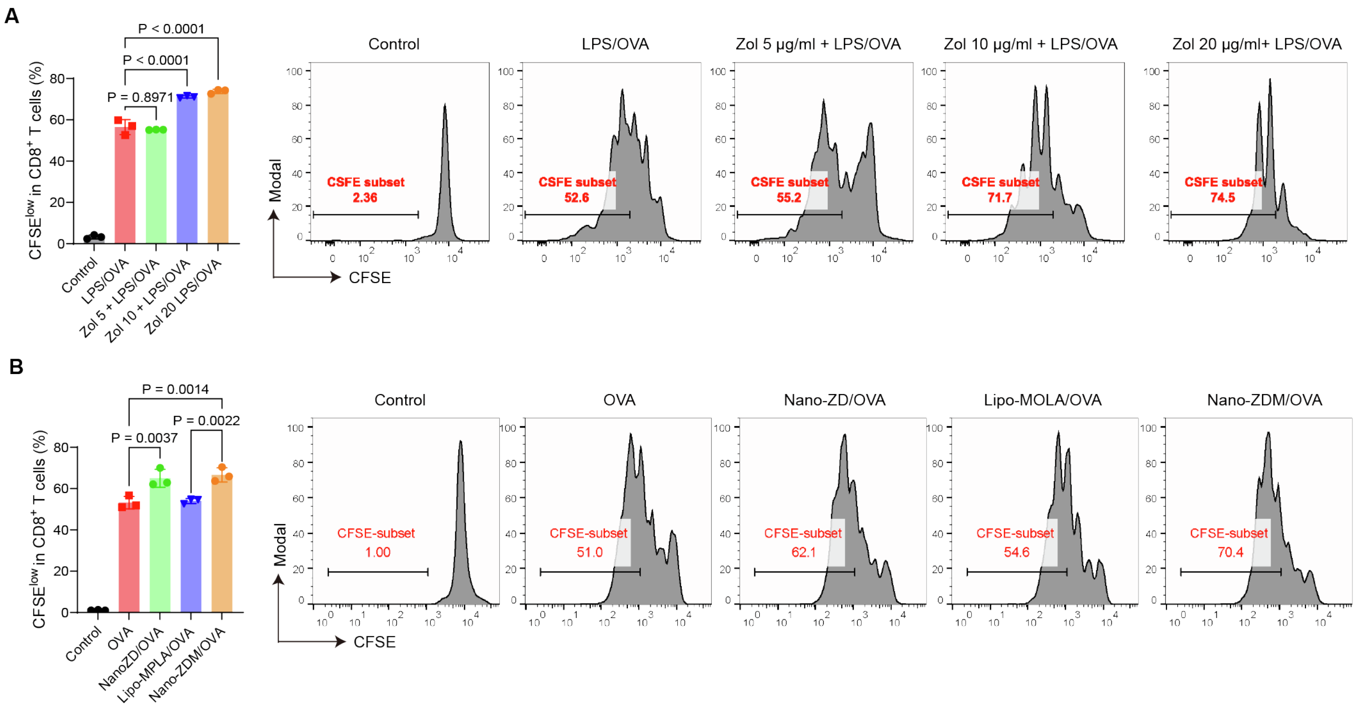

Figure S7. (A) Percentage of CFSE^{low} in CD8$^+$ T cells from OT-1 mice after co-culture with BMDCs pre-treated with free zoledronate (n = 3). **(B)** Percentage of CFSE^{low} in CD8$^+$ T cells from OT-1 mice after co-culture with BMDCs treated with Nano-ZD/OVA or Nano-ZDM/OVA (n = 3). Related to Figure 2. Data are shown as mean ± s.d.

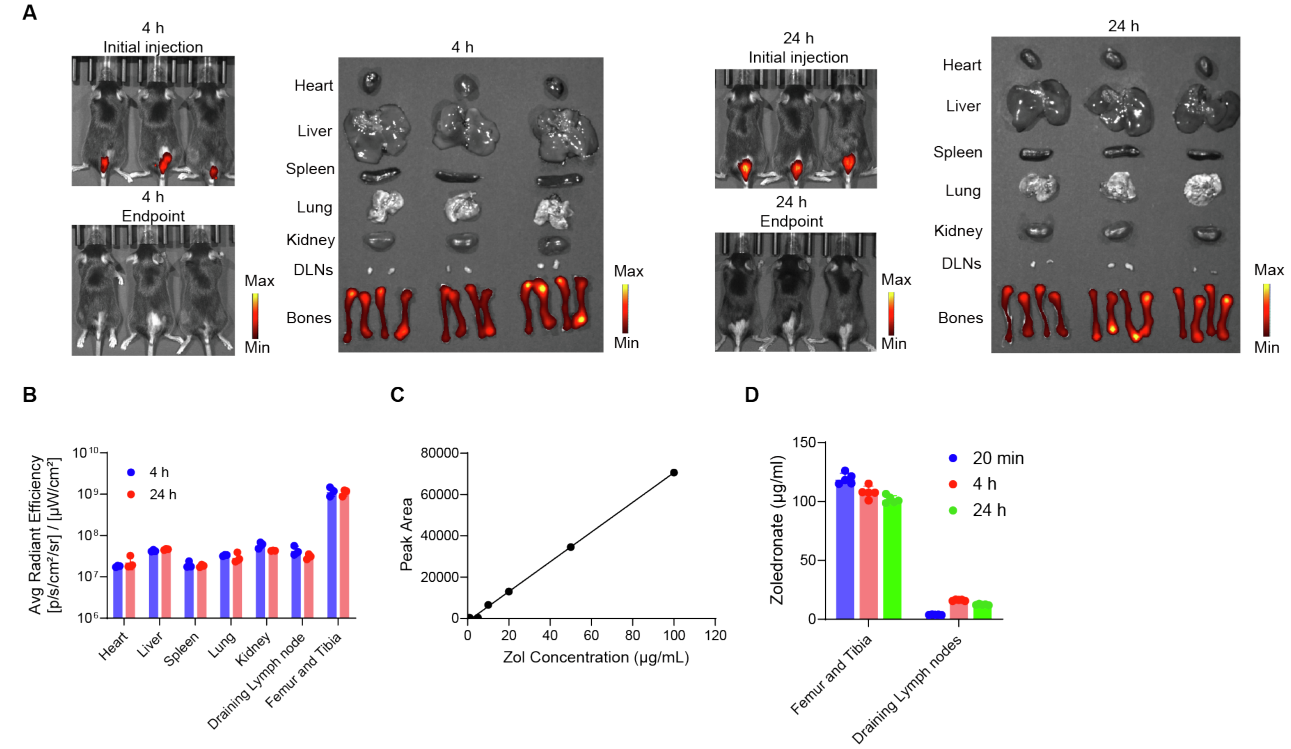

Figure S8. (A-B) Representative fluorescence images and statistical analysis of the distribution intensity of free zoledronate in different tissues after subcutaneous injection of Zol-AF647 for 4 hours and 24 hours (n=3). **(C)** HPLC standard curve of zoledronate in mobile phase. **(D)** Quantitative analysis of zoledronate in the bones and DLNs at different time points after subcutaneous injection of free zoledronate (200 μg per mouse, n=5). Related to Figure 3. Data are shown as mean ± s.d.

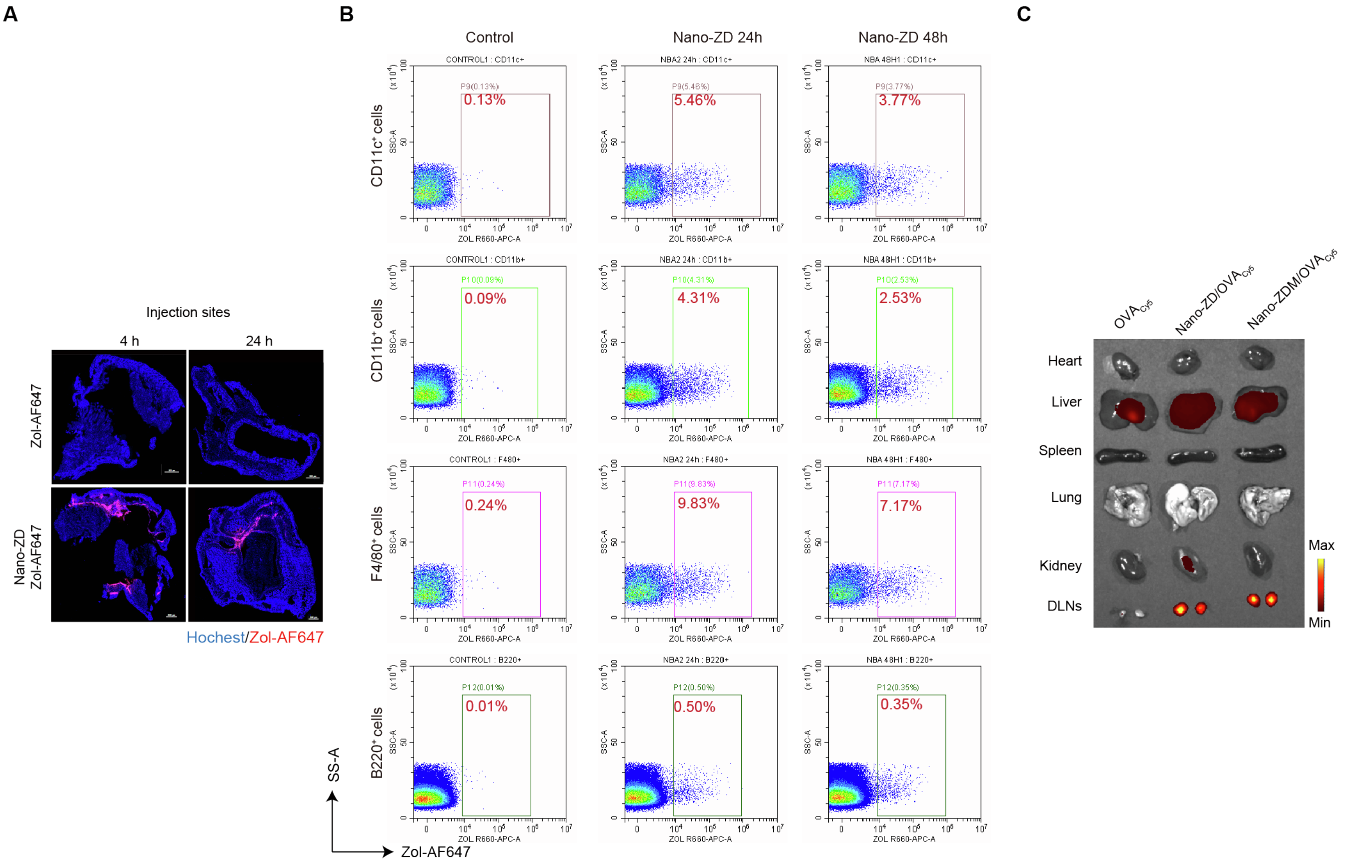

Figure S9. (A) Representative fluorescence images of the section of inject sites after the treatments of Zol-AF647 or Nano-ZD/Zol-AF647 at 4 h and 24 h, scale bar = 800 μm. Related to Figure 3G. **(B)** Representative scatter plots of Zol-AF647^{+} cells in CD11c^{+}, CD11b^{+}, F4/80^{+} or B220^{+} cells. Related to Figure 3H. **(C)** Representative fluorescence images of the DLNs and major organs after the treatments of OVA_{Cy5}, Nano-ZD/OVA_{Cy5}, or Nano-ZDM/OVA_{Cy5} at 24 h, respectively. Related to Figure 3I.

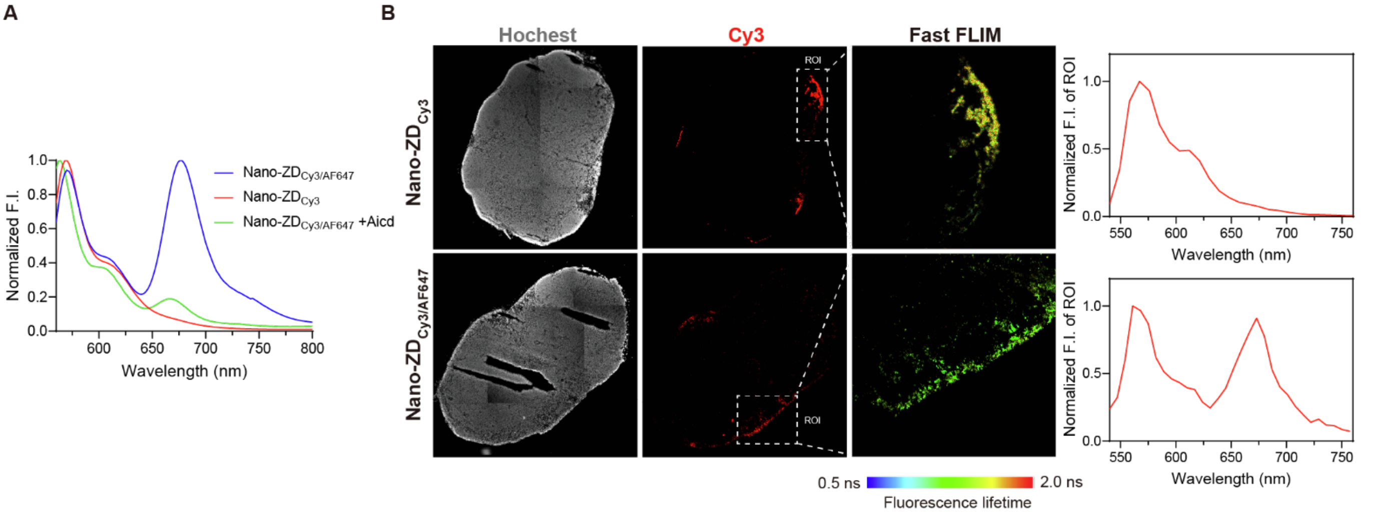

Figure S10. (A) Representative fluorescence emission spectra of Nano-ZD under different conditions upon excitation at 540 nm. **(B)** Representative fluorescence images, fast FLIM images, and local fluorescence emission spectra of draining lymph node sections following injection of Nano-ZD_{Cy3} or Nano-$ZD_{Cy3/AF647}$. Related to Figure 3.

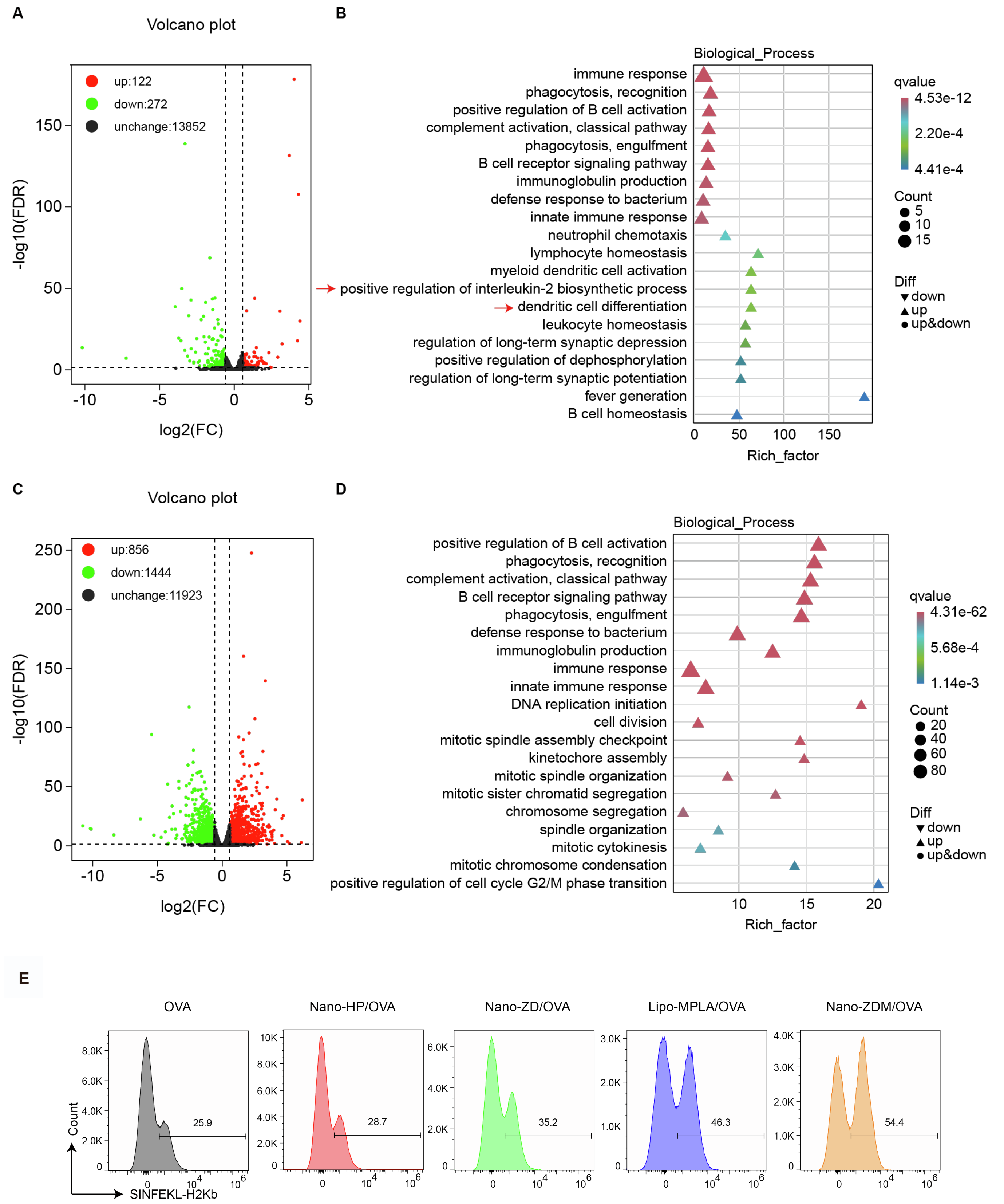

Figure S11. Nano-ZD and Nano-ZDM induce immune activation in DLNs. Related to Figure 3. (A-D) RNA-seq analysis of draining lymph nodes (DLNs) after different treatments. (n = 3) **(A)** Volcano plots of DEGs between Nano-ZD-treated mice and untreated mice. **(B)** GO enrichment analysis of the upregulated genes in DLNs from Nano-ZD-treated mice compared with untreated mice. **(C)** Volcano plots of DEGs between Nano-ZDM-treated mice and untreated mice. **(D)** GO enrichment analysis of the upregulated genes in DLNs from Nano-ZDM-treated mice compared with untreated mice. **(E)** Representative flow cytometry profiles of SIINFEKL-H-2Kb^{+} cells from CD11c^{+} cells in draining lymph nodes. Related to Figure 3O.

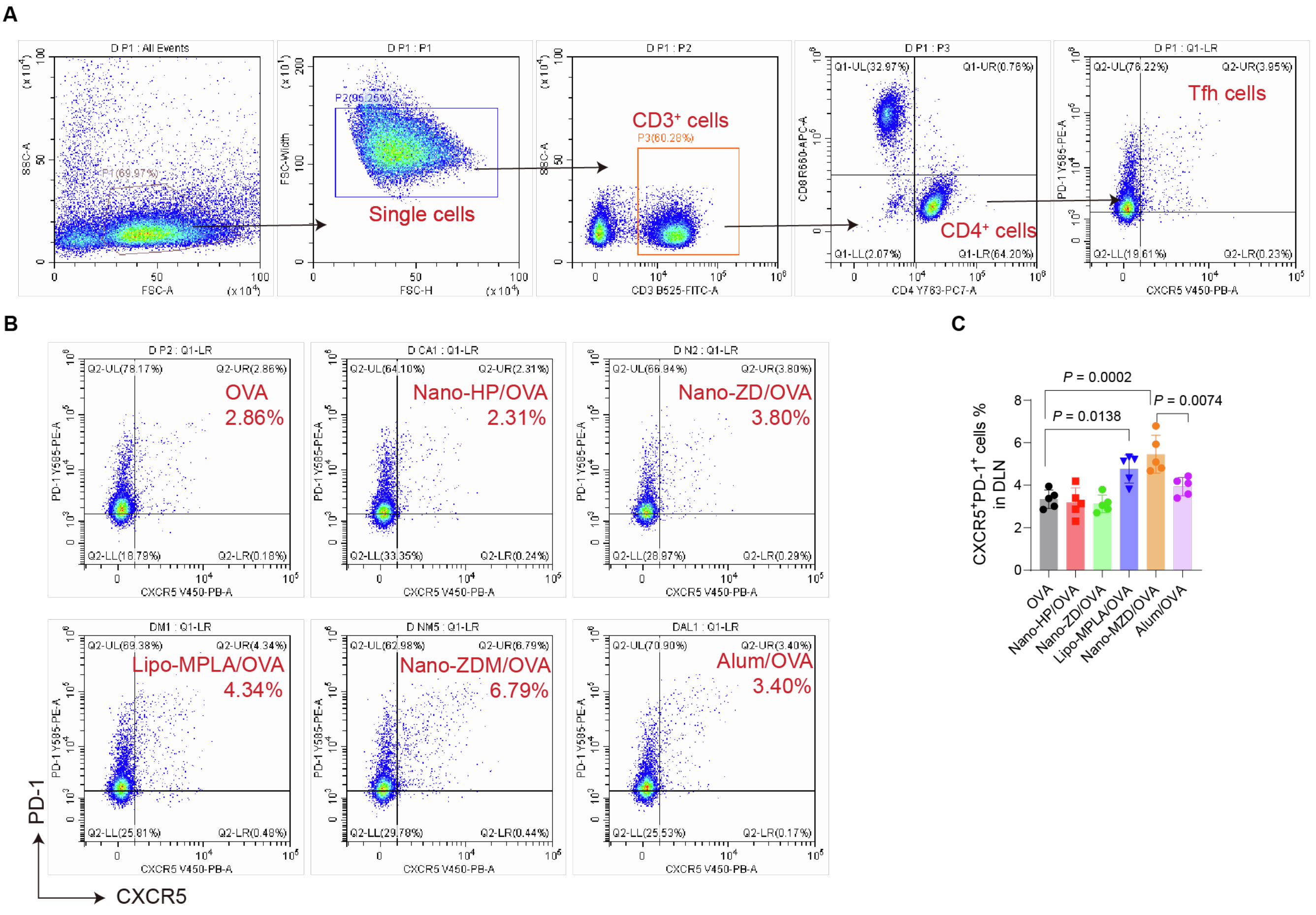

Figure S12. (A) Gating Strategies of Tfh cells in DLNs. **(B-C)** Representative scatter plots and quantification of Tfh cells in DLNs (n = 5). Related to Figure 4. Data are shown as mean ± s.d.

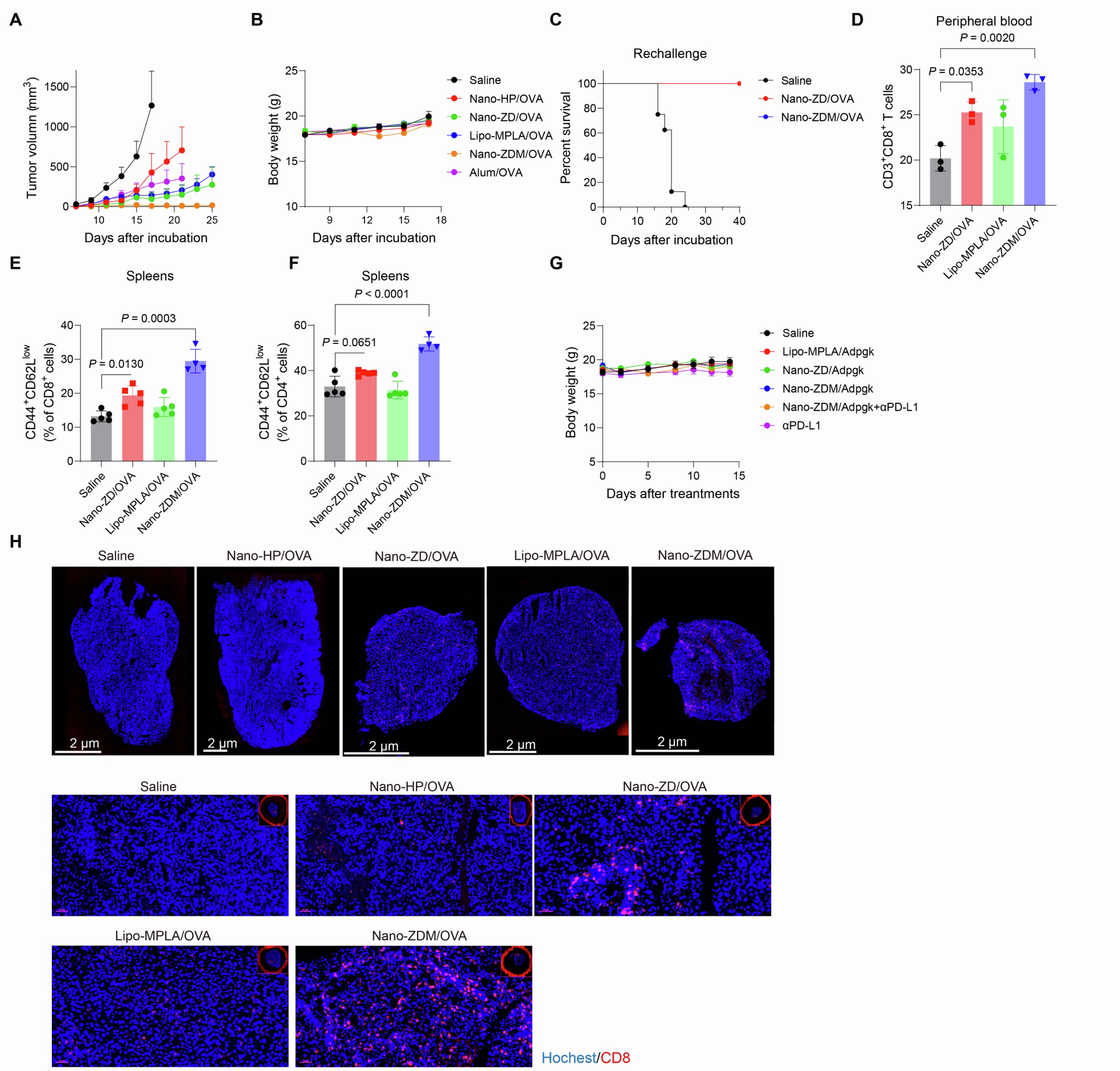

Figure S13. Nano-granulated zoledronate effectively activates the cellular immune response for cancer immunotherapy. Related to Figure 5. (A-B) Tumor growth profiles and body weight changes of B16-OVA tumor-bearing mice during the treatments (n = 8). **(C)** Survival curves of mice with B16-OVA tumor rechallenge on day 60 (n = 8). **(D)** The percentage of $CD3^+CD8^+$ T cells in peripheral blood from mice with different treatments (n = 3). **(E-F)** The percentage of effector memory T cells among $CD8^+$ T cells and $CD4^+$ T cells in spleens after different treatments (n = 4-5). **(G)** Body weight changes of MC38 tumor-bearing mice during the treatments (n = 7-8). **(H)** Immunofluorescence staining of $CD8^+$ T cells of tumor sections from B16-OVA-bearing mice with different treatments. Data are shown as mean ± s.d.

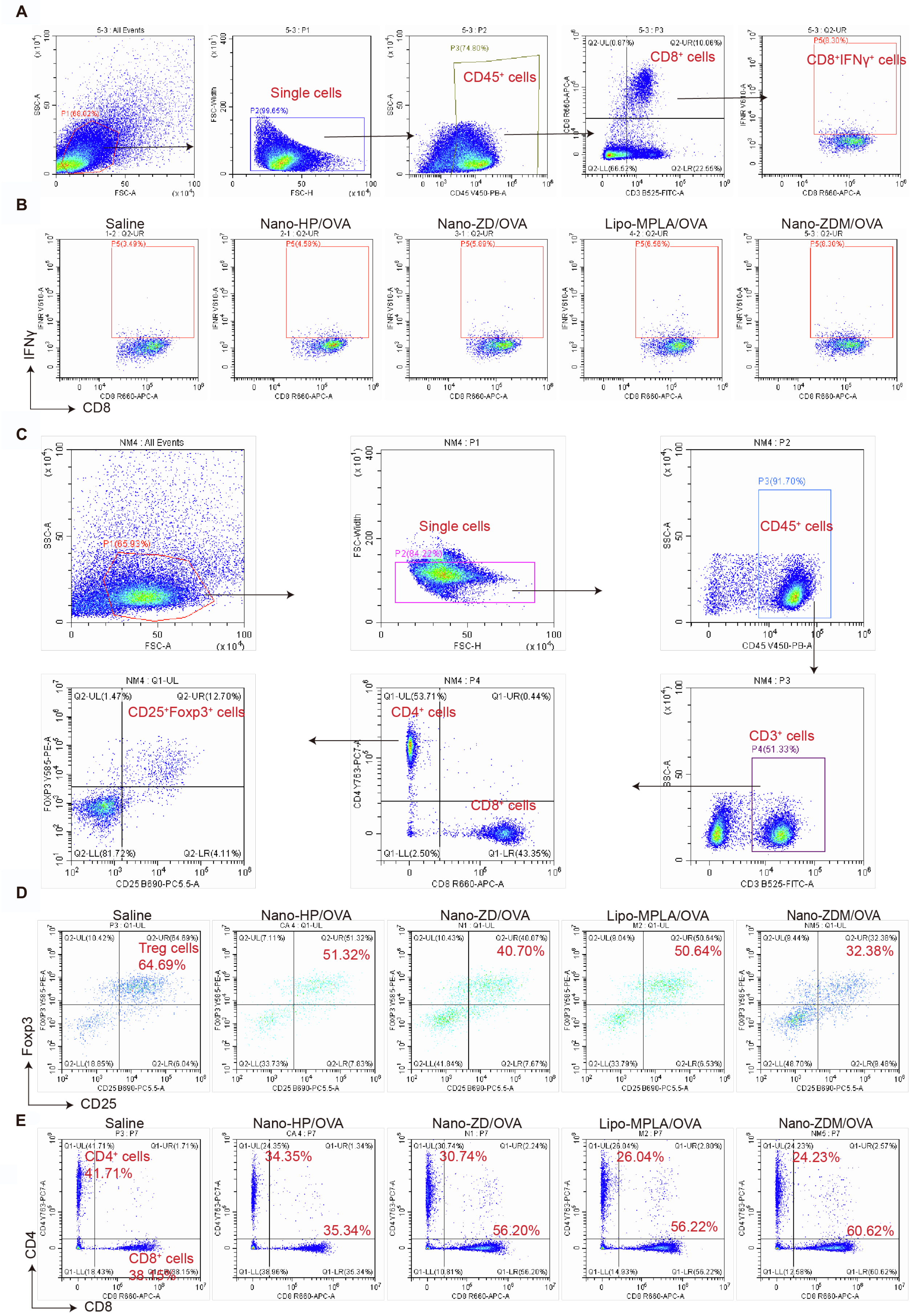

Figure S14. (A-B) Gating Strategies (A) and representative scatter plots (B) of CD8$^+$IFNγ$^+$ T cells in spleens. Related to Figure 5G. **(C)** Gating Strategies of Treg (CD25$^+$Foxp3$^+$) cells, CD8$^+$ T cells and CD4$^+$ T cells (spleen cells). **(D)** Representative scatter plots of Treg cells in tumors after different treatments. **(E)** Representative scatter plots of CD8$^+$ T cells and CD4$^+$ T cells in tumors after different treatments. Related to Figures 5H and 5I.

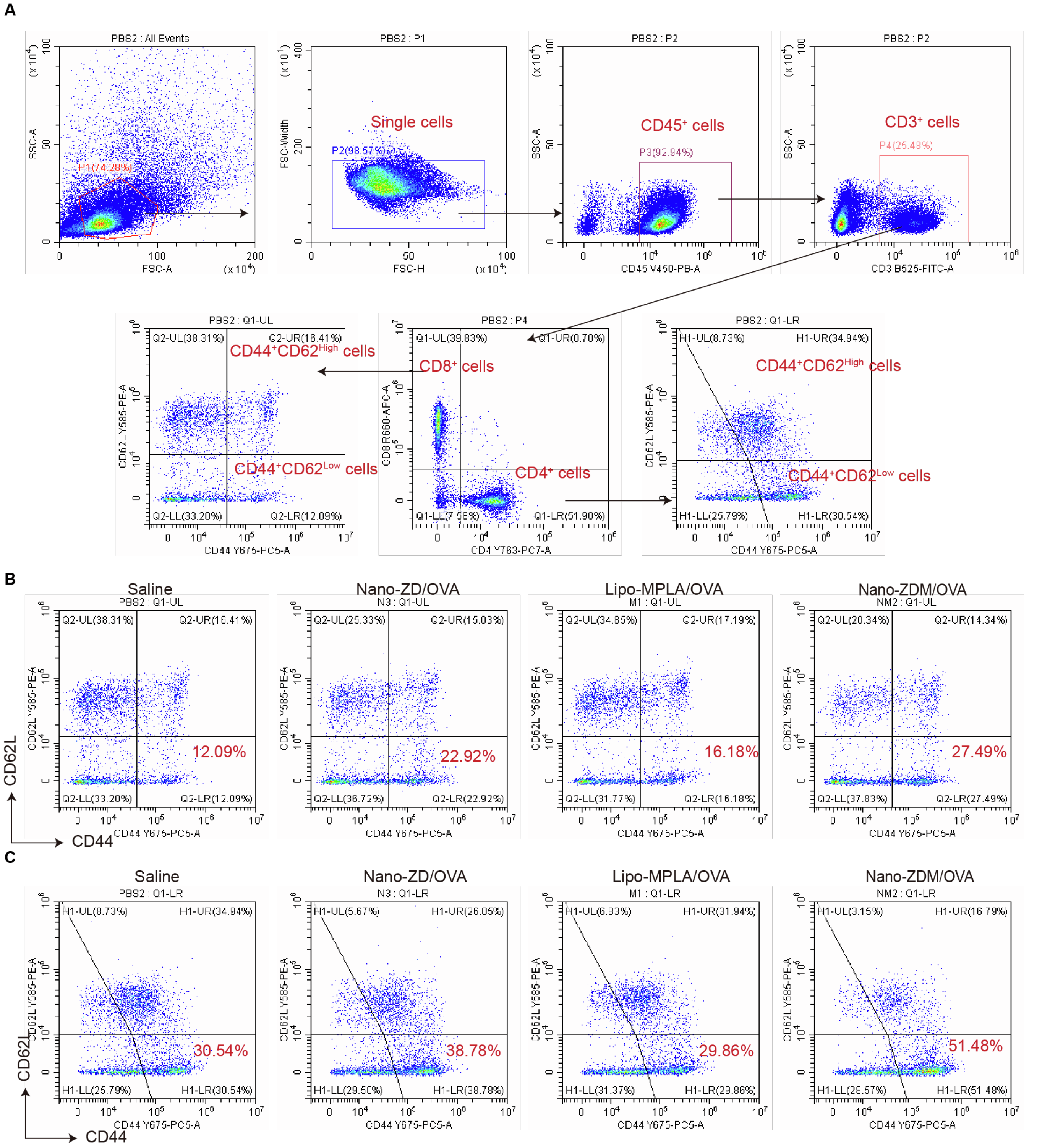

Figure S15. Gating Strategies **(A)** and representative scatter plots of effector memory T cells ($CD44^{+}CD62^{low}$, Tem) in $CD8^{+}$ **(B)** or $CD4^{+}$ T cells **(C)**. Related to Figures S13E- S13F.

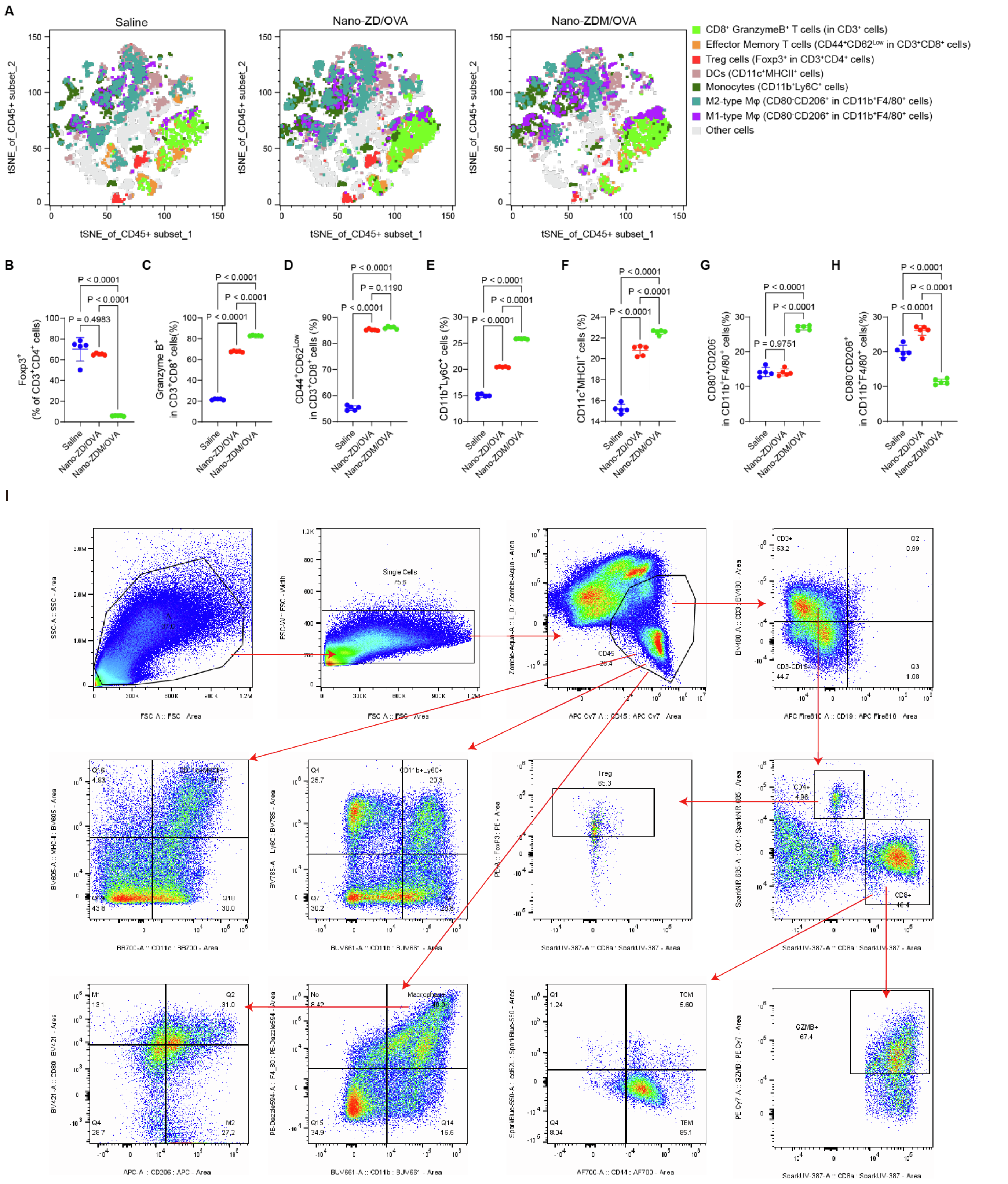

Figure S16. Full-spectrum flow cytometry analysis of tumor tissues. Related to Figure 5. (A) Representative t-SNE plots of tumor-infiltrating immune cell subpopulation after different treatments. **(B-H)** Percentage of tumor-infiltrating Treg cells **(B)**, $CD8^{+}$ Granzyme B^{+} T cells **(C)**, Effect memory T cells **(D)**, Monocytes **(E)**, DCs **(F)**, M1-type macrophages **(G)**and M2-type macrophages **(H)** after different treatments (n = 5). **(I)** Gating Strategies of full-spectrum flow cytometry analysis. Data are shown as mean ± s.d.

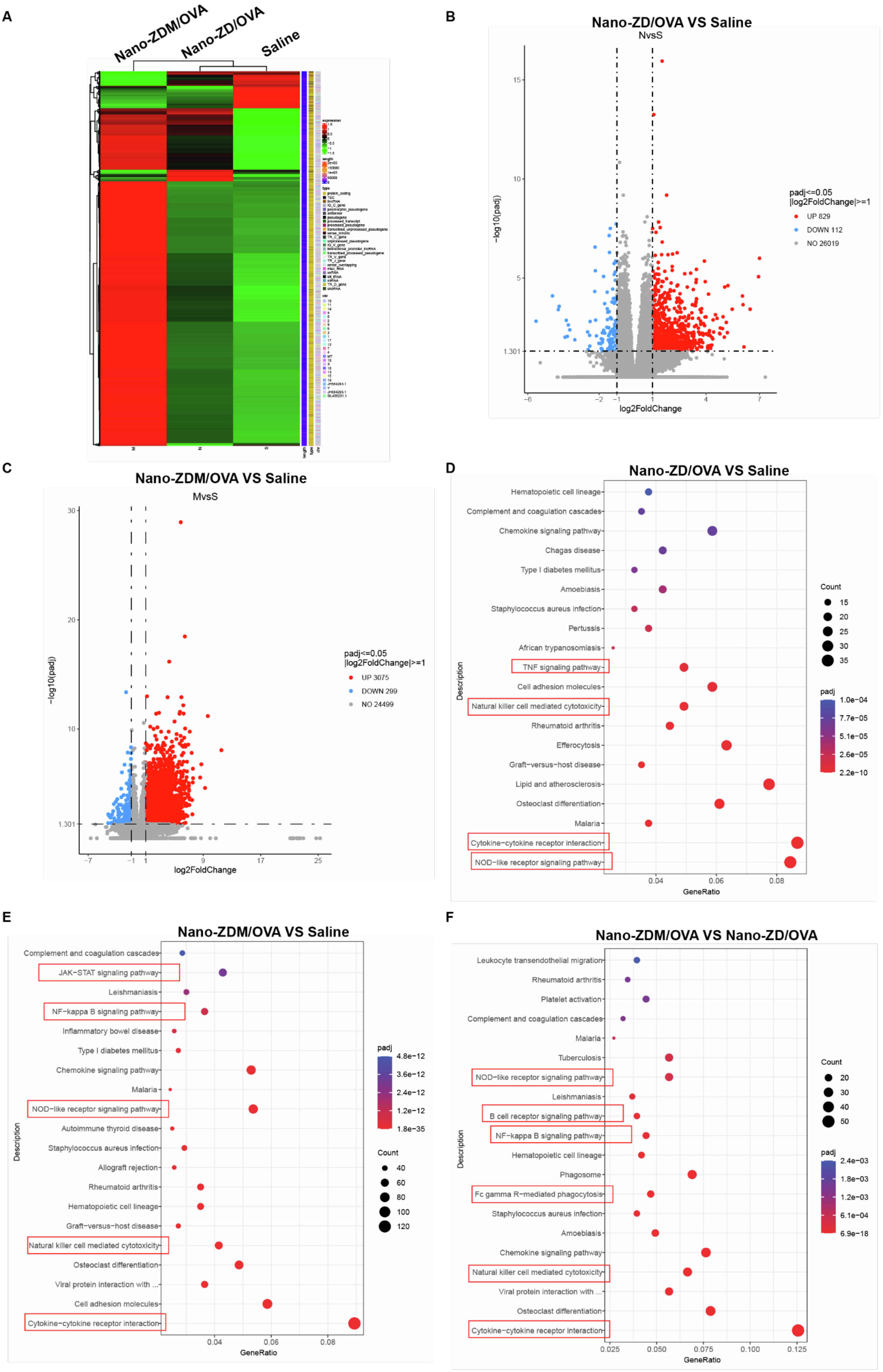
A
Nano-ZDM/OVA
Nano-ZD/OVA
Saline
B
Nano-ZD/OVA VS Saline
NvsS
−log10(padj)
log2FoldChange
padj<=0.05
|log2FoldChange|>=1
UP 829
DOWN 112
NO 26019
C
Nano-ZDM/OVA VS Saline
MvsS
−log10(padj)
log2FoldChange
padj<=0.05
|log2FoldChange|>=1
UP 3075
DOWN 299
NO 24499
D
Nano-ZD/OVA VS Saline
Hematopoietic cell lineage
Complement and coagulation cascades
Chemokine signaling pathway
Chagas disease
Type I diabetes mellitus
Amoebiasis
Staphylococcus aureus infection
Pertussis
African trypanosomiasis
TNF signaling pathway
Cell adhesion molecules
Natural killer cell mediated cytotoxicity
Rheumatoid arthritis
Efferocytosis
Graft−versus−host disease
Lipid and atherosclerosis
Osteoclast differentiation
Malaria
Cytokine−cytokine receptor interaction
NOD−like receptor signaling pathway
Description
GeneRatio
Count
padj
E
Nano-ZDM/OVA VS Saline
Complement and coagulation cascades
JAK−STAT signaling pathway
Leishmaniasis
NF−kappa B signaling pathway
Inflammatory bowel disease
Type I diabetes mellitus
Chemokine signaling pathway
Malaria
NOD−like receptor signaling pathway
Autoimmune thyroid disease
Staphylococcus aureus infection
Allograft rejection
Rheumatoid arthritis
Hematopoietic cell lineage
Graft−versus−host disease
Natural killer cell mediated cytotoxicity
Osteoclast differentiation
Viral protein interaction with ...
Cell adhesion molecules
Cytokine−cytokine receptor interaction
Description
GeneRatio
padj
Count
F
Nano-ZDM/OVA VS Nano-ZD/OVA
Leukocyte transendothelial migration
Rheumatoid arthritis
Platelet activation
Complement and coagulation cascades
Malaria
Tuberculosis
NOD−like receptor signaling pathway
Leishmaniasis
B cell receptor signaling pathway
NF−kappa B signaling pathway
Hematopoietic cell lineage
Phagosome
Fc gamma R−mediated phagocytosis
Staphylococcus aureus infection
Amoebiasis
Chemokine signaling pathway
Natural killer cell mediated cytotoxicity
Viral protein interaction with ...
Osteoclast differentiation
Cytokine−cytokine receptor interaction
Description
GeneRatio
Count
padj

Figure S17. RNA-seq analysis of B16-OVA tumor tissues after the treatments with Saline, Nano-ZD/OVA or Nano-ZDM/OVA (n = 4). Related to Figure 5. (A) Clustering heat map of DEGs. **(B-C)** Volcano plots of DEGs. **(D-F)** KEGG enrichment pathways of upregulated DEGs.

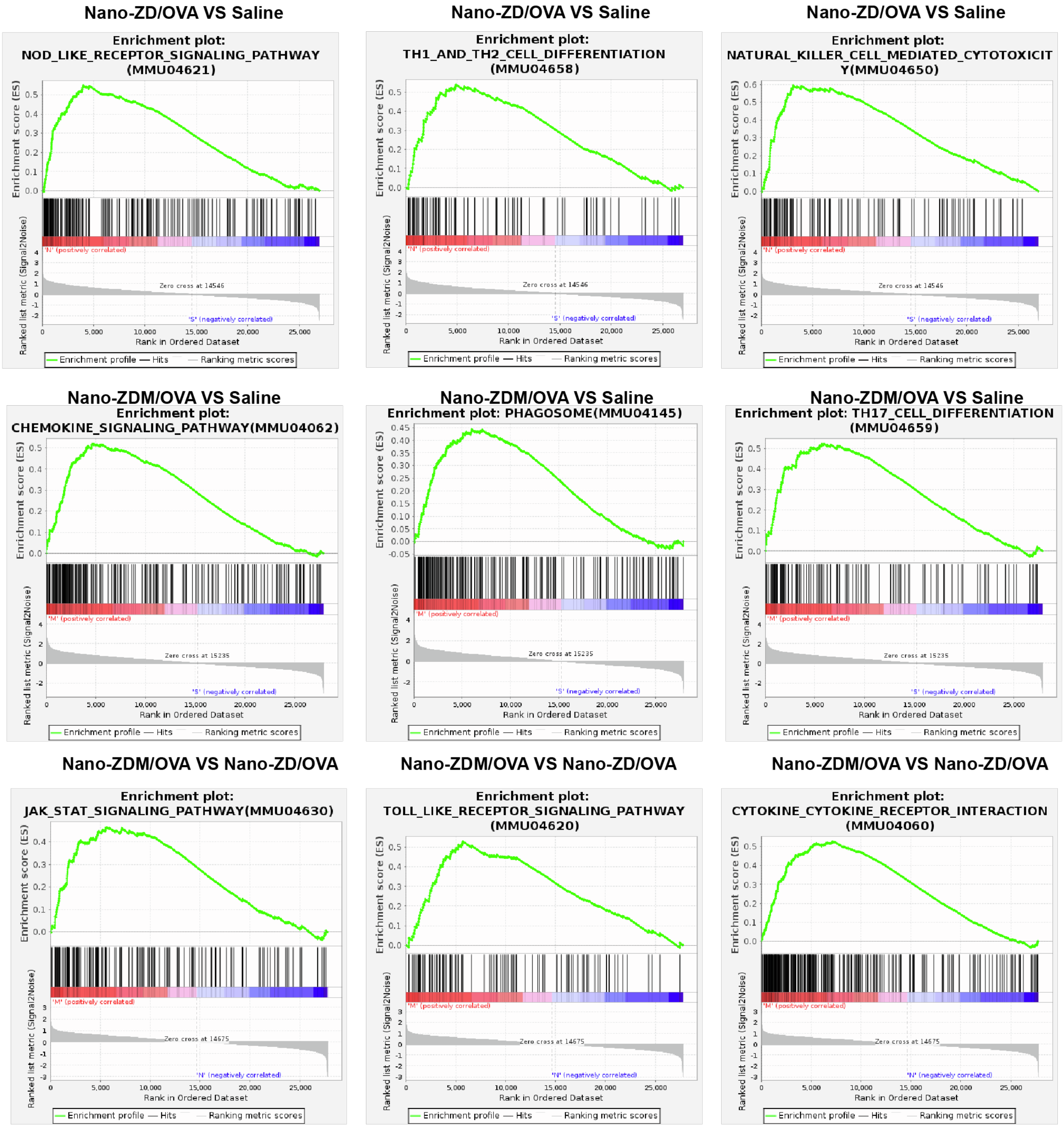

Figure S18. Parts of GSEA enrichment analysis in B16-OVA tumor tissues after the treatments with Saline, Nano-ZD/OVA or Nano-ZDM/OVA.

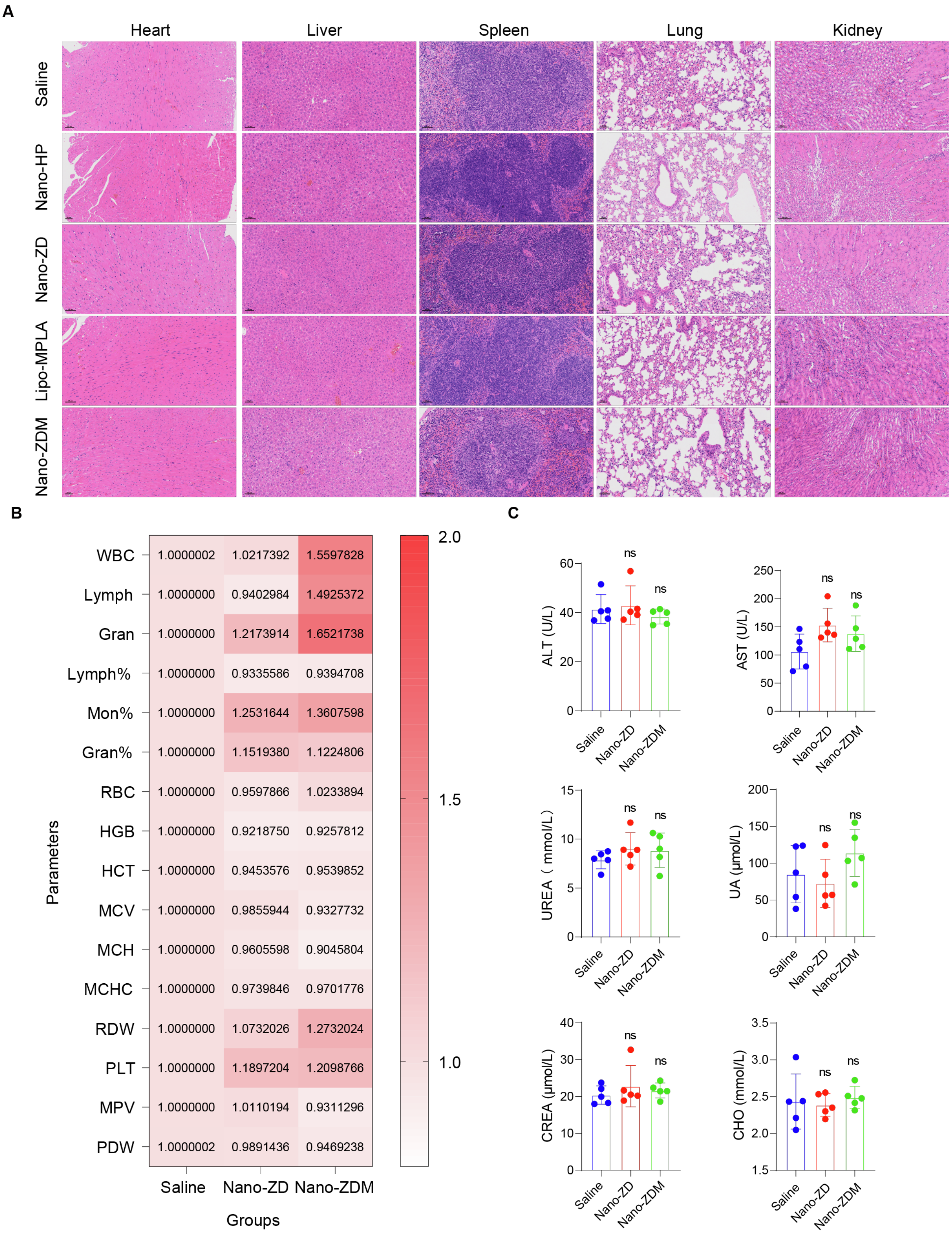

Figure S19. Safety evaluation of nanoparticles. Related to Figure 5. C57BL/6J mice were subcutaneously injected with different treatments every 6 days for 2 times and sacrificed 9 days after the last treatments. (22 μg zoledronate, 5 μg MPLA and 100 μg Alhydrogel per mouse per time). **(A)** H&E staining of major organs. No significant tissue damage was detected after the treatment of Nano-ZD or Nano-ZDM. Scale bar = 50 μm **(B-C)** The blood and sera were collected from different treatments mice for blood routine and blood biochemical test (n = 5). WBC (White Blood Cell), Lymph (Lymphocyte), Gran (Granulocyte), Mon (Monocyte), RBC (Red Blood Cell), HGB (Hemoglobin), HCT (Hematocrit), MCV (Mean Corpuscular Volume), MCH (Mean Corpuscular Hemoglobin), MCHC (Mean Corpuscular Hemoglobin Concentration), RDW (Red Cell Distribution Width), PLT (Platelet Count), MPV (Mean Platelet Volume), PDW (Platelet Distribution Width), ALT (Alanine Aminotransferase), AST (Aspartate Aminotransferase), UREA (Urea), UA (Uric Acid), CREA (Creatinine), CHO (Total Cholesterol). No significant haematological toxicity, hepatotoxicity and nephrotoxicity were detected after the treatment of Nano-ZD or Nano-ZDM. Data are shown as mean ± s.d.

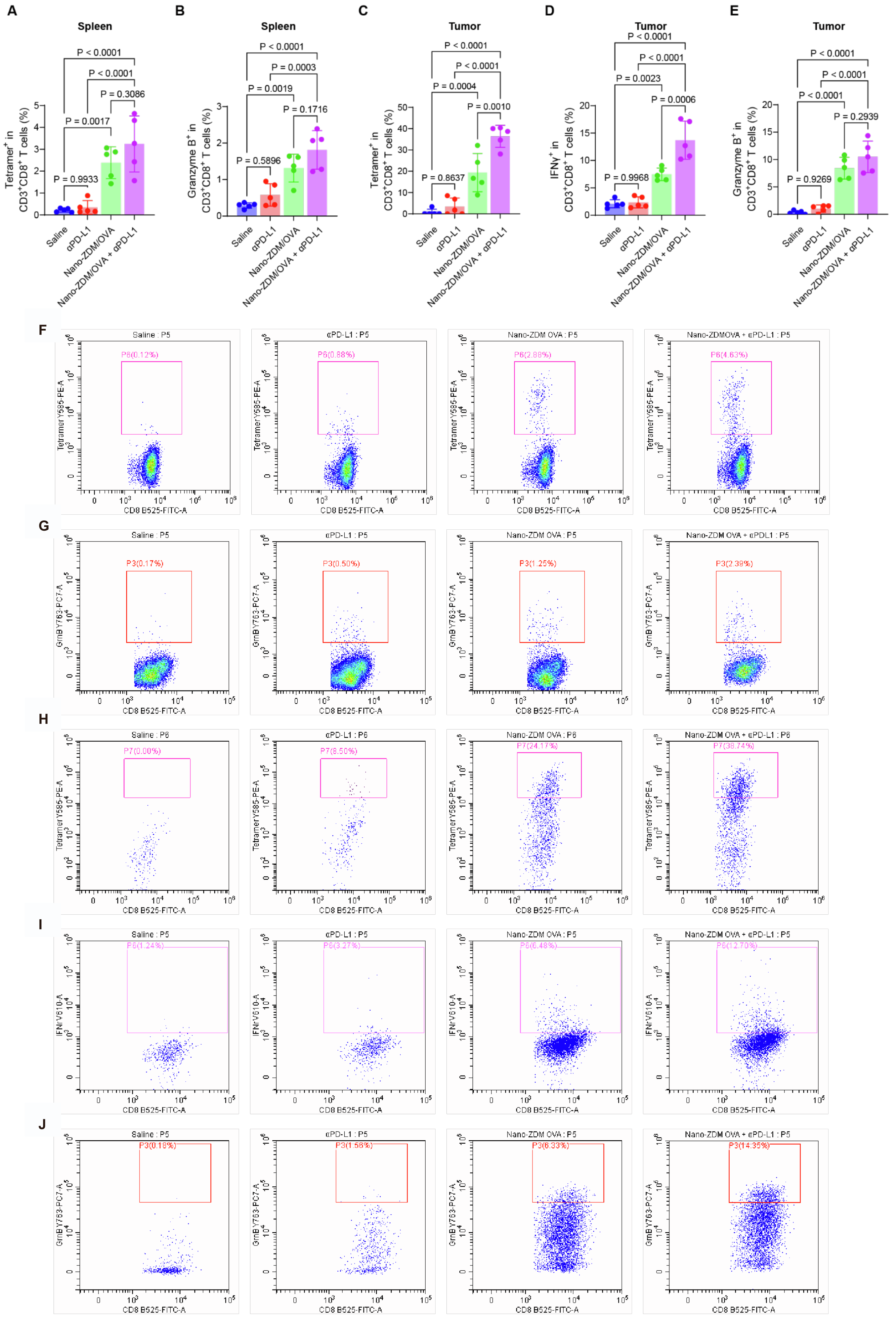
A
Spleen
P < 0.0001
P < 0.0001
P = 0.3086
P = 0.0017
P = 0.9933
Tetramer+ in CD3+CD8+ T cells (%)
B
Spleen
P < 0.0001
P = 0.0003
P = 0.0019
P = 0.1716
P = 0.5896
Granzyme B+ in CD3+CD8+ T cells (%)
C
Tumor
P < 0.0001
P < 0.0001
P = 0.0004
P = 0.0010
P = 0.8637
Tetramer+ in CD3+CD8+ T cells (%)
D
Tumor
P < 0.0001
P < 0.0001
P = 0.0023
P = 0.0006
P = 0.9968
IFNγ+ in CD3+CD8+ T cells (%)
E
Tumor
P < 0.0001
P < 0.0001
P < 0.0001
P = 0.2939
P = 0.9269
Granzyme B+ in CD3+CD8+ T cells (%)
Saline
αPD-L1
Nano-ZDM/OVA
Nano-ZDM/OVA + αPD-L1
F
Saline : P5
P6(0.12%)
αPD-L1 : P5
P6(0.88%)
Nano-ZDM OVA : P5
P6(2.88%)
Nano-ZDMOVA + αPD-L1 : P5
P6(4.63%)
Tetramer Y585-PE-A
CD8 B525-FITC-A
G
Saline : P5
P3(0.17%)
αPD-L1 : P5
P3(0.50%)
Nano-ZDM OVA : P5
P3(1.25%)
Nano-ZDM OVA + αPDL1 : P5
P3(2.39%)
GrnB Y763-PC7-A
CD8 B525-FITC-A
H
Saline : P6
P7(0.00%)
αPD-L1 : P6
P7(8.50%)
Nano-ZDM OVA : P6
P7(24.17%)
Nano-ZDM OVA + αPD-L1 : P6
P7(38.74%)
Tetramer Y585-PE-A
CD8 B525-FITC-A
I
Saline : P5
P6(1.24%)
αPD-L1 : P5
P6(3.27%)
Nano-ZDM OVA : P5
P6(6.48%)
Nano-ZDM OVA + αPD-L1 : P5
P6(12.70%)
IFNr V610-A
CD8 B525-FITC-A
J
Saline : P5
P3(0.18%)
αPD-L1 : P5
P3(1.56%)
Nano-ZDM OVA : P5
P3(6.33%)
Nano-ZDM OVA + αPD-L1 : P5
P3(14.35%)
GrnB Y763-PC7-A
CD8 B525-FITC-A

Figure S20. Flow cytometry analysis of antigen-specific T-cell responses in B16-OVA tumor bearing mice following combination treatment with Nano-ZDM/OVA and αPD-L1 (n = 5). Related to Figure 5. (A) Percentage of OVA-specific tetramer^{+} CD8^{+} T cells in spleens. **(B)** Percentage of Granzyme B^{+} CD8^{+} T cells in spleens **(C)** Percentage of OVA-specific tetramer^{+} CD8^{+} T cells in tumors. **(D)** Percentage of IFNγ^{+} CD8^{+} T cells in tumors. **(E)** Percentage of Granzyme B^{+} CD8^{+} T cells in tumors. **(F)** Representative scatter plots of OVA-specific tetramer^{+} CD8^{+} T cells in spleens. **(G)** Representative scatter plots of Granzyme B^{+} CD8^{+} T cells in spleens. **(H)** Representative scatter plots of OVA-specific tetramer^{+} CD8^{+} T cells in tumors. **(I)** Representative scatter plots of IFNγ^{+} CD8^{+} T cells in tumors. **(J)** Representative scatter plots of Granzyme B^{+} CD8^{+} T cells in tumors. Data are shown as mean ± s.d.

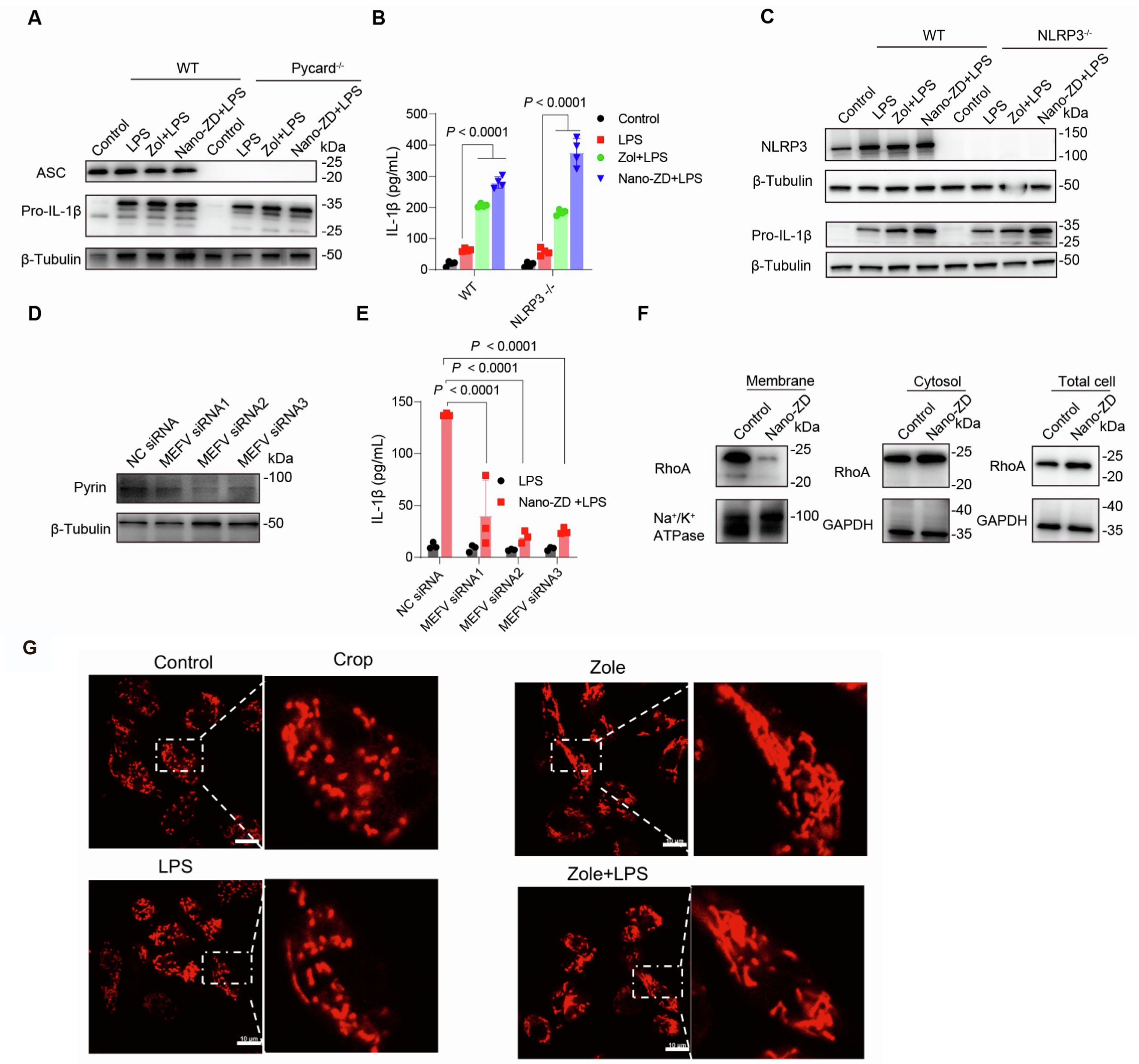

Figure S21. Nano-granulated zoledronate sensitizes and amplifies innate immunity by activating pyrin inflammasome and triggering mtROS-dependent MAVS oligomerization. Related to Figure 6. (A) Western blot analysis of ASC and pro-IL-1β protein expression in the lysates of BMDCs from Pycard$^{-/-}$ mice. Related to Figure 6C. **(B)** ELISA detections of IL-1β in the supernatant of BMDCs from NLRP3$^{-/-}$ mice. **(C)** Western blot analysis of NLRP3 and pro-IL-1β protein expression in the lysates of BMDCs from NLRP3$^{-/-}$ mice. β-tubulin was used as a loading control. **(D)** Western blot analysis of pyrin protein expression in the lysates of BMDCs after the treatments of MEFV siRNA. β-tubulin was used as a loading control. Related to Figure 6D. **(E)** ELISA detections of IL-1β in the supernatant of BMDMs after the treatments of MEFV siRNA (n =3). **(F)** Western blot analysis of the expression of RhoA in membrane, cytosol, and total cell in BMDCs after the treatments of Nano-ZD. Na^{+}/K^{+} ATPase was used as a loading control of membrane proteins. GAPDH was used as a loading control of cytosol proteins. **(G)** Mitochondrial morphology of BMDCs after different treatments. Related to Figure 6I. BMDCs were stained with Mitotracker deep red and analyzed by confocal microscopy. Representative images are shown. Scale bar = 10 μm. Data are shown as mean ± s.d.

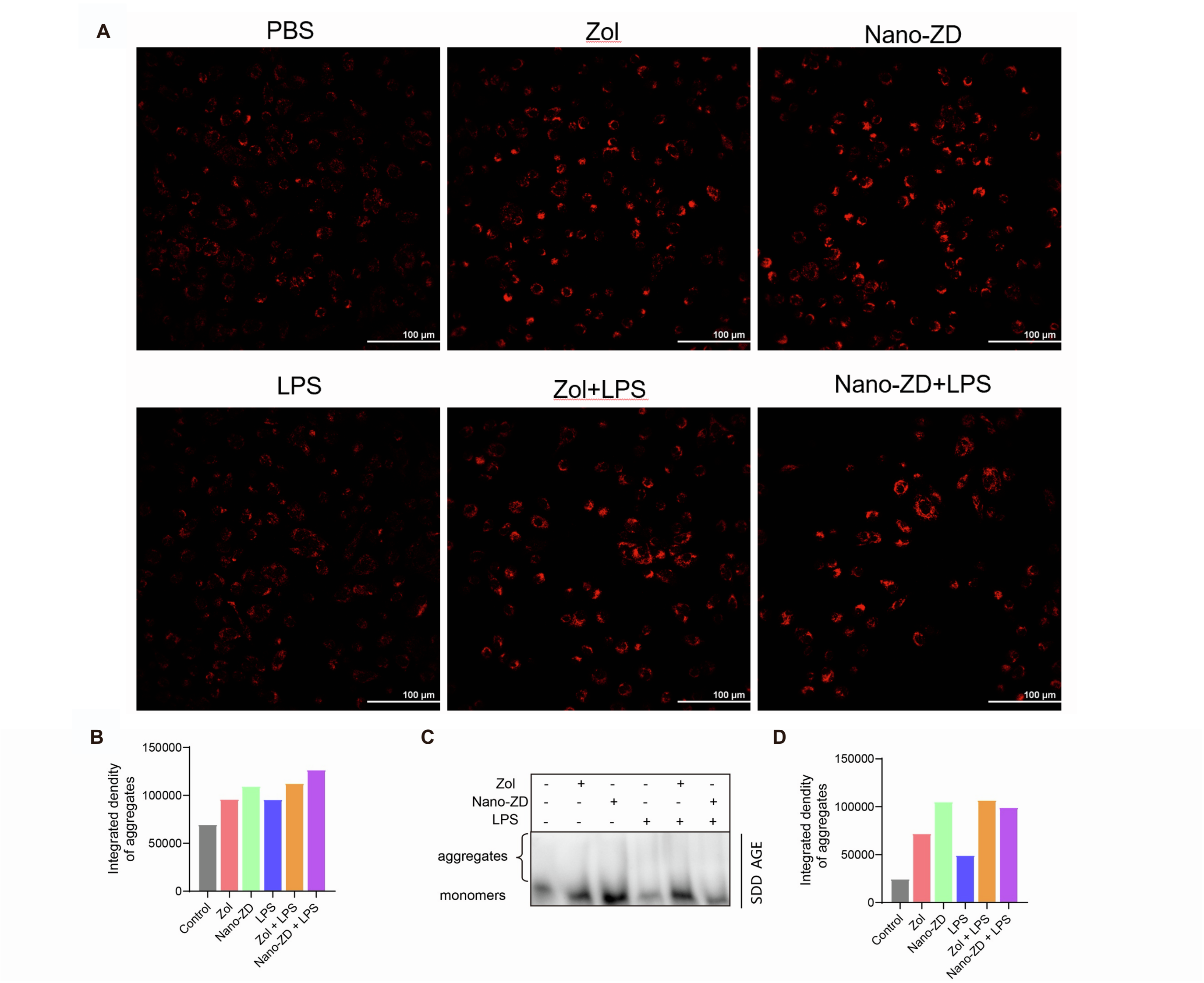

Figure S22. Immunofluorescence and SDD-AGE analyses of MAVS after different treatments. (A) Representative confocal images of MAVS staining in BMDCs. Related to Figure 6K. Scale bar, 100 μm. BMDCs were pretreated with zoledronate or Nano-ZD for 24 h and subsequently stimulated with LPS for 12 h. Scale bar = 100 μm. **(B)** Quantitative densitometric analysis of MAVS aggregates in Figure 6N. **(C-D)** Repeat experiment for SDD-AGE analysis of MAVS oligomerization and quantitative densitometric analysis.

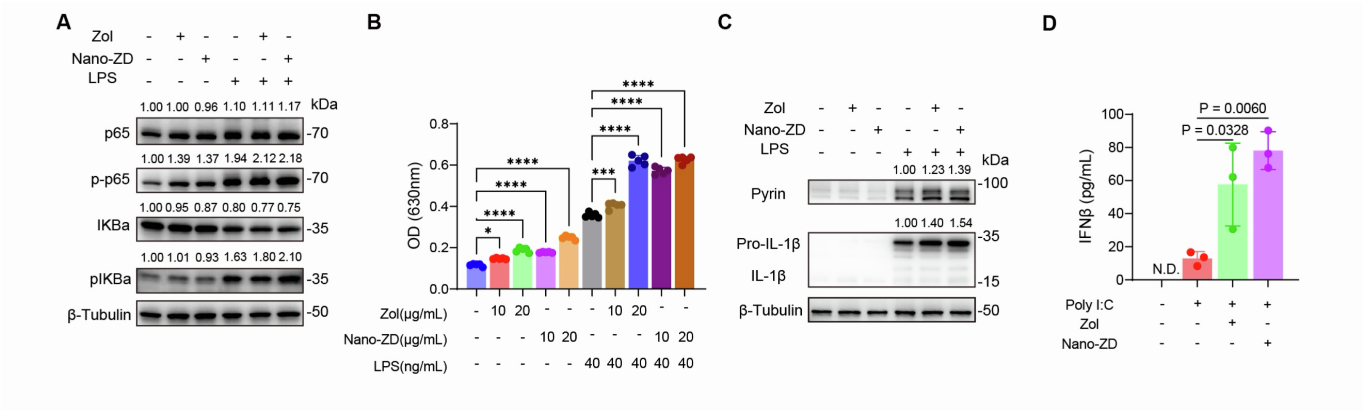

Figure S23. (A) Western blot analysis of total p65, p-p65, pIkBα, IkBα protein expression in the lysates of BMDCs after different treatments. β-tubulin was used as a loading control. **(B)** NF-κB agonistic activity of various treatments via a NF-κB reporter cell assay (n = 5). **(C)** Western blot analysis of pyrin and pro-IL-1β protein expression in the lysates of BMDCs after different treatments. β-tubulin was used as a loading control. **(D)** ELISA detection of IFNβ in the supernatant of BMDCs after treatment with poly I:C alone or after pretreatment with zoledonate and Nano-ZD followed by stimulation with poly I:C (n = 3). Related to Figure 6. Data are shown as mean ± s.d. * $P < 0.05$, ** $P < 0.01$, *** $P < 0.001$, **** $P < 0.0001$

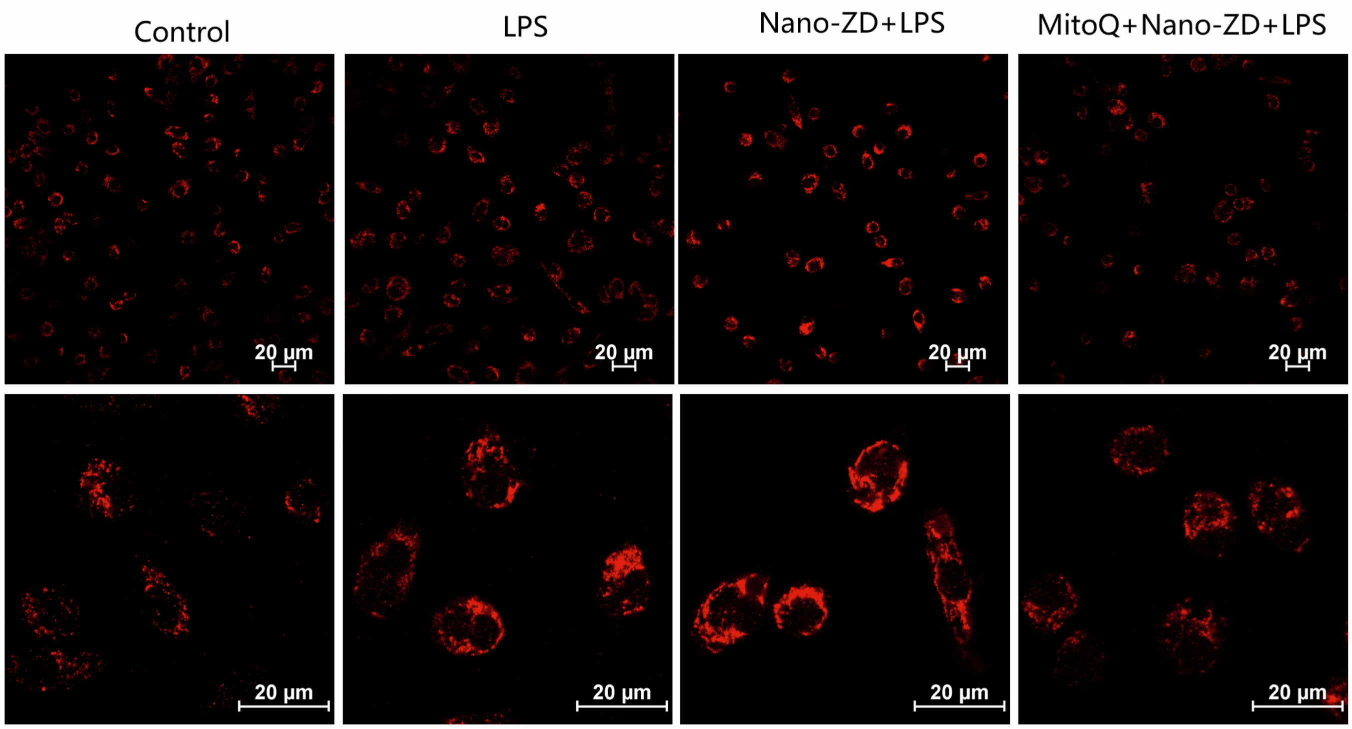

Figure S24. The representative confocal images of BMDCs after immunofluorescence staining for MAVS. Related to Figure 6O. BMDCs were pretreated with Nano-ZD for 24 h cells and subsequently stimulated with LPS for 12 h. MitoQ was added 30 min before the addition of Nano-ZD. Scale bar = 20 μm.

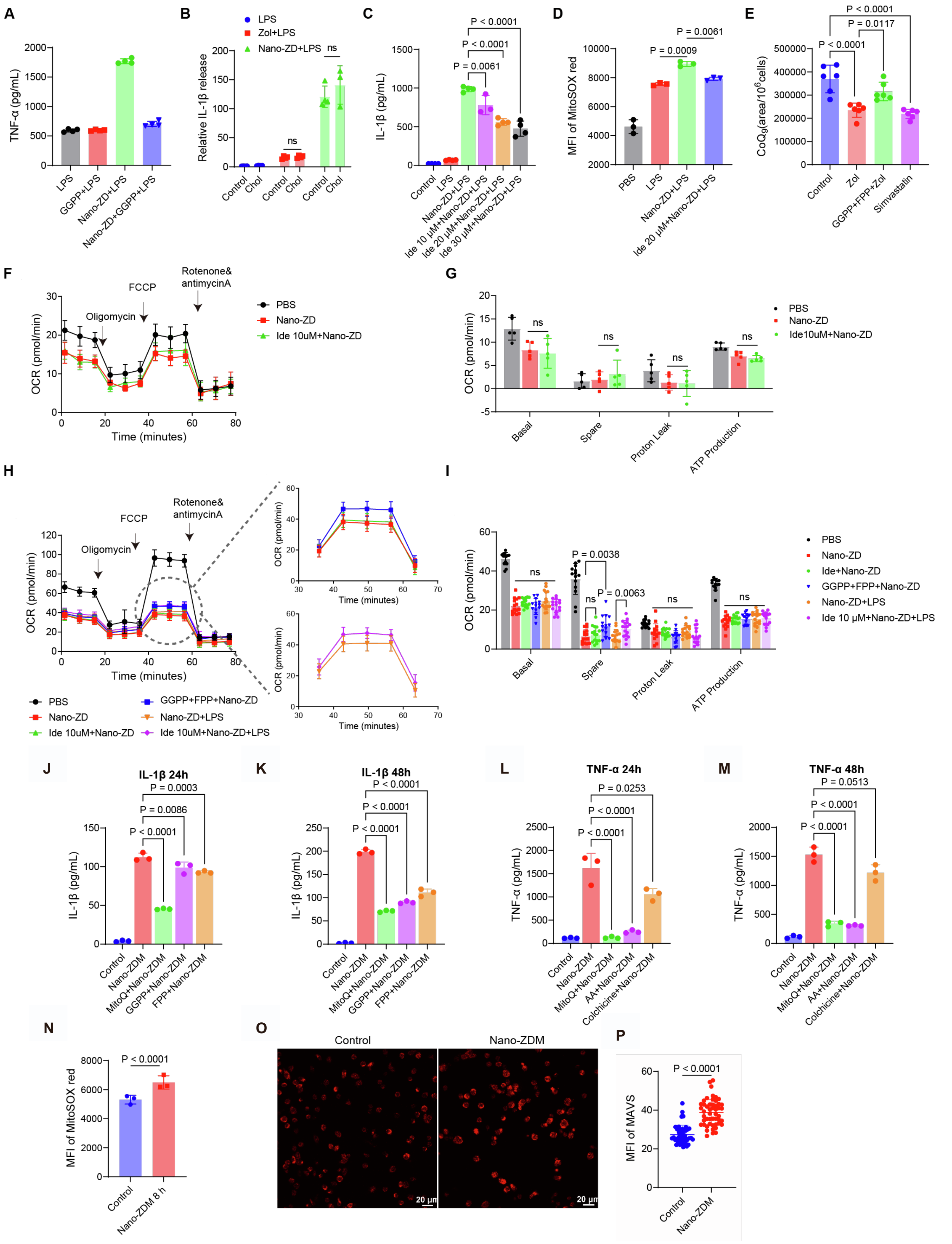

Figure S25. Nano-granulated zoledronate induces mtROS production through the mevalonate-CoQ-OXPHOS/pyrimidine metabolism axis. Related to Figure 7. (A) ELISA detection of TNF-α in the supernatant of BMDCs after supplementation with GGPP (n = 4). **(B)** Relative IL-1β release after supplementation with cholesterol (n = 3). **(C)** ELISA detection of IL-1β in the supernatant of BMDCs after supplementation with different concentrations of idebenone (n = 4). **(D)** The expression of mtROS in BMDCs after supplementation with idebenone (n = 3). **(E)** The CoQ9 levels in whole cells of BMDCs after different treatments (n = 6). **(F-I)** Mitochondrial stress test of BMDCs after supplementation with idebenone (10 μM), GGPP(5 μM) and FPP(10 μM).

(F-G) The real-time OCR change curve and the corresponding statistical results of basal respiration rate, spare respiratory capacity, proton leak, and ATP production (n = 5). **(H-I)** The real-time OCR change curve and the corresponding statistical results of basal respiration rate, spare respiratory capacity, proton leak, and ATP production in another experiment (n = 14 -16). **(J-P)** The mechanism studies of Nano-ZDM. **(J-K)** ELISA detection of IL-1β in the supernatant of BMDCs after supplementation with MitoQ, GGPP or FPP for 24 h or 48 h (n = 3). **(L-M)** ELISA detection of TNF-α in the supernatant of BMDCs after supplementation with MitoQ, arachidonic acid (AA), or colchicine for 24 h or 48 h (n = 3). **(N)** The expression of mtROS in BMDCs after Nano-ZDM treatments (n = 3). **(O-P)** Representative immunofluorescence staining images and the mean fluorescence intensity of MAVS in BMDCs after Nano-ZDM treatments. (Scale bar: 20 μm, each counted point represents one cell). Data are shown as mean ± s.d.

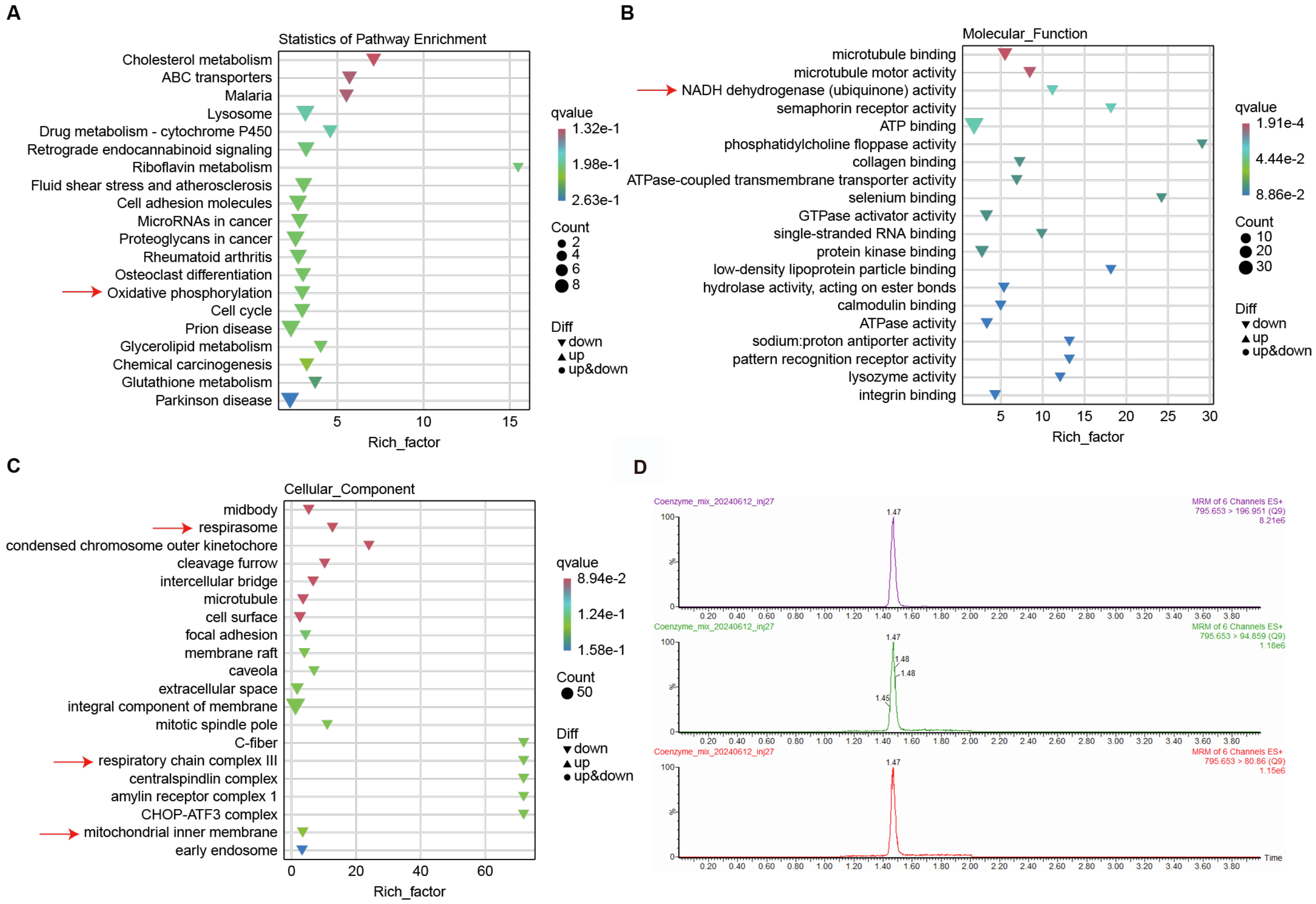

Figure S26. (A-C) RNA-seq analysis of BMDCs after the treatments of zoledronate. Related to Figure 7F. **(A)** KEGG enrichment analysis of downregulation of DEGs between untreated and zoledronate groups. **(B-C)** GO enrichment analysis in molecular function and cellular component of downregulation of DEGs between untreated and zoledronate groups. **(D)** Separation and detection of CoQ9 by LC-MS/MS. Related to Figures 7D and 7E.

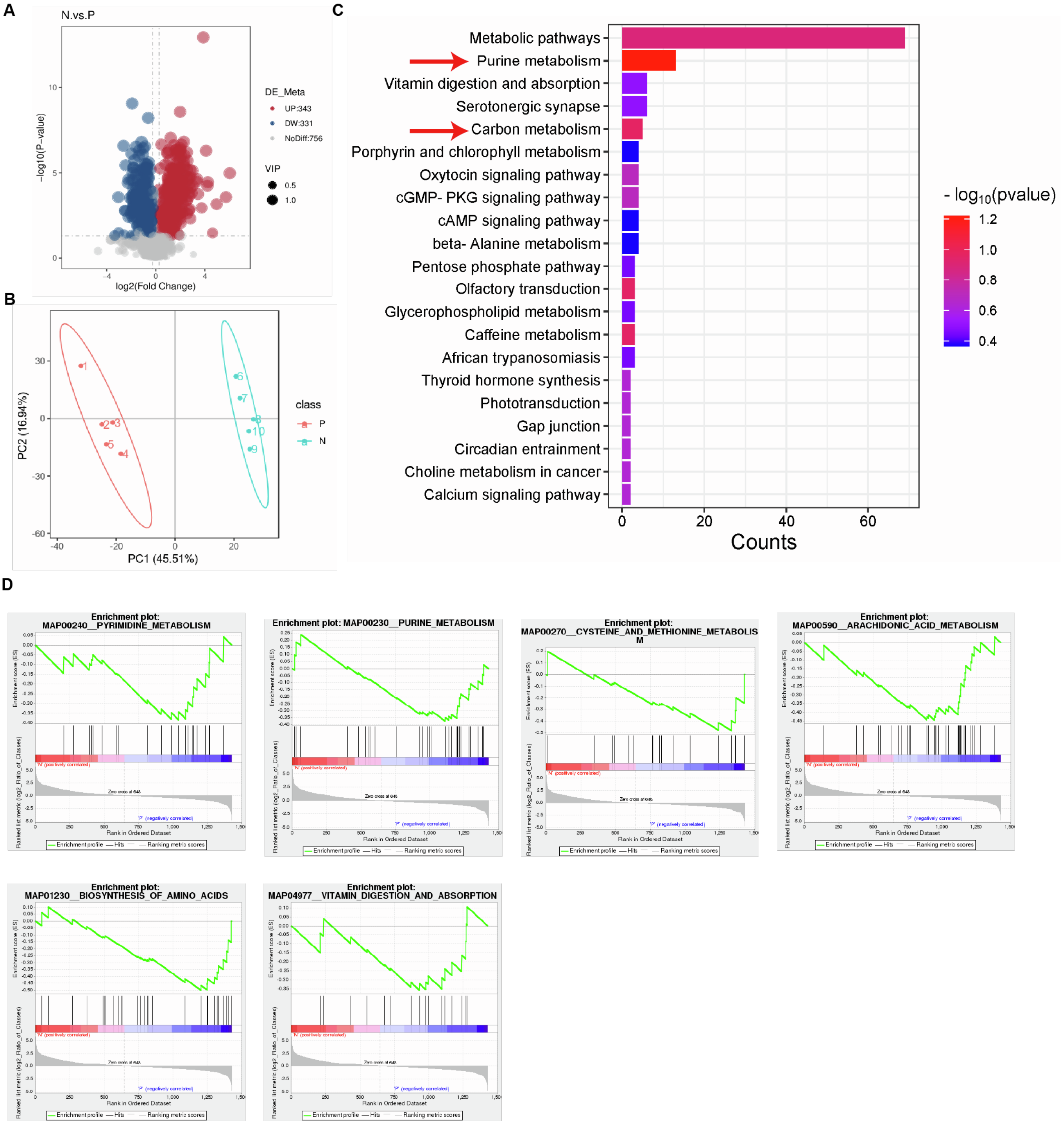

Figure S27. Analysis of untargeted metabolomics data of BMDCs treated with or without Nano-ZD (10 μg/mL zoledronate) for 24 h. Related to Figures 7K-7M. The control group is PBS (P), the experimental group is Nano-ZD (N). n = 5 **(A)** Volcano plots comparing metabolomic profiles of BMDCs treated with or without Nano-ZD. **(B)** PCA score plots of different groups. **(C)** KEGG enrichment analysis of differential metabolites between Nano-ZD and PBS groups. **(D)** Transcript Gene set enrichment analysis (GSEA) of differential metabolites between Nano-ZD and PBS groups.

Table S1. Diameter, zeta potential and drug loading efficiency of nanoparticles. Related to Figure 2.

	Core diameter (Number)/nm	Hydrodynamic diameter (Number)/nm	PDI	ξ-potential mV	the loading efficiency of zoledronate
Nano-ZD	32.2 ± 2.0	53.2 ± 1.6 nm	0.253 ± 0.0091	-7.4 ± 0.3	62.67 ± 2.51%
Nano-ZDM		55.2 ± 1.6 nm	0.246 ± 0.012	-9.8 ± 0.3	68.59 ± 2.14%
